# Bio-mordants: a review

**DOI:** 10.1007/s11356-024-32174-8

**Published:** 2024-02-23

**Authors:** Hüseyin Benli

**Affiliations:** Department of Chemistry and Chemical Processing Technologies, Mustafa Çıkrıkçıoğlu Vocational School, Kayseri University, 38280 Kayseri̇, Turkey

**Keywords:** Bio-mordant, Natural dyeing, Green production, Eco-friendly, Sustainability

## Abstract

Due to the increasing pressure on environmentally friendly approaches and sustainable production processes, the textile dyeing industry has focused on natural colorants. Thus, the use of bio-mordants, which are biological materials, has become widespread as an alternative to metal salts, most of which are non-ecological, used in the application processes of natural colorants. In natural dyeing, dyers want to use mordant substances in the dyeing processes in order to both expand the color spectrum and improve the fastness properties. Conventional metal salts used in natural dyeing are made up of metallic ions, which, when released into the environment as wastewater effluent at the end of the dyeing process, cause major damage to the ecosystem. Many researchers have thought about using mordants derived from natural sources to address the environmental problem. This article is a review of the investigation of natural mordants used instead of metallic mordants in the process of coloring various textile materials with natural dyestuff sources. It has been determined that many substances, most of them herbal materials, are used as mordants. In this review, mordants, except for conventional metal salts, are examined under three main groups for a better understanding. These groups are as follows: (*i*) natural or bio-mordants, (*ii*) oil mordants, and (*iii*) new-generation and non-vegetable-based mordants. Here, researchers will find an overview of the most recent developments in green mordants as well as application techniques for a variety of mordants.

## Introduction

The use of dangerous substances has come under intense scrutiny in the textile finishing industry, particularly because of the mutagenic, carcinogenic, and allergic consequences of textile chemicals and textile dyestuffs (Pereira and Alves 2012). Research in this area has concentrated on environmentally friendly, economically viable, and sustainable production techniques. The majority of textiles are colored and printed with petrochemical-based synthetic dyes. However, the effects of synthetic dyes on the environment, their chemical composition, and their contents are constantly questioned and criticized nowadays. As a result, researchers, designers, artists, and practitioners are interested in environmentally friendly alternative natural dyes and techniques for all natural fibers and the majority of man-made fibers. Natural dyes were widely used before the invention of synthetic dyes, frequently in conjunction with mordants like alum to dye natural fibers like wool, linen, cotton, and silk, but their use decreased after the invention of synthetic dyes. However, due to growing pressure on manufacturers to provide more environmentally friendly substitutes for dyes derived from petrochemicals, interest in natural dyes has been rekindled in recent years. Because some natural dyes have little to no affinity for textiles, heavy metal salts are used as mordants to fix the color and provide color fastness. The processes of pre-, simultaneous/meta-, and post-mordanting can all be used for natural dyeing.

In order to increase the affinity of natural dyes for textile fiber, substances called tannins could also be used. Tannins can improve the affinity of various dyes, making them useful as natural mordants in the process of natural dyeing. The phenols included in tannins’ hydroxyl group are crucial for creating crosslinks with different dyes and fibers, which helps to fix color (Pisitsak et al. 2018). Tannins are phenolic compounds found in nature. Those with o-dihydroxy groups convert to metal chelates when the substrate is pre- or post-treated with metal salts. The most popular and often used natural fiber is cotton. However, effective dye uptake is challenging due to the electrostatic repulsion between the anionic cellulose structure and natural colors (Pisitsak et al. 2016). Tannin-containing mordants were discovered to be effective when applied to cotton fibers because they improved the way the dye adhered to the fabric (Prabhu and Teli 2014). Plants with high concentrations of tannin and chlorophyll have been used successfully as bio-mordants in the natural dyeing of textiles (Singh et al. 2021). Oak bark and wood (*Quercus infectoria*), pomegranate peel (*Punica granatum*), and cutch (*Acacia catechu*) are a few crucial raw materials for tannins (Pisitsak et al. 2016, 2018). In one study, *Moringa* and neem bark, rich in tannic acid and catenoids, were used as mordant agents in dyeing wool with cocoa fibers (Jabar et al. 2023b). In traditional natural dyeing, some metal salts are also preferred to increase affinity. In Europe, alum has been well-known for ages. Filings made of iron and tin have also been employed. In Scotland and Ireland, stale urine is frequently employed, though perhaps more as a cleaning agent than as a true mordant (İşmal and Yıldırım 2019). Today, many researchers still use metal salts such as iron, crom, tin, alum, zinc, and copper as mordants in their natural dyeing studies (Davulcu et al. 2014; Bahtiyari et al. 2020; Benli 2017, 2021, 2022a; Benli and Bahtiyari 2018, 2023, 2022b). Natural dyeing has expanded beyond its original uses in modern times. Particularly, the variety of natural mordants has been expanded to include diverse organic wastes, which have begun to be directly supplied from nature (İşmal and Yıldırım 2019).

In this review, detailed research on the biological-based mordants that scientists use instead of metal mordants in their academic studies has been made and presented to the readers. It has been determined that many plant materials can be used as sources of bio-mordants instead of metal mordants in natural dyeing processes. New dimensions and ideas for mordant classification have consequently emerged.

## Material and methods

In this review, the most up-to-date version of natural mordant materials that will help the dyeing process while using natural dyes, which will make serious contributions to environmentally friendly production processes, is presented. In the literature review, it was determined that dozens of different biological materials were used as natural mordant materials. During the literature review, a compilation was made using very different terms that can express natural mordant substances. The terms used in the search are “natural mordants,” “bio-mordants,” “oil mordants,” “eco-friendly mordants,” “green mordants,” “bio-fixing mordants,” “anchoring mordants,” and “safe mordants.” Websites used for comprehensive literature reviews include Web of Science, Scopus, Google Scholar, and EBSCO (Textile Technology Complete Database). The related reports that covered the specific use of bio- or natural mordants on textiles were chosen.

## Mordant agents

Especially in recent years, different expansions have been made in the coloring of textile materials. At the beginning of these expansions is the use of plant-based natural dyestuffs in dyeing processes. Traditionally, metal salts, called mordants, are frequently used in dyeing processes with natural dyestuffs. The Latin word “mordere,” which means “to bite,” is where the word “mordant” originates (Cunningham et al. 2011; Prabhu and Bhute 2012). A mordant is a substance that can be fastened to a fiber and joins chemically with natural colorants. Depending on how they are used, natural dyes can be divided into two categories: substantive dyes and non-substantive dyes. The fabric does not need to be pretreated before using the substantive dyes (e.g., indigo, orchil, and turmeric). Contrarily, non-substantive dyes (such as logwood, madder/alizarin, cochineal, or fustic) can only color already-mordanted materials or function when a mordant is added to the dyebath. Pretreatment comes in three different forms: direct (for cotton, e.g., turmeric, safflower); acid (for silk and wool, e.g., saffron, lac); or basic (for silk and wool, e.g., berberine). Natural mordant dyes can be either monogenetic or polygenetic; the former produces only one color regardless of the mordant used, while the latter produces different colors (e.g., logwood, alizarin, fustic, and cochineal). According to their chemical structures, natural dyes are divided into the following basic groups: turmeric (diarylol methane), saffron, annatto (carotenoid), barberry (alkaloids), henna (quinonoid), French marigold, sandal (flavonoid), safflower (benzoquinone), alroot, and oyam (anthraquinone) (Roy Choudhury 2013).

Because most natural dyes do not have a strong affinity for textile fibers, especially cellulosic, mordanting is a step that must be added to the dyeing process. Cotton needs to be mordanted since it is harder to dye than wool or silk because it lacks the amino- and carboxyl-groups that serve as attachment sites for dye molecules (Saxena and Raja 2014). As a result, transition metal salts, often known as mordants, are employed in natural dyeing. Because outcomes vary depending on the plant and the type of mordant, it is impossible to offer strict rules and instructions for the mordanting process. To improve the dyeing qualities, mordants bond the dye to the fabric and alter the pH of the medium. The majority of natural dyes require a mordant agent to fix the color to the fiber and increase its fastness. It is seen that mordants are divided into different classes in the literature. Saxena and Raja (2014) divided mordants into three groups: metal salts or metallic mordants, oil mordants, and tannins. Adeel et al. (2017a) classified mordants as basic mordants (chemical mordants), acidic mordants (bio-mordants), and newly discovered mordants. In another study, mordants were divided into three classes as metal salts or metallic mordants, tannic acid (tannins), and oil mordants (Prabhu and Bhute 2012).

In this review, mordant substances are examined in three subclasses: natural or bio-mordants, oil mordants, and new-generation and non-vegetable-based mordants. Conventional mordant substances (metal salts) are mentioned here briefly but will not be discussed in detail.

### Natural or bio-mordants

Bio-mordant agents are used in the same way that metallic mordants are. Table 1 presents the bio-mordant varieties that have been used so far, and some of them are as follows: oak galls, oak wood, gallnut, sumach, myrobalan, pomegranate rinds, tannin, tannic acid, tartaric acid, guava, banana leaves, ash, valex, rosemary, thuja, amla, roots of Rumex hymenosepalus, chlorophyll, green tea, black tea, chitosan, lemon juice, eggshell, natural lodhra, kenduka, gelatine, albumen, acetic acid, caseine, lactarane, milk, urine, blood, and bentonite. Bio-mordants are powerful biomolecules that increase the functional properties of the plant when interacting with substances such as cotton, wool, and silk (Hosseinnezhad et al. 2022a). When these molecules are applied to the material before or after dyeing, it produces solid and stable tones via the extra H-bond (Jabar et al. 2022). In addition, the conjugation in the molecule plays a role in strong, unfading bonds by causing a special bond with the functional region of the material (-NHCO = silk or wool; -OH = cellulose) and the colorant functional points (-O.H. or -O.H. and -C = O) by the transfer of ions in the system (Botteri et al. 2022). In this review, lots of herbal sources have been identified that are used as bio-mordant agents. But, herein, the photographs of the plants belonging to the only ten most cited bio-mordans by the researchers are given in Table 1. In natural dyeing processes between 1993 and 2023, and especially in the last 5 years, 71 studies on pomegranate peel, 51 studies on *Acacia*, 47 studies on tannic acid, 45 studies on myrobalan, 35 studies on turmeric, 22 studies on oak, 21 studies on henna, 14 studies on lemon, 12 studies on tamarind, and 12 studies on *Aloe vera* plants were found to be among the most used bio-mordant substances. Additionally, more than 80 plant sources have been identified by researchers as being used as bio-mordants in natural dyeing. In Table 1, an attempt is made to establish a relationship between the plant sources used as bio-mordans and the authors who used these bio-mordans.

**Table 1:**
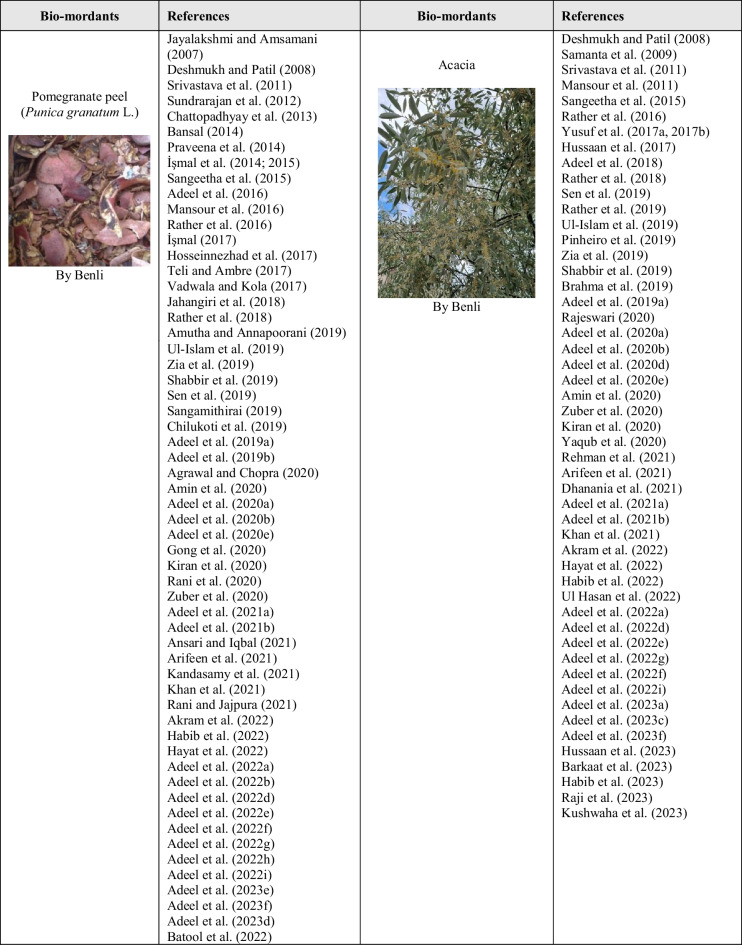

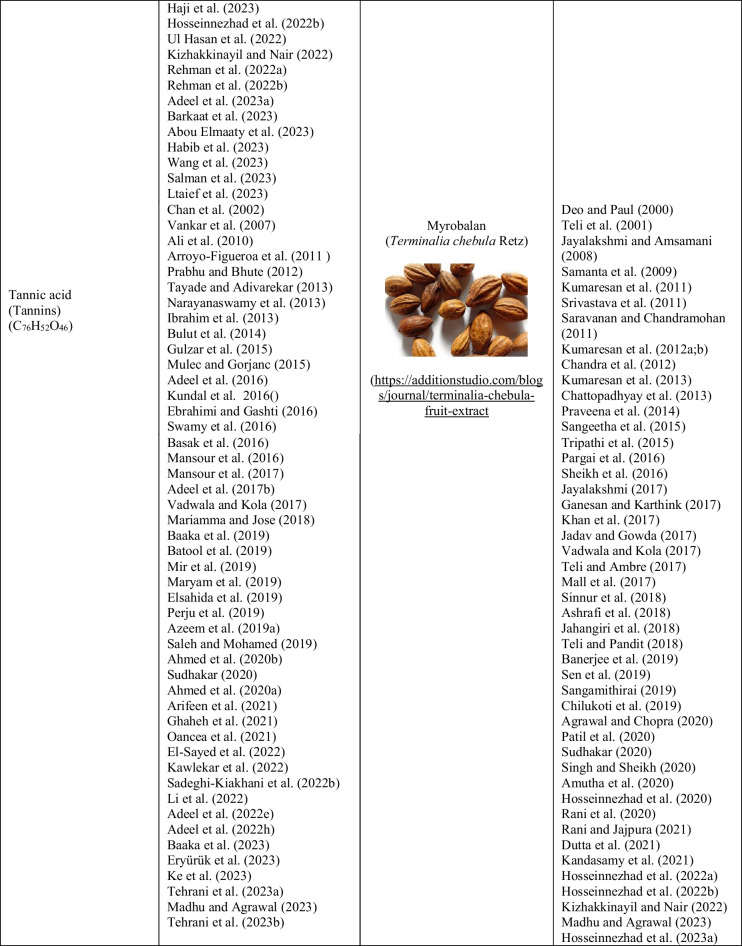

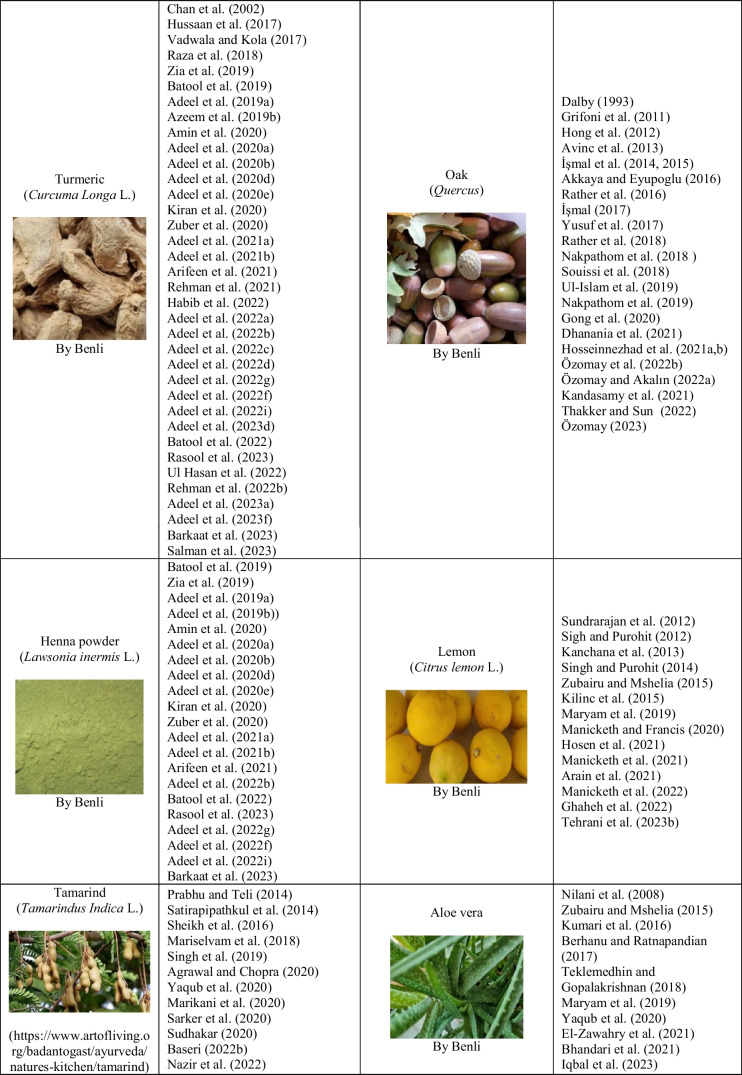

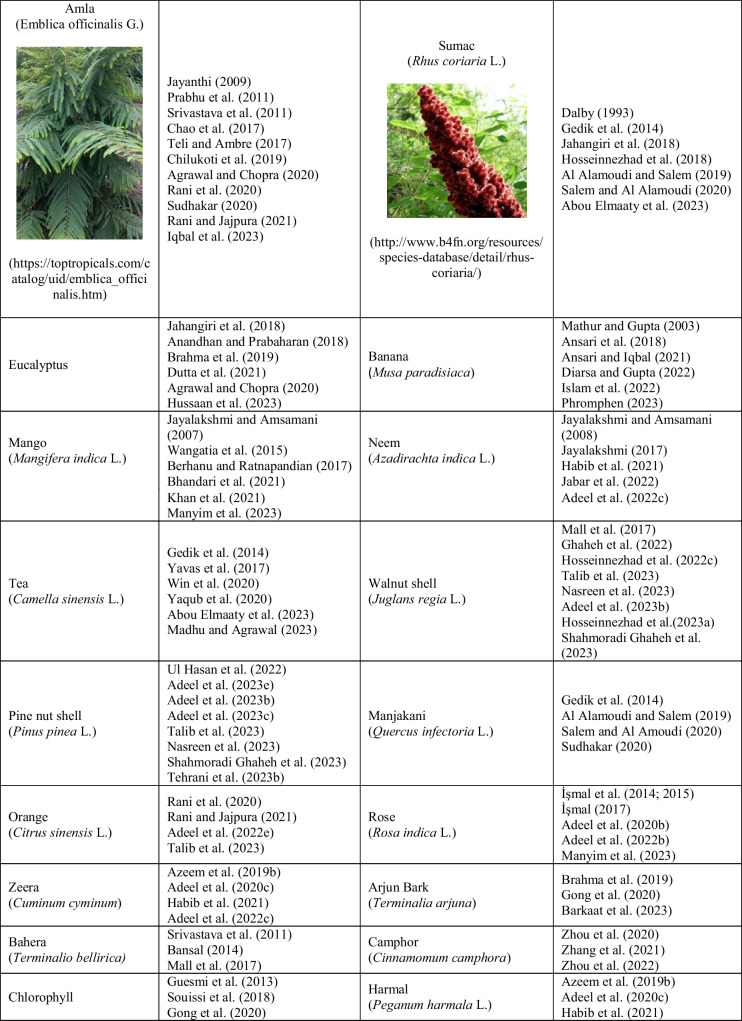

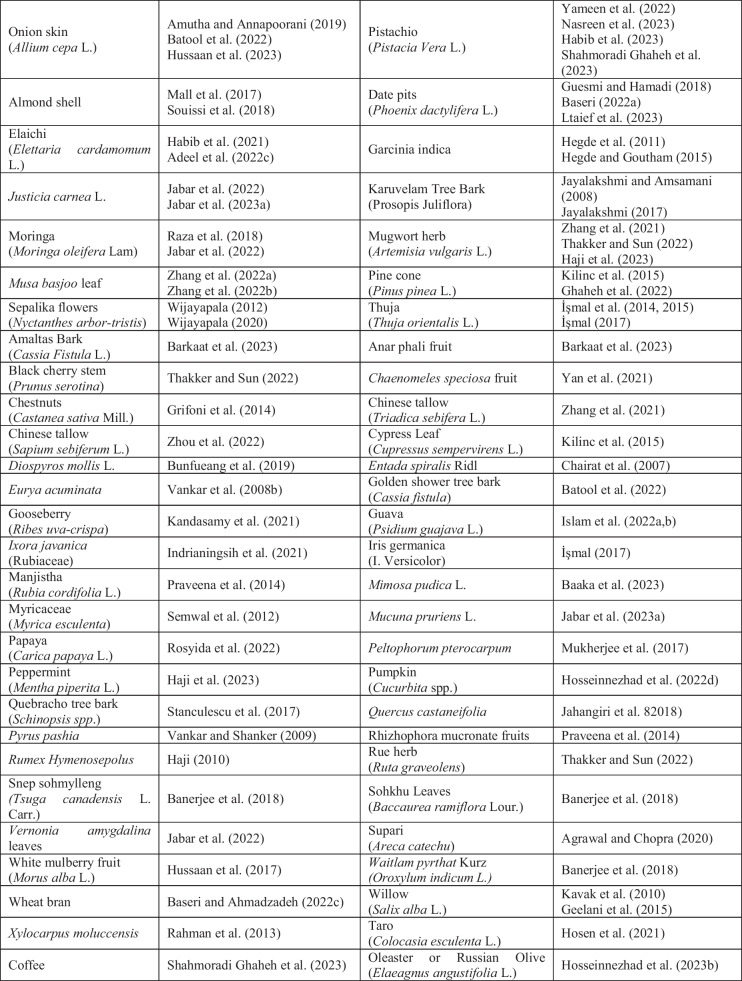
Sources used as bio-mordants in the literature

As well, a few examples of natural mordants include cupula of oak, unripe grape juice, vinegar, Seville orange juice, spurge secretion, ox urine, rock algae, clay, lime, bread yeast, wood ash, and mud mixed with animal urine (Ozturk et al. 2013). In Anatolia, natural mordants such as salt, lemon salt, cattle urine, vinegar, sour grapes, citrus juice, washing soda, mole milk, baker’s yeast, ash, clay, pelit, moss, dried yogurt, and lime were used in dyeing processes from time to time in various proportions (Başaran and Sarikaya 2015). Bio-mordants are included in the keywords of many articles. Academic studies using bio- or natural mordant materials are listed alphabetically and briefly summarized below.

#### Acacia

According to current knowledge, there are more than 1350 species of *Acacia* throughout the world. There are at least five different groupings of *Acacia* species, according to a recent study on the genus. *Acacia* species are challenging to categorize and identify. Several *Acacia* species have been reported to contain amines, alkaloids, gums, non-protein amino acids, terpenes (including essential oils, diterpenes, phytosterol and triterpene genins, and saponins), hydrolyzable tannins, flavonoids, and condensed tannins (Seigler 2003). Numerous scientists have employed the *Acacia* plant as a bio-mordant in their natural dyeing investigations. Silk fabrics were dyed using *Acacia* extract as a bio-mordant and *Sticta coronata* as a natural dye by Mansour and Heffernan (2011). Based on the outcomes, it is stated that the *Sticta coronata* dye gave silk cloth a lovely violet hue. In a different study, gallnut, pomegranate peel, and *Acacia arabica* bark tannin extracts were utilized as bio-mordants to color wool. It was announced that the results of using bio-mordants in wool dyeing are comparable to those of metallic mordants in terms of color strength and fastness properties, that bio-mordants produce quite different color gamuts than expected from a mordant and thus offer the potential to replace metal salts in the wool dyeing process (Rather et al. 2016). In different experiments, *Acacia* was used as a bio-mordant when dyeing wool and cotton with natural dyes. According to the results obtained, it is stated that shades of red wool with good to excellent color fastness properties were obtained (Yusuf et al. 2017a, b). Ul-Islam et al. (2019) proposed that by utilizing bio-mordants isolated from pomegranate peel (*Punica granatum* L.), gallnut (*Quercus infectoria* L.), and catechu (*Acacia catechu*), wool samples may be dyed in a sustainable and metal-free manner. As a result of the analysis, the examined bio-mordants interact differently with the coloring components of *Butea monosperma* (palas) dye, producing dark brown, olive green, dark brown, cinnamon, burgundy, and yellowish tones on wool. Zia et al. (2019) employed henna leaves (*Lawsonia inermis*), turmeric rhizomes (*Curcuma longa*), pomegranate bark (*Punica granatum*), and *Acacia* bark (*Acacia nilotica*) as bio-mordants to color cotton fabric. With regard to the findings, it is stated that ultrasound treatment, a green technique for natural dyeing, offers a lot of potential for isolating colorants from plant sources in friendly circumstances and has acceptable fastness. In a different study, wool yarn was dyed naturally with lac and Himalayan rhubarb, and a bio-mordant was created using bark extract from the *Acacia nilotica* plant. According to the findings, it was determined that the developing color was in the yellow–red coordinate of the color space diagram, and the color tones changed toward the red region in *Kerria lacca* stained samples and toward the yellow region in *Rheum emodi* stained samples (Rather et al. 2019). Pinheiro et al. (2019) employed *Acacia mearnsii* sawdust as a bio-mordant and *Hibiscus sabdariffa* flowers, *Allium cepa* peels, and *Curcuma longa* root as natural dyes to color natural fibers. Based on the results, pleasant changes in fiber color were seen in all samples due to the nuances produced by each natural dye, but dyeings with extracts of *H. sabdariffa*, *A. cepa*, and *C. longa* showed greater impregnation of *A. mearnsii* and showed intense pink, mustard yellow, and yellow colors, respectively. It caused reddish-brown discoloration. In another study, pomegranate peel and *Acacia* were utilized as bio-mordants to dye wool, while *Alkanna tinctoria* roots were employed as a natural dye. According to the results, a wide range of colors were produced with good color quality and fastness results, and pomegranate peel extract, iron, and babool, among other bio-mordants, improved the color yield of dyed wool (Shabbir et al. 2019). Sen et al. (2019) used lac as a natural dye to dye polyester fiber and catechu, myrobalan, and pomegranate as bio-mordants. In accordance with the outcomes, it is claimed that the color strength obtained with catechu as a bio-mordant was higher than with stannous chloride and comparable to that with ferrous sulfate. To color nylon fabric, Rehman et al. (2021) employed lac insects as a natural dye and *Acacia* and turmeric as bio-mordants. In accordance with the findings, there is a claim that the pre-mordanting approach produces the best fastness qualities, and the highest color yields when 5% of *Acacia* and 1% of turmeric are added. Arifeen et al. (2021) used *Acacia* bark, turmeric, henna, pomegranate, and tannic acid as bio-mordants to color nylon fabric. They also employed walnut bark as a natural dye. Subject to the findings, it is stated that in comparison to their synthetic counterparts, it has been found that using 3% of turmeric extract as a pre-bio-mordant and 5% of *Acacia* extract as a post-bio-mordant has produced good color qualities, and it is also determined that the incorporation of sustainable biosources as a color modifier has added value to the natural dyeing process and improved the color ratings of the reddish-brown dye obtained from walnut bark by ultrasonic treatment, an environmentally friendly instrument. In a further experiment, *Ficus religiosa* bark powder was used as a natural dye to dye cotton, and *Acacia* and pomegranate were used as bio-mordants. Depending upon the findings, it is stated that 2% of pomegranate and 4% of *Acacia* extracts used as a post-bio-mordant have produced good color qualities (Akram et al. 2022). Peepal (*Ficus religiosa*) was used by Habib et al. (2022) as a natural dye to color silk, and *Acacia*, turmeric, and also pomegranate were used as bio-mordants. With regard to the findings, it is a claim that the use of bio-mordants has improved the sustainability and environmental friendliness of the process while also achieving the best *K/S* and L*a*b* values. In one study, Hayat et al. (2022) employed *Acacia* and pomegranate as bio-mordants and black tea leaves as a natural dye to dye silk fabric. Based on the findings, it is stated that new color shades with good to outstanding fastness degrees were produced by using iron (3%) and *Acacia* extract (2%), iron (2%), and pomegranate extract as precursors and bio-mordant (2%) were used as post-chemical and bio-mordant, while aluminum (3%) and pomegranate extract (3%) were used as meta-chemical and bio-mordant. In different studies, Adeel et al. used pomegranate (*Punica granatum*) rind, *Acacia* (*Acacia nilotica*) bark, and turmeric (*Curcuma longa*) rhizomes as bio-mordants to dye wool and cotton. Depending upon the findings, it is claimed that applied to MW-treated cotton at 55 °C for 75 min, 50 mL of acidified methanol-solubilized extract from a 5-min microwave-treated powder produced excellent results; additionally, the use of salt of Fe as a chemical mordant and extracts of *Acacia* and turmeric as bio-mordants under ideal conditions has produced good to excellent fastness ratings; microwave treatment also has good potential for the extraction of the colorant from Arjun bark for cotton dyeing and its application (Adeel et al. 2022a, d, 2023f). In one work, Raji et al. (2023) exploited *Reseda luteola* L. for the extract of a natural yellow dye, using the tannic bark of *Acacia mearnsii* as a bio-mordant to ensure sustainable dyeing of wool fabrics. Subject to the findings, it is stated that pre-mordanting with an alternative bio-mordant (alum-*Acacia mearnsii* tannin) enhanced the fabric’s general ability to absorb dyes, generating a spectrum of hues from pale yellow to dark brown while also exhibiting outstanding resistance to washing and wet and dry rubbing. In one study, Kushwaha et al. (2023) reported that to dye pineapple fabric with coral jasmine natural dye using *Acacia nilotica* (babul bark) as a bio-mordant, the results demonstrated that babul bark and coral jasmine concentrations boosted color yield (*K/S*). The wash and rubbing fastness qualities were good to exceptional. The fabric sample with the highest dye and mordant content (5% owf babul shell and 20% owf coral jasmine) showed antibacterial activity up to 98.23%, antioxidant activity up to 99.37%, and outstanding UV protection. It has been mentioned that there is a complex structure between the bio-mordan, the dye source, and the fiber.

#### Almond shell

Green almond shells, which contain chemical structures such as tannin and gallic acid, were used to strengthen dyes and make them resistant to chemical treatments (Vankar and Shanker 2008a; İşmal et al. 2014). Souissi et al. (2018) used three different metallic (Alun, ZnSO_4_, and CuSO_4_) and biological (gall nuts, chlorophyll (a), and green almond shell) mordants to improve the absorption degree as well as the color fastness of cotton fabrics dyed with the aqueous extract of dates. According to the results obtained, they determined that the color fastness values of dyeings made using biological mordants were higher than those made using metal mordants.

#### Aloe vera

People have recognized and used the *Aloe vera* plant for thousands of years because of its advantages for their health, looks, and skin. The name *Aloe vera* is derived from the Arabic word “Alloeh,” which means “shining bitter stuff,” and the Latin word “vera,” which denotes “truth.” The botanical name of the plant that yields *Aloe vera* is *Aloe barbadensis Miller*. It belongs to the Liliaceae family and is a perennial, xerophytic, pea-green, shrubby, or arborescent plant. It primarily grows in the arid climates of Africa, Asia, Europe, and America. *Aloe vera* contains 75 potentially active substances, such as vitamins, enzymes, minerals, carbohydrates, lignin, saponins, salicylic acids, and amino acids. It provides 12 phenolic compounds called anthraquinones, which are widely employed as laxatives. Emodin and aloin have antiviral, antibacterial, and analgesic properties (Surjushe et al. 2008). *Aloe vera* gel has unique characteristics like colorlessness, transparency, and viscosity and can be used as a printing thickener, mordant, and antimicrobial for various fabrics and colors. *Aloe vera* is used in pre-treatments such as cleaning, desizing, softening, and printing due to its succulent, enzymatic, and sticky characteristics. A salty component of *Aloe vera* gel also allows for natural, eco-friendly coloring. *Aloe vera* gel is a natural antibacterial substitute for synthetic antimicrobials (Mosaad 2021). Several scientists employ the *Aloe vera* plant as a bio-mordant in their studies, according to the literature. In one study, depending upon the findings, it is stated that *Aloe vera* juice was utilized as a natural mordant, and marigold petals were used as a natural dye, and when used in a 10:10 ratio with *Aloe vera* juice, the natural dye from marigold petals had a good dyeing impact on animal fiber as opposed to plant or synthetic fiber (Nilani et al. 2008). In a study, both metallic and organic (*Aloe vera* and lemon) mordants were used to color cotton garments with a natural dye extract made from onion peel. Subject to the findings, it is stated that natural mordants gave pale yellow colors, while synthetic mordants such as copper sulfate and alum also gave yellow colors, and on the other hand, iron sulfate gave darker shades of color (Zubairu and Mshelia 2015). Kumari et al. (2016) evaluated the dyeing effects on synthetic fibers by using the chemicals produced from *Morus alba* bark as a natural dye and *Aloe vera* extract as a natural mordant. In a study, the potential for employing the extracts from the bark of the *Cassia singueana* plant as a natural dye and the extracts from *Aloe vera* and mango peels as bio-mordants in the coloring of tanned sheep skin was examined. The majority of samples showed that the fastness result fell between good and excellent (Berhanu and Ratnapandian 2017). Teklemedhin and Gopalakrishnan (2018) used *Aloe vera* extract as a bio-mordant for dyeing silk fabrics. When dyed directly without a mordant and when dyed with copper sulfate and *Aloe vera* mordants, the results for washing fastness, rubbing fastness, and light fastness were nearly identical and recorded in the very good to excellent range. Using natural dye produced from the *Cassia singueana* plant without the use of a mordant and dying in the presence of *Aloe vera*, the findings of this experiment led to the conclusion that it is possible to dye silk fabric with a range of acceptable fastness attributes. In a different investigation, gelatin and *Aloe vera* extracts were employed as bio-mordants for ecologically friendly dyeing of cotton fabric with a satisfactory color fastness value. The relationship between the bio-mordan, the dye supply, and the fiber is complex, as was already mentioned (El-Zawahry et al. 2021).

#### Amaltas (*Cassia fistula* L.) Bark

Amaltash is the popular name for *Cassia fistula*, a member of the Fabacae family. This plant is used in traditional medicine to treat leprosy, skin conditions, and syphilis, as well as burns, constipation, convulsions, diarrhea, dysuria, and epilepsy. A rich source of tannins, flavonoids, and glycosides can be found in *Cassia fistula* (Verma 2016). The extract of this plant was used as a bio-mordant in natural dyeing. Barkaat et al. (2023) stated that for bio-mordanting, turmeric rhizomes, henna leaves, *Acacia* bark, pomegranate rind, Amaltas bark, pomegranate peel, Anar phali fruit, and Arjun bark were used in the natural dyeing process. It is stated that before dyeing, the Arjun bark extract’s high strength (*K/S* = 25.04) and darker shade (L* = 22.3) with a reddish yellow color (a* = 28.1; b* = 31.9) were generally present, and the application of pomegranate extract, which contains tannin, also created high strength (*K/S* = 26.1) and a less red (a* = 12.1) but yellower color (b* = 29.6) following dyeing.

#### Amla (*Emblica officinalis* G.)

**Fig. 1:**
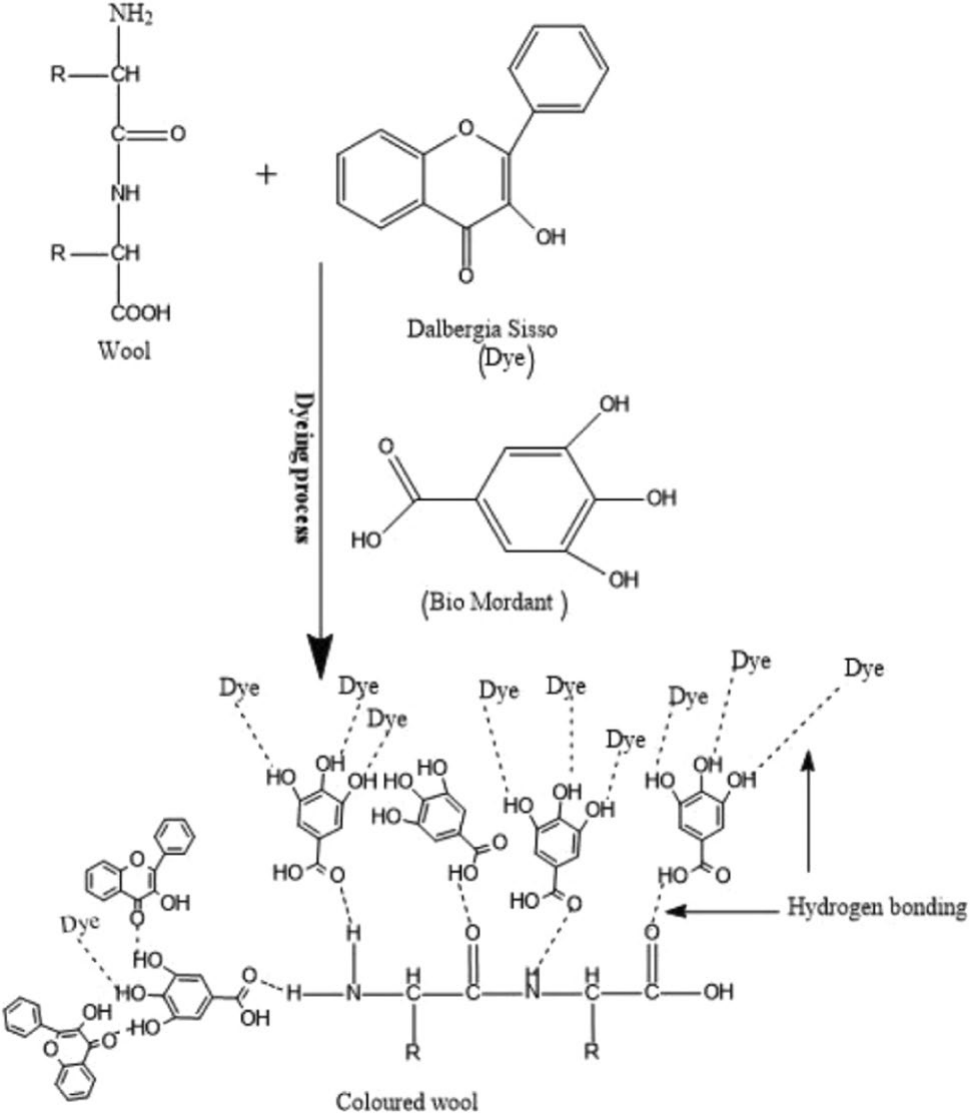
Complex structure formed between bio-mordant, wool, and dye (this figure was adapted from Iqbal et al. 2023)

The tree *Emblica officinalis* G., often known as amla in Hindi, belongs to the tiny genus *Emblica* and is indigenous to China, India, Ceylon, and India. *Emblica* is known to be a good source of flavones, tannins, and other bioactive substances. Tannin, which makes approximately 28% of the fruit of *Emblica officinalis* G, includes a phenolic hydroxyl group that can help colored molecules in natural fabrics take on their color. In other words, tannin can improve the way plant dyes bind to fibers, intensifying the color of fabrics dyed with plant dyes like cotton and silk (Prabhu et al. 2011). Listed below are numerous articles in which amla is used as a bio-mordant in natural dyeings. In a study, it was emphasized that the tannin found in the dried fruits of the *Emblica officinalis* plant can be a good mordant for dyeing cotton fabrics with direct dye (Jayanthi 2009). Prabhu et al. (2011) reported that tannin extracted from the dried fruits of *Emblica officinalis* can be used alone as a natural mordant and can also be combined with copper sulfate for dyeing cotton and silk fabrics. It is stated that the inclusion of *Emblica officinalis* G. tannin, which serves as a mordant and contains phenolic hydroxyl groups that form complexes with the dye molecules and increase the fixation of dye on cotton fabric, was responsible for the improvement in dyeing depth. In another study, it was reported that cotton and silk fabrics could be dyed using the natural mordant tannin extracted from amla using five different natural dye sources and copper sulfate, a metal mordant, and the *K/S* and UFP (ultraviolet protection factor) values of dyed fabrics increased significantly (Chao et al. 2017). In a different study, natural indigo and marigold were used as natural dyes, and amla, harda, and pomegranate rind were used as natural mordants in the coloring of cotton and cotton/viscose fabrics, and it was reported that natural mordants could be promising in the future (Teli and Ambre 2017). Chilukoti et al. (2019) stated that cotton fabrics can be dyed using the extract obtained from onion peel as a natural dye by using the Soxhlet device and using amla, myrobalan, pomegranate peel, and chitosan as natural mordants. It is stated that in the pre-mordanting procedure, chitosan was found to have the highest *K/S* values among the four distinct mordants, and the fastness characteristics of nearly all the samples were found to be average. In another study, it was stated that bio-mordants (harda powder, pomegranate peel, orange peel, and amla powder) can replace metal mordants in wool dyeing due to the dyeing of protein fabrics using natural tannin extracts as well as metal salts (ferrous sulfate, alum, and copper chloride). Dyed fabrics, especially wool, exhibited a very large enhancement in UV protection, and it is also stated that dye extract and dyed samples have very good anti-microbial behavior (Rani et al. 2020). In one study, Iqbal et al. (2023) stressed the use of bio-mordants like *Aloe vera* and *Emblica officinalis* (amla) to accomplish sustainable natural dyeing utilizing *Dalbergia sissoo* on wool. Results indicate that utilizing natural mordants improved the fixing of natural dyes and also enhanced their antibacterial activity. The complex structure between the bio-mordant, the dye source, and the wool is presented in Fig. 1.

#### Anar phali fruit

Mexico is the native home of *Opuntia ficus-indica* (L.), also known as the prickly pear, barbary pear, cactus pear, Indian pear, Indian fig, tuna fig, mission cactus, nopal, nopalito, amar phali, chittarthohar, and nagphani. Kaempferol, quercetin, kaempferol 3-methyl ether, narcissin, ( +)-dihydroxykaempferol (aromadenrin), ( +)-dihydroxyquercetin (taxifolin), and eriodictyol are among the flavonoids found in nopal fruit (Khan and Shaukat 2006). Anar phali fruit was used as a bio-mordant in natural dyeing. Barkaat et al. (2023) stated that for bio-mordanting, turmeric rhizomes, henna leaves, *Acacia* bark, pomegranate rind, Amaltas bark, pomegranate peel, Anar phali fruit, and Arjun bark were used in the natural dyeing process.

#### Arjun bark (*Terminalia arjuna*)

*Terminalia arjuna* (family Combetaceae), a versatile medicinal plant with a wide range of biological activities and a large-sized fluted tree with a usually buttressed trunk, is found in the South Asian region. The presence of active substances, such as phytosterol, lactones, flavonoids, phenolic compounds, tannins, and glycosides, was detected through phytochemical screening in high concentrations (Mandal et al. 2013). Arjun bark, which is used as a natural dye source in some studies, has been reported by Brahma et al. (2019) as being used as a bio-mordant in their study. They used marigold, *Eucalyptus* leaf, and henna as natural dyes and *Eucalyptus* bark, Arjun bark, and Khair as natural mordants for dyeing cotton fabric. It can be seen that brighter colors generally result from higher quantities of natural mordants. *Eucalyptus* bark exhibits higher color production than Arjuna and Khair among the natural mordants. The yield of color increases as mordant’s concentration is increased. Higher color strength and washing fastness have been noted to be challenging to attain with natural mordants (Brahma et al. 2019).

#### Bahera (*Terminalia bellirica* Roxb.)

One of the first medicinal plants used in Southeast Asia, India, Pakistan, Nepal, Bangladesh, and Sri Lanka, was *Terminalia bellirica* (Gaertn.) Roxb. With about 200 species, the genus *Terminalia* ranks second in size among the Combretaceae family behind *Combretum* (Gupta et al. 2020). Alkaloids, flavones, lignans, tannins, phenols, coumarin, terpenoids, glycosides, and saponins are only a few of the various bioactive secondary metabolites present in *T. bellirica*, which have a wide range of pharmacological activity (Abraham et al. 2014). The article showing that *T. bellirica* can be used as a bio-mordant is presented below. One study used *Rheum emodi* as a source of colored extracted dye and Myrobalan or harda fruit skin (*Terminalia chebula*), anar rind (*Punica granatum*), amla (*Emblica officinalis*), Bahera (*Terminalia bellirica*), and Katha (*Acacia catechu*) as bio-mordants to dye silk fabrics. It is reported that the fastness results of all metallic mordants were very good to washing and sunlight, except tin chloride, and the hues produced by natural mordants, such as amla and Katha, were also viewed as quite positive (Srivastava et al. 2011). In one study, *Carissa carandas* L. was used as a natural dye to dye silk, and *Terminalia bellirica* Roxb. and *Punica granatum* were used as bio-mordants. It is claimed that *Carissa carandas* imparted a brown color range on silk, and as natural mordants, Terminalia bellirica and *Punica granatum* showed increased physical properties with good to very good sunlight and washing fastness properties (Bansal 2014).

#### Banana (*Musa paradisiaca*)

The herbaceous plant known as the banana (*Musa paradisiaca*) is a member of the Musaceae family. It is widely accepted that it came from a tropical part of southern Asia. Bananas also produce a significant amount of biomass as waste in the form of pseudostems, leaves, suckers, etc. Apigenin glycosides, myricetin glycoside, myricetin-3-O-rutinoside, naringenin glycosides, kaempferol-3-O-rutinoside, quercetin-3-O-rutinoside, dopamine, and N-acetylserotonin were the main substances identified in the banana sap (Pothavorn et al. 2010). Some papers in which different parts of the banana are used as bio-mordant are given as follows. It is reported that different shades were obtained due to varying concentrations of dye, and the simultaneous mordanting method gave very good to excellent results for dyed wool samples (Ansari et al. 2018). As tannin, a polyphenolic substance found in banana pseudo stem sap, has great characteristics for the fixing of the pigment molecules. Strong hydrogen bonds provide a cross-link, which improves the dye’s stability and results in an excellent fastness property. It is stated that using the two mordants, banana stem extract produces a color ranging from light vanilla cream to light yellow, and silk fabrics dyed with banana stem extract came out a similar color. Tannic acid was used to dye the fabric, yielding a pale yellow color, and all the dyed samples had good rubbing and washing fastness with no color change (Mariamma and Jose 2018). Mathur and Gupta (2003) obtained concentrated aqueous natural mordants by extraction of banana flower petaloid in a study they conducted. The turmeric-dyed (*Curcuma longa*) Bharat Merino sheep wool yarn was then treated separately with natural mordant and chromium metal salt. They reported that the color fastness and *K/S* values of dyeings made using natural mordants and chromium salt were similar. In a different study, in which *Senegalia catechu* flowers were used as a natural dye for dyeing wool, it was shown that banana pseudostem sap could be used as a bio-mordant (Ansari et al. 2018). In another study, Ansari and Iqbal (2021) used *Butea monosperma* and *Tagetes erecta* flower extracts as natural dyes, banana pseudostem sap, and *Punica granatum* rinds as bio-mordants to investigate the antibacterial activity of selected herbal products used in fabric dyeing. In another study, Diarsa and Gupte (2022) reported that color fastness and dye uptake improved when they used banana pseudostem sap as a natural mordant and marigold (*Tagetes erecta*) as a natural dye. It is stated that the simultaneous mordanting process produced cotton and silk fabrics with the highest color strength, brightest yellow tones, and lowest *L* value. In one study, Phromphen aimed to optimize the dyeing process of marigold flower dye by using banana peel extraction as a bio-mordant in cotton fabrics. It is stated that the washing and light fastness of the cotton fabrics that had been mordanted had both improved (Phromphen 2023).

#### Black cherry stem (*Prunus serotina*)

**Fig. 2:**
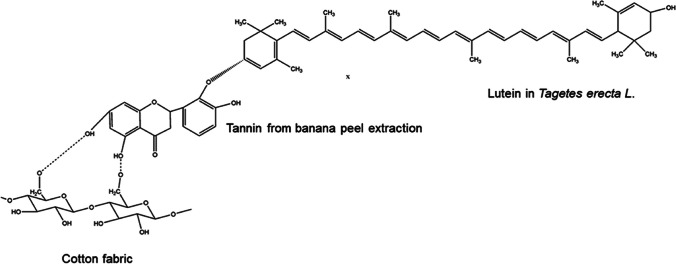
Complex structure formed between bio-mordant, cotton, and dye (this figure was adapted from Thakker and Sun 2022)

Thakker and Sun (2022) reported that hops can be used as a natural dye in dyeing cotton fabric and as natural mordant agents in oak bark, mugwort herb (*Artemisia vulgaris* L*.*), rue herb (*Ruta graveolens*), and black cherry stem (*Prunus serotina*). While the dyed samples had very good rubbing and washing fastnesses, moderately light fastness values were obtained. Bio-mordant, cotton, and dye have a complex structure, shown in Fig. 2.

#### Camphor (*Cinnamomum camphora*)

Camphor is a substance with a strong odor and pungent taste, obtained from camphor laurel (*Cinnamomum camphora*) and other related trees of the laurel family. Topical analgesic, antiseptic, antispasmodic, antipruritic, anti-inflammatory, anti-infective, anti-redness, contraceptive, mild expectorant, nasal decongestant, cough suppressant, etc. Camphor has many pharmaceutical applications. *Cinnamomum camphora* leaf contains camphor as the main ingredient along with cineol, linalool, eugenol, limonene, safrole, α-pinene, β-pinene, β-myrecene, α-humulene, p-cymene, nerolidol, borneol, camphene, and some other ingredients (Hamidpour et al. 2013). Zhou et al. (2020) dyed woolen fabrics with a bio-mordant agent derived from camphor green leaves (chlorophyll extract) and a natural dye derived from tannin-rich waste, Chinese tallow/*Sapium sebiferum* L. It is stated that the use of two metal mordants (ferrous sulfate and alum) and one bio-mordant (chlorophyll extract) enhanced and widened the shade palette, ranging from dark black (ferrous sulfate) to dark yellow (chlorophyll extract) shades.

#### *Chaenomeles speciosa* fruit

In China, the *Chaenomeles speciosa* (CSP) and *Chaenomeles sinensis* (CSS) species are commonly farmed, and their fruits are always plucked just as they are starting to ripen. In China, CSP and CSS are well-known and can be used to make a variety of popular canned foods, preserves, fruit wines, fruit vinegars, juices, and other products. The active components of CSP and CSS have been identified as flavonoids (such as quercetin, luteolin, catechin, epicatechin, and procyanidin B1 and B2), triterpenes (such as oleanolic acid and ursolic acid), phenolics (such as chlorogenic acid), carbohydrates, amino acids, proteins, and tannins (Miao et al. 2016). Yan et al. (2021) stated that dye extracts obtained from dried *Buddleja officinalis* flowers can be used in dyeing hemp fabric with 2 different metals (alum) and ferrous sulfate and 3 different natural mordants (*Chaenomeles speciosa* fruit, gum rosin, and plant ash). Natural mordants have similar effects to metallic ones on fastness after washing, and acid perspiration, and dyed samples.

#### Chestnuts (*Castanea sativa *Mill*.*)

Chestnut (*Castanea sativa* Mill.) is a versatile plant that is grown for lumber, nuts, and tannin and makes a positive contribution to the forestry environment. It is primarily found in the Northern Hemisphere, in Southern Europe, from Turkey to the Atlantic Islands, in Asia, primarily in China, Korea, and Japan, and in the USA (Pereira-Lorenzo et al. 2006). Chestnut wood, bark, and flesh tannin extracts’ chemical makeup has been identified, and they all fall within the category of hydrolyzable tannins (Vázquez et al. 2008). Grifoni et al. (2014) demonstrated that using the tannin found in chestnut as a bio-mordant, vegetable fibers (cotton and jute) can be dyed with various natural dyestuff sources (*Helichrysum italicum Roth* (Roth), *Rubia peregrina* L*.* (wild madder), *Daphne gnidium* L*.* (daphne), *Lavandula stoechas* L*.* (wild lavender), and *Cynara scolymus* L. (artichoke)). It is stated that flax samples dyed with *Helichrysum* extract showed an improvement in UV protection properties when mordanted with potassium alum instead of tannins; chestnut tannins provided a slight support in phenol uptake for *Lavandula* and *Helichrysum* plant dyes with respect to potassium alum, but without an improvement of the fabrics UV protection properties.

#### Chinese tallow (*Triadica sebifera* L.)

**Fig. 3:**
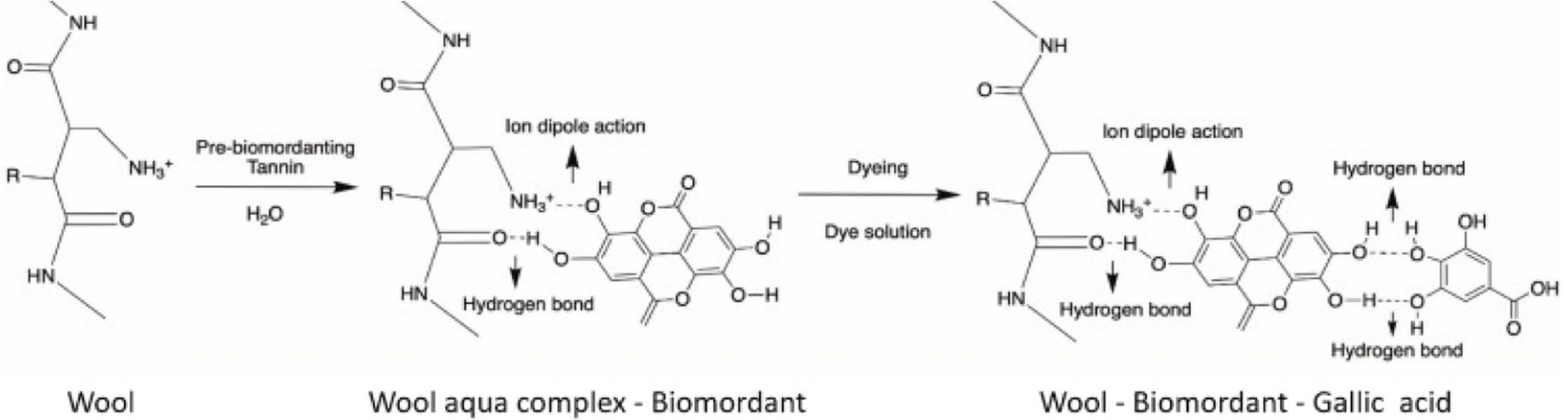
Complex structure formed between wool, bio-mordant, and natural dye (this figure was adapted from Zhang et al. 2021)

Due to a unique combination of rapid growth and excellent stress tolerance, Chinese tallow is the most pervasive non-native tree species in southern US woods and aggressively displaces native tree species (Gan et al. 2009). The large number of flavonoids and phenolics (gallic acid, quercetin, kaempferol, etc.) found in bio-mordants like Chinese tallow displays strong antioxidant activity and is consistent with the relative concentration of the majority of known phenolic components. Zhang et al. (2021) used the seed pods of *Eriobotrya japonica* L. as a natural dye source and the flora leaves of Chinese tallow (tannins), *Folium artemisiae argyi* (eupatilin), and *Cinnamomum camphora* (chlorophyll) as bio-mordants for the coloring of the woolen fabric and for the bio-functional finish. The complex structure formed between wool, bio-mordant, and natural dye is presented in Fig. 3. It is claimed that due to the high contact between fiber and dye molecules activated by functional groups (such as phenolic groups), fabric mordanted with tannin-rich bio-mordant had the best color strength and excellent functional behavior.

#### Chinese tallow (*Sapium sebiferum* L.)

Originating in China, the Chinese tallow tree (*Sapium sebiferum* L.) (Euphorbiaceae) today mostly thrives in subtropical areas of the world and is frequently grown as an ornamental plant. Gallic acid, kaempferol, quercetin, sitosterol glycoside, astragalin, 6-O-galloyl-d-glucose, methyl gallate, and methyl-3,4,5-trihydroxybenzoate have all been found in the leaves of *Sapium sebiferum* (Fu et al. 2013). In one study, this herb was used as a bio-mordant. In this study, *Gingko biloba* L. leaf extract was used as a natural dye, and *Sapium sebiferum* L. and *Cinnamomum camphora* extracts were used as bio-mordants for dyeing wool fabrics. While metal salts showed adverse effects on the antibacterial performance of dyed wool fabric, most of the bio-mordants showed improved antibacterial performance. With metals and bio-mordants, the natural dye made from *G. biloba* L. waste leaves created brown colors in a range of shades and tones. The produced colors included shades of reddish-brown, dark brown, and light brown (Zhou et al. 2022).

#### Chlorophyll

Chlorophyll (a) is a plant pigment that gives plants their green color. Research has shown that using chlorophyll (a) increases wool’s ability to absorb natural colors (Guesmi et al. 2013). Various studies showing that chlorophyll (a) is used as a natural mordant in dyeing processes are listed. In one study, dyes were extracted from *Opuntia ficus-indica* by ultrasound, and the dyeing of wool fabric was investigated using betanin as the natural dye and chlorophyll-a as a bio-mordant. The results show an increase in color strength values with increasing bio-mordant concentrations, and mordanted samples also have good fastness properties (Guesmi et al. 2013). Gong et al. (2020) investigated the possibility of dyeing wool with natural dye obtained from middle-aged or mature leaves of *Cinnamomum camphora*. They reported that the application of different bio-mordants, such as gallnut, pomegranate peel, Arjun bark, chlorophyll extract, and citric acid, produced a range of environmentally friendly shades with a highly diverse color gamut and high color fastness. It is stated that in general, the color and fastness findings of pre-biomordanted wool fibers with *P. granatum*, citric acid, and chlorophyll extract were equivalent to those of metal-treated samples.

#### Coffee

**Fig. 4:**
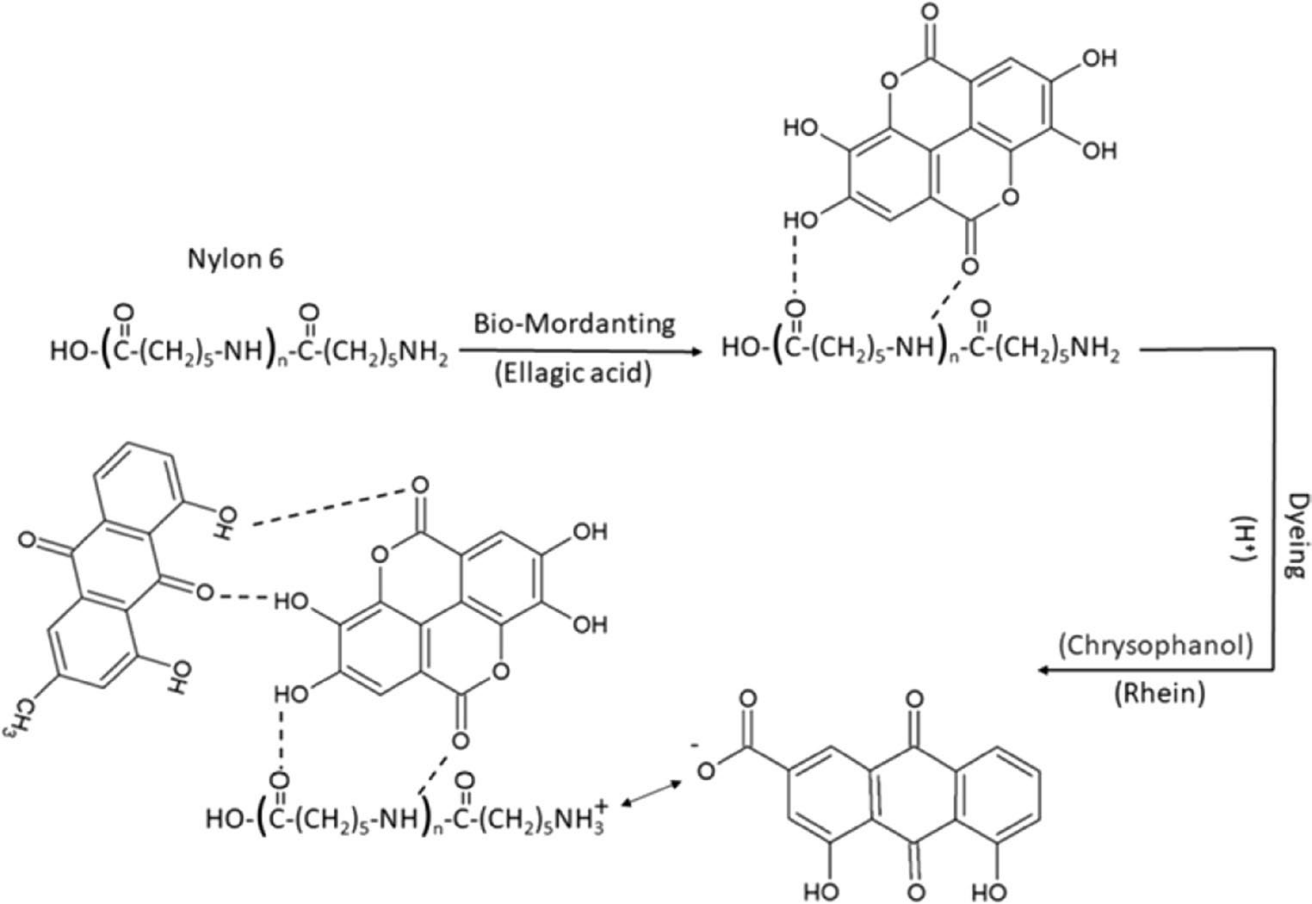
Complex structure formed between nylon 6, bio-mordant, and natural dye (this figure was adapted from Shahmoradi Ghaheh et al. 2023)

According to Shahmoradi Ghaheh et al. (2023), nylon 6 cloth was dyed a brown color using rhubarb flower pieces. Four natural mordants, including walnut husks, pistachio hulls, pine cones, and green coffee, were used to obtain satisfactory color fastness. The strongest color was obtained by 10% of walnut husk; however, several natural mordants all produced the same color (brown). The remarkable color fastness of samples that have been bio-mordanted makes them a useful alternative to metal mordants. The complex structure formed between nylon 6, bio-mordant, and natural dye is presented in Fig. 4.

#### Cypress leaf (*Cupressus sempervirens* L.)

Twelve species of the *Cupressus* genus (Cupressaceae) are found at high altitudes in North America, the Mediterranean Basin, and subtropical Asia. Due to its therapeutic and pharmacological benefits, *C. sempervirens* is frequently used as a cosmetic ingredient in soap and fragrance, including its essential oil, which is extracted from the shoots (Selim et al. 2014). The prominent structures in the chemical composition of *Cupressus* (Cupressaceae) cones, leaves, and seeds are α-pinene, Δ^3^-carene, β-mircene, limonene, and α-terpinolene (Tumen et al. 2010). Kilinc et al. (2015) suggested that this plant can be used as a bio-mordant. In their study, they used the extract obtained from *Chamaecyparis lawsoniana* cones as a natural dye to dye wool fabrics and the extracts obtained from *Cupressus sempervirens*, lemon peel, and larch cones as mordant materials. It is stated that mordanting with larch, lemon peel, and cypress generated better *K/S* values, and the lower mordant concentrations gave, in general, better color change and lower staining values.

#### Date (*Phoenix dactylifera* L.) pits

In dry and semiarid areas of the world, including North Africa, the Arabian Peninsula, and South Asian nations, date palm is widely grown (Shi et al. 2014). In numerous studies, the chemical makeup of date pits has been determined (Ali-Mohamed and Khamis 2004). Other organic molecules, including proteins, alkaloids, steroids, vitamins, phenols, triterpenes, and other groups of chemicals, were discovered through phytochemical and chromatographic screening (Alshowiman 1990). In one of the studies using date seeds, Guesmi and Hamadi (2018) reported that cotton can be dyed with the same extract using the bio-metals found in the date pits (Guesmi and Hamadi 2018). In another study, Baseri (2022a) suggested that wool yarns can be dyed with high fastnesses by using date seeds as an environmentally friendly mordant and Zenian as a dye. In one study, Ltaief et al. (2023) stated that to dye cotton, *Citrullus colocynthis* leaves were used as a dye source, biomordanting with pomegranate peel and date palm pits. It is expressed that pre-biomordanting with pomegranate peel and date palm pits and polyethyleneimine, a co-polymer of dimethyl diallyl ammonium chloride and diallylamin, considerably improved the color strength and exhaustion outcomes when applied to cotton fabric.

#### *Diospyros mollis* L.

The genus *Diospyros* L. (Ebenaceae), distributed in regions such as India, Thailand, Japan, Nigeria, South Africa, and the Philippines, is characterized by its ability to produce triterpenes from the lupine family. It has been reported that there are chemical structures such as luperol, a-amyrine, b-cytostreol, diospyrol, 1,8-dihyroxynapthalene, 8-dihyroxy-2-acetyl-3-methyl, and napthalene in the fruits of *Diospyros mollis* Griff (Maridass 2008). Bunfueang et al. (2019) stated that people in the north of Thailand use the extracted aqueous liquid from the fruit of *Diospyros mollis* as a natural mordant agent for the dyeing of cotton with natural dyes, and the dyed cotton exhibits good wash and light fastness properties.

#### Elaichi (*Elettaria cardamomum* L.)

Small cardamom, green cardamom, and genuine cardamom are all names for the *Elettaria cardamomum* (L.) plant, which is produced in Tanzania, India, Guatemala, Sri Lanka, and Nepal (Garg et al. 2016). According to reports, different polyphenolic components and antioxidant properties are present in cardamom extract from different spices (Shan et al. 2005; Naveed et al. 2013). Adeel et al. (2022c) described a sustainable, green dyeing procedure for silk that included neem, elaichi, turmeric, and zeera as bio-mordants and Esfand (*Peganum harmala*) as a natural dye. The color coordinates are said to show that most of the samples have brighter, redder, and yellower hues; however, the best fabric dyed after ultrasound exposure for up to 30 min has produced brighter shades with a yellowish red tone, and similarly, the acidic extract has produced a brighter shade with a redder and more yellowish tone, while the methanolic extract has produced a brighter tint with a reddish-yellow hue.

#### *Entada spiralis* Ridl

Malaysia is home to the woody climber *Entada spiralis* Ridl (Leguminoceae), whose stem bark has historically been used as shampoo, soap, and a remedy for syphilis and insect bites. Also, in many reports, *Entada* sp. has been shown to have antimicrobial and antifungal activity against various fungal pathogens (Aboaba et al. 2006). In one study, Chairat et al. (2007) investigated the dyeing effect of cotton and silk yarn with an extract obtained from dried fruit peels of mangosteen (*Garcinia mangostana* L.) and a natural mordant solution obtained from the stems of *Entada spiralis* Ridl. It is claimed that pre-mordanting with the natural *Entada spiralis* mordant solution increased the *K/S* value of the dyed cotton compared to pre-mordanting with conventional mordants.

#### Eucalyptus

In their study, Anandhan and Prabaharan (2018) used the extract obtained from the pomegranate peel as a dye to dye cotton fabric, and the extract obtained from the *Eucalyptus* bark as a natural mordant and reported that the obtained dyeing results could be compared with the dyeings made with metal mordants (such as aluminum sulfate, copper sulfate, and ferrous sulfate). Dutta et al. (2021) proposed that onion peel extract can be used as a natural dye and *Eucalyptus* and myrobalan can be used as bio-mordants to dye cotton-knitted fabrics in another study. It is stated that synthetic mordants have good wash fastness, although natural mordants exhibit results that are almost identical. A greater *K/S* value was reportedly obtained for natural mordant in together mordanting compared to synthetic mordant; on the other hand, it was claimed that mixing natural and synthetic mordant produced the best results in together mordanting.

#### *Eurya acuminata*

*Eurya acuminata* is a tall evergreen shrub with 5–7.6 cm long, narrowly oblongelliptic, serrulate, attenuate leaves. It is found widely in Japan, China, India, Sri Lanka, Thailand, Vietnam, Taiwan, and in the hilly forests of Bangladesh. In the chemical analysis of different *Eurya* species, many chemicals were found, including anthrocyanins, ellagic acid, caffeine, flavone, flavonols, β-D-glucopyranoside, euryanoside, and chrysoeriol (Faisal et al. 2016). Vankar et al. (2008b) reported that environmentally friendly dyeing can be done by using *Rubia cordifolia* as a natural dye and *Eurya acuminata* as a bio-mordant to dye cotton fabric. According to the claim, using a bio-mordant not only improves the fastness qualities but also provides useful colorimetric information on dyeing, and even the fastness properties in this instance produce positive outcomes.

#### *Garcinia indica*

*Garcinia indica* is a member of the genus *Garcinia* and the family Clusiaceae. It is indigenous to India and can be found growing there, as well as in forests, along rivers, in wastelands, and in the western ghats of southern India. Mangostin, normangostin, betamangostin, gamma-mangostin, and iso-mangostin are the primary xanthone derivatives found in the fruit hull (Hegde et al. 2011). Hegde et al. (2011) reported that to dye cotton fabrics, *Punica granatum*, *Rubia cordifolia*, *Terminalia chebula*, *Rheum emodi*, turmeric (*Curcuma longa*) root, and annatto (*Bixa orellana*) seed extracts can be dyed using natural dyes and *Garcinia indica* fruit shell as a bio-friendly mordant. It is stated that *Garcinia indica* fruit extract, as a natural mordant dyed with turmeric and applied to organic woven cotton, exhibits fair staining and good washing resistance. In another study, Hegde and Goutham (2015) stated that to dye silk fabrics, turmeric (*Curcuma longa*) root and annatto seed extracts can be dyed using natural dyes and *Garcinia indica* fruit shell as an eco-friendly mordant.

#### Golden (*Cassia fistula*) shower tree bark

The semi-wild Indian labernum *Cassia fistula* Linn., commonly known as the golden shower, has been widely dispersed in a number of nations, including Brazil, Mexico, the West Indies, India, South Africa, Mexico, and China. It is well recognized that the plant organs of *C. fistula* constitute a significant source of secondary metabolites, particularly phenolic chemicals. It has been reported that there are chemical structures such as oxyanthraquinone and dihydroxyanthraquinone, especially in the bark of the tree (Bahorun et al. 2005). Bitter gourd leaf extract can be used as dyes, and turmeric (curcumin), henna (lawson), golden shower peel (tannin), onion peel (quercetin), and pomegranate peel (tannin) can be used as bio-mordants to dye cotton with sufficient color fastness, according to Batool et al. (2022). According to pre-bio-mordanting experiment findings, applying extracts of golden shower bark, pomegranate peels, turmeric, henna, and onion peel before dying has produced a darker hue. This is a result of extra H-bonding between the flavonoid (colorant), cellulosic cotton, and the (-OH) of the bio-mordant.

#### Gooseberry (*Ribes uva-crispa*)

Gooseberry (*Ribes uva-crispa* L.) has been evaluated as a potential source of bioactive compounds with outstanding antioxidant activity due to its phenolic, flavonoid, and anthocyanin contents (Orsavová et al. 2019). In one study, the dyeing potential of waste sawdust (*Pterocarpus indicus* Willd.) was investigated, and an ultrasound-assisted extraction method was applied to extract the natural dye from this sawdust. The resulting extract was used in dyeing cotton and silk fabrics using various metallic and natural mordants such as alum, stannous chloride, copper sulfate, gall nut (*Quercus infectoria*), pomegranate peel (*Punica granatum* L.), and gooseberry (*Ribes uva-crispa* L.), and the process was optimized. In addition, the effect of different pretreatments, such as chitosan and myrobalan, on fabric dyeing was also investigated. It is stated that the preparation of naturally colored cotton and silk fabrics with chitosan and myrobalan is said to considerably improve their color strength, color fastness, and UV protection qualities, and also that the positive a* and b* readings of all dyed materials indicate that they are all in the reddish to yellowish color range (Kandasamy et al. 2021).

#### Guava (*Psidium guajava *L*.*)

The guava tree (*Psidium guajava* L.), like most fruit tree species, exhibits various phenological stages over the course of its vegetative life in response to environmental factors (Salazar et al. 2006). The guava (*Psidium guajava* L.) plant’s green leaves are a reliable source of phenolic substances like tannins. The presence of gallic acids, catechin, ellagic acid, and their derivatives has been mentioned. Additionally, it has coloring molecules called myricetin-3-O-β-D-glucoside. In a study, it was investigated whether jute-cotton combined fabrics can be used as bio-mordants for extracts from banana (*Musa paradaisica* L.) peel and guava (*Psidium guajava*) leaves by using natural dyes extracted from onion skin. In line with the results obtained, it has been suggested that banana peel and guava leaves, which are used as bio-mordants, can be an alternative to metallic mordants. It is stated that extracts from guava leaves produce better color strength outcomes (Islam et al. 2022a, b).

#### Harmal (*Peganum harmala *L*.*)

Harmal (*Peganum harmala* L., family Zygophyllaceae) is a perennial, glabrous plant that is native to the eastern Mediterranean region and grows naturally in semi-arid climates, steppe areas, and sandy soils. Studies on the chemical structure of the extracts reveal that the important components of the plant are quinazoline alkaloids and beta-carboline (Moloudizargari et al. 2013). An example of its application in the textile industry: Azeem et al. (2019b) used logwood extract as a natural dye and zeera (*Cuminum cyminum*), harmal (*Peganum harmala*), and turmeric (*Curcuma longa*) extracts as bio-mordants to dye wool fabrics with natural dyes with the help of ultrasonic energy. It is stated that fabrics dyed with various bio-mordants are much brighter, much redder, and much more yellow.

#### Henna (*Lawsonia inermis *L*.*)

Henna, also known as *Lawsonia inermis* L. (sometimes known as *Lawsonia alba*), is the only species in the genus and a member of the Lythraceae family. From various plant sections, around 70 phenolic chemicals have been identified. Lawsone, a coloring ingredient in naphthaquinones, has been connected to a number of medicinal actions. The strong scent of the essential oil extracted from the flowers is mostly due to the terpene ionone. In addition to various volatile terpenes, the plant has also yielded some non-volatile terpenoids, one sterol, two alkaloids, and two derivatives of the dioxin (Semwal et al. 2014). Henna has been used as both a dye and a bio-mordant in natural dyeing. Some research on their use as a bio-mordant is presented below. Adeel et al. (2019b) used microwave energy to dye wool fabrics with cochineal-based natural dye (carminic acid) and claimed that excellent fastness colors could be obtained by using henna and pomegranate as bio-mordants. It is reported that, comparing the application of bio-mordants to chemical mordants, the color has improved overall and has outstanding qualities. In another study, Batool et al. (2019) used black carrot (*Daucus carota* L.) to dye cotton and chemical (tannic acid and iron sulfate) and bio-mordants (turmeric (*Curcuma longa* L.) and henna (*Lawsonia inermis* L.) to increase color strength. Cotton fabrics that had been dyed showed the best color fastness characteristics in terms of exposure to light, washing, and dry and wet rubbing. In another study, Zuber et al. (2020) suggested that bio-mordants could be used to make the natural coloring process more environmentally friendly. They used neem (*Azadirachta indica*) bark extract as a natural dye by microwave energy in dyeing silk fabrics, as well as turmeric (*Curcuma longa*), henna (*Lawsonia inermis*), pomegranate (*Punica granatum*), and *Acacia* (*Acacia nilotica*) as bio-mordants in their work. It is reported that when used before dying the irradiated silk with colorant extracted from neem bark after microwave treatment for 2 min, lawsone extracted from henna, curcumin extracted from turmeric, quercetin from *Acacia*, and tannin extracted from pomegranate rind have excellent color strength. Adeel et al. (2020b) used yellow dyes obtained from cinnamon bark to dye silk, Al and Fe salts as sustainable chemical mordants, and *Acacia*, henna, rose, pomegranate, and turmeric extracts as bio-mordants and emphasized that the results could be obtained at sufficient fastness values. Amin et al. (2020) also used henna as a bio-mordant in their studies. They used henna leaves, *Acacia* bark, turmeric, and pomegranate bark as bio-mordants for green and sustainable processes from cochineal insects extracted with the help of microwaves in the coloring of silk fabrics and obtained high fastnesses. In the study, where licorice root (*Glycyrrhiza glabra* L.) was used as a natural dye source, cotton fabric was dyed with the effect of ultrasonic waves, while *Acacia*, henna, turmeric, and pomegranate extracts were used as bio-mordants. It is stated that fastness characteristics after bio-mordant application revealed that functional potent molecules interacted with the functional moiety of the colorant and cotton fabric (-OH) so tightly that the shade developed resists fading to a significant degree when exposed to heat, light, crocking, dry cleaning, and perspiration agents (Adeel et al. 2022i). Rasool et al. (2023) also reported that henna can be used as a bio-mordant; in their study, they used the extracts of bougainvillea (*Bougainvillea glabra*) flowers as dyes and henna and turmeric as bio-mordants in the dyeing of cotton and silk.

#### *Ixora javanica* (Rubiaceae)

The lovely red-blooming plant *Ixora javanica*, which belongs to the Rubiaceae family, is noted for having a lot of flavonoid chemicals in it, notably in its flowers. Studies on *I. javanica*’s phytochemistry revealed the presence of flavonoids such as quercetin, formononetin, and anthocyanin (Dontha et al. 2015; Vishwanadham et al. 2016). Indrianingsih et al. (2021) used *G. mangostana* extract as a dye source and *Ixora javanica* leaves as a natural mordant to color cotton fabrics and reported that the dyed fabrics showed antibacterial properties, and colors in orange, brown, and green tones were obtained.

#### *Iris germanica* (I. versicolor)

*Iris germanica* (*I. versicolor*) is a member of the Iridaceae family and includes tannin, volatile oil, starch, salicylates, and acrid resin (irisin). It is a blood purifier and helps with skin issues (Mabey 1988). Nine isoflavonoids isolated from *Iris germanica* L. were tested for their anti-inflammatory properties using activated human neutrophils in a spectrophotometric assay (Rahman et al. 2003). In his study, İşmal (2017) used pomace extract as dye and *Iris germanica* (*I. versicolor*), valex (acorn of *Quercus ithaburensis* ssp. macrolepis), pomegranate rind (*Punica granatum* L.), rosemary (*Rosmarinus officinalis*), and thuja (*Thuja orientalis*) as bio-mordants to dye woolen fabrics with a clean and sustainable production approach. It is stated that, compared to the control sample, large quantities of thuja, iris, and rosemary generally seemed to improve darkness.

#### *Justicia carnea *L

*Justicia carnea* L. is a flowering plant commonly found in various parts of Africa. It has been reported that there are chemical structures such as terpenoids, phenols, and flavonoids within the plant (Onyeabo et al. 2017). In one study, Jabar et al. (2022) used *Justicia carnea* L. leaves, *Moringa oleifera* L. bark, *Azadirachta indica* L. bark, and *Vernonia amygdalina* leaves as bio-mordant agents to dye cotton fabric with natural dyes. It has been reported that different colors can be obtained, from yellow to chocolate, depending on the type of mordant applied.

#### Karuvelam (Prosopis juliflora) tree bark

Another herb used as a bio-mordant is Karuvelam bark. Karuvelam is a difficult plant to remove by hand due to its spines and deep roots that suppress the growth of neighboring plants (Devadharshini et al. 2018). Jayalakshmi and Amsamani (2008), and Jayalakshmi (2017) emphasized that annatto, catechu, and eclipta extracts can be used as natural dyes, and Karuvelam, myrobalon, and neem bark can be used as bio-mordants. It has been reported that both good washing and light fastness can be obtained in dyeings made by using bio fixing agents together with natural dyes.

#### Lemon (*Citrus lemon *L*.*)

Lemons and limes are the fruits that have the highest citric acid overall. Citrus fruits naturally contain a high concentration of the weak tricarboxylic acid known as citric acid (Penniston et al. 2008). Citric acid, found in lemon juice or peel, has been used as a bio-mordant in different studies. Sundrarajan et al. (2012) investigated the silk dyeing properties of Parijatak (*Nyctanthes arbor-tristis*) flowers and used pomegranate, tannin, and lemon juice as natural mordants as well as metal mordants. In another study, it was used in the natural dyeing process of wool by combining it with lemon juice, copper sulfate, potassium dichromate, and iron sulfate in different proportions. It has been stated that, depending on the mordant substance, dyeings can be obtained in perfect colors from yellow to green (Singh and Purohit 2012, 2014). Kanchana et al. (2013) used *Clitoria ternatea* (clitoria flowers), *Tagetes erecta* Linn (Marigold) flowers, *Punica granatum* (pomegranate) peel as natural dyes, and lemon as a natural mordant in their research to dye cotton and synthetic fabrics. Maryam et al. (2019) were among the researchers who used lemon juice as a mordant agent. They used *Allium cepa* onion skin to dye cotton fabric and tannic acid, *Aloe vera*, and lemon juice as natural mordants. It has been reported that different color tones can be obtained, from brown to purple, depending on the mordant material and the mordanting method. A series of studies using lemon juice as a mordant can be summarized as follows: alum, acetic acid, CuSO_4_, and lemon juice were used to dye cotton, silk, and polyester yarns using different natural dye sources such as sepals of *Mussaenda* hybrid, fruits of *Carissa carandas*, *Syzygium cumini*, pink leaves of *Cordyline fruticosa*, and red sepals of *Mussaenda erythrophylla*. It has been reported that different color tones can be obtained, from pale yellow to green, depending on the mordant agents (Manicketh and Francis 2020; Manicketh et al. 2021, 2022). Arain et al. (2021) used lemon as a bio-mordant to provide an environmentally friendly dyeing process for polyester fabric with henna. In one study, Tehrani et al. (2023b) stated that pre-, meta-, and post-mordanting techniques were used to dye silk fibers using a dye that was derived from spent coffee grounds (SCGs) with various metallic and natural mordants. Mordants included natural substances like pinecone, tannic acid, and lemon peel, in addition to metal salts like tin chloride and copper sulfate. According to the findings, metal samples displayed stronger color in all methods compared to bio-mordant samples. Additionally, in terms of fastness criteria, the utilized bio-mordant might be a good replacement for metal mordants.

#### Mango (*Mangifera indica* L.) tree bark

*Mangifera indica*, usually referred to as the mango, has long played a significant role in both Ayurvedic and traditional medical practices. The plant’s various chemical components, including its polyphenols, flavonoids, and triterpenoids. Isomangiferin, tannins, and gallic acid derivatives are the main bioactive components of the xanthone glycoside mangiferin (Shah et al. 2010). Jayalakshmi and Amsamani (2007) used manjistha and ratanjot as natural dyes to dye wool yarn, and pomegranate peel and mango peel as bio-mordants. In order to create environmentally friendly hues, Wangatia et al. (2015) investigated the application of mango tree bark mordant to cotton using a pre-mordanting or post-mordanting approach. They used a natural dye made from bitter plants and discovered that post-mordanting with mango produces stronger colors and better color fastness than pre-mordanting. Other researchers using mango peel as a natural mordant are Bhandari et al. (2021). In their work, they used the bark of *Castanopsis indica* as a dye to dye the fabric and *Aloe vera* and mango bark extract as natural mordants. It has been reported that colors in brown tones are obtained depending on the mordant substance. In one study, the effects of bio-mordants on the dyeing qualities of *Euclea divinorum* Hiern (Ebenaceae) dye extract were evaluated using several mordanting techniques on cotton fabric, according to Manyim et al. (2023). For cotton fabric that has been dyed, the bio-mordants increased the color strength from 0.612 to 0.863 and 0.911 for mango and rosemary, respectively.

#### Manjakani *(Quercus infectoria)*

Because it is believed to contain significant amounts of bioactive components such as tannin, gallic acid, syringic acid, ellagic acid, P-sitosterol, amentoflavone, and hexamethyl ether, *Quercus infectoria* has been widely utilized as a medicinal plant since ancient times (Ab Rahman et al. 2015). In another study, myrrh (*Commiphora molmol*) extract was used as a natural dye, and sumac (*Rhus coriaria*) and manjakani (*Quercus infectoria*) were used as bio-mordants to dye wool and silk fabrics. According to the findings, samples dyed with sumac have a stronger hue than those colored with manjakani (Al Alamoudi and Salem 2019).

#### Manjistha (*Rubia cordifolia *L.)

A species of flowering plant belonging to the coffee family, Rubiaceae, is called *Rubia cordifolia*, also referred to as Common Madder or Indian Madder. Manjistha is a key herbal medication used in Indian medicine. Its scientific name is *Rubia cordifolia* L. (family Rubiaceae). The plant contains chemical structures such as anthraquinone, munjistin, purpurin, pseudopurpurin, and triterpenoids (Verma et al. 2016). In one study, manjistha was used as a natural mordant along with myrobalan, pomegranate rind, and *Rhizophora mucronata* fruits. It is stated that the fruits of *Rhizophora mucronata* function as a better mordant than the other natural mordants when they are utilized, and the fact that natural mordants outperformed metal salts like SnCl_2_ in terms of color strength is particularly noteworthy (Praveena et al. 2014).

#### *Mimosa pudica *L*.*

A subshrub with delicate soft gray-green leaves that fold and droop at night or when touched and chilled, *Mimosa pudica* L. (Mimosaceae) is a prostrate or semi-erect species native to tropical America, Australia, and India. Alkaloids, a non-protein amino acid (mimosine), flavonoids (C-glycosides), sterols, terpenoids, tannins, and fatty acids have all been identified in phytochemical research on *M. pudica* (Ahmad et al. 2012a). This plant was also used as a bio-mordant. In dyeing wool and nylon fabrics, date palm fiber fibrillium was used as a natural dye and *Mimosa pudica*, and tannic acid as bio-mordants. It is reported that the dyeability and fastness qualities are improved when mordants are combined with extracts (Baaka et al. 2023).

#### Moringa (*Moringa oleifera* Lam)

A tree known as *Moringa oleifera* Lam is seen growing profusely in many tropical and subtropical nations. Commercial cultivation of it is practiced in India, Africa, South and Central America, Mexico, and Hawaii, as well as throughout all of Asia and Southeast Asia. In traditional medicine, seeds, leaves, oil, sap, bark, roots, and flowers are frequently employed. According to reports, the leaves contain a variety of antioxidant substances, including ascorbic acid, flavonoids, phenolics, and carotenoids (Stohs and Hartman 2015). In one study, *Moringa* was used as a natural mordant along with turmeric to dye viscose fabric with natural dyes. Depending on the mordant used, it has been stated that color tones can be obtained from light yellow to pale pink (Raza et al. 2018).

#### *Mucuna pruriens *L*.*

*Mucuna pruriens* is a member of the Fabaceae family, also known as the cowage plant. *Mucuna pruriens* leaves are used to treat syphilis, ringworm, scorpion stings, coughs, dog bites, discomfort, pleuritis, and fractured bones. Alkaloids, reducing sugar, anthraquinones, flavonoids, saponins, tannins, cardiac glycosides, phenols, and steroids were found in *Mucuna pruriens* during phytochemical screening (Divya et al. 2017). Jabar et al. (2023a) used *Mucuna pruriens* as a bio-mordant in natural dyeing. They used mango peels as a natural dye and *Mucuna pruriens* L. and *Justicia carnea* L. as bio-mordants for dyeing silk fabrics.

#### Mugwort herb (*Artemisia vulgaris* L.)

With more than 500 species, the *Artemisia* genus is part of the subtribe Artemisiinae of the tribe Anthemidae in the Asteraceae family. These species are found around the planet, but they are most prevalent in regions with moderate climates, such as those in Europe, East Asia, the Americas, North Africa, and Australia. Additionally, they are a good source of phenolic acids, coumarins, and flavonoids (Ekiert et al. 2020). Thakker and Sun (2022) reported that hops can be used as a natural dye in dyeing cotton fabric and as natural mordant agents in oak bark, mugwort herb (*Artemisia vulgaris* L*.*), rue herb (*Ruta graveolens*), and black cherry stem (*Prunus serotina*). It is stated that very good rubbing and washing fastnesses and moderate light fastness values were achieved for the dyed samples.

#### *Musa basjoo *leaf

*Musa basjoo* is a member of the Musaceae family. It has been found that *Musa basjoo* contains anthraquinone, flavonoids, coumarins, phenols, cyanide, amine, coumarin, and other chemicals that can interact with certain dyes and fabric macromolecules simultaneously. Textiles (wool, silk) have been dyed using the leaves, bark, and fruit rinds of *Musa basjoo* as a mordanting agent. Wool and silk fabrics can be functionally dyed while also achieving ecological dyeing thanks to the tannin in *Musa basjoo* leaves used as a bio-mordant (Zhang et al. 2022a, b).

#### Myricaceae (*Myrica esculenta*)

The evergreen, sun-tolerant tree *Myrica esculenta* (Myricaceae), also referred to as “Katphala,” can reach a height of 12 m. *Myrica esculenta* bark is said to have a wide range of therapeutic benefits. The bark has been shown to contain a variety of phytochemicals, including gallic acid, myricanone, myricanol, and tannins, including epigallocatechin-3-o-gallate (Jain and Jain 2010). Semwal et al. (2012) used metal and natural mordant materials (the extract of the stem bark of *Myrica esculenta*) to dye cotton fabrics and yarns with natural dyes.

#### Myrobalan (*Terminalia chebula* Retz)

**Fig. 5:**
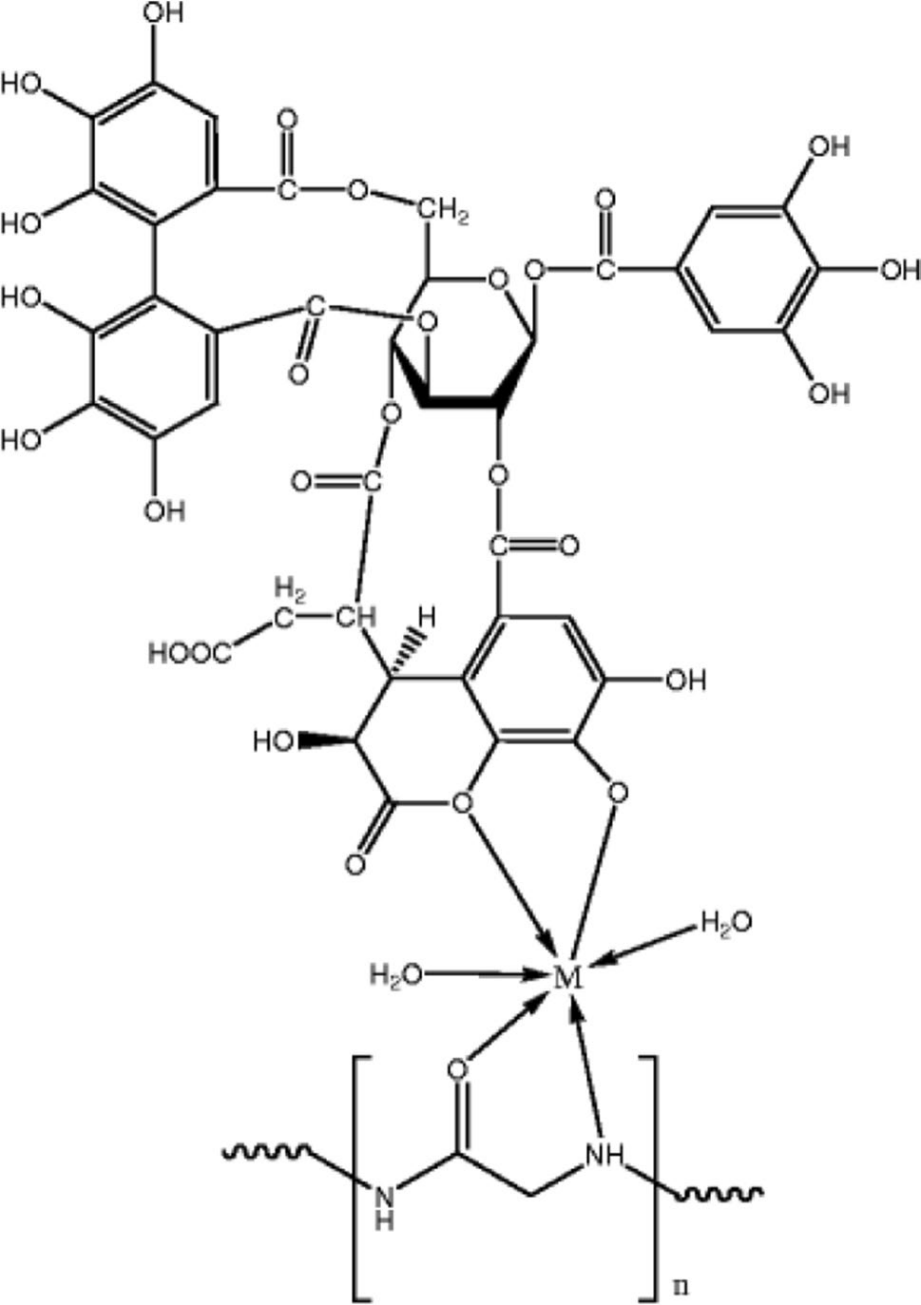
Chebulagic acid, metal ions, and functional groups in wool fiber produce a complex (this figure was adapted from Shabbir et al. 2017; Kasiri and Safapour 2013)

It is among the most significant and popular mordants used in dyeing procedures. It can be used as both a dye and a mordant. Fruits of *Terminalia chebula*, most popularly referred to as “harda,” are used to make myrobalan mordant. It gives textiles a soft yellow tint. The largest significant source of tannin is myrobalan (Khan et al. 2005). There are many studies available for the use of myrobalan as a mordant agent. The basic chemical structure of myrobalan is presented in Fig. 5. In a study, it was emphasized that using a combination of harda and tartaric acid as natural mordants in the coloring of ecru denim fabrics showed a synergistic effect (Deo and Paul 2000). In another study, Teli et al. (2001) used eco-friendly alum and natural mordant harda while dyeing denim fabrics with onion extract. In addition, they reported that alum and harda can be used alone or together. The bleached jute fabric was colored with natural mordants such as babool (*Acacia arabica*) and mirobolan using different natural dyes (Samanta et al. 2009). The silk fabric was colored with the flower of *Cordia sebestena*, while myrobalan (*Terminalia chebula*) was used as a bio-mordant. In another study, bleached cotton fabric was colored with natural dyes, while myrobalan (*Terminalia chebula*) was used as a natural mordant. It has been reported that various colors can be obtained with high color fastnesses by using different mordant combinations (Kumaresan et al. 2011, 2012a, b). Similarly, baked cotton fabric was dyed using *Tecoma stans* flowers, while myrobalan and cow dung were used as natural mordants (Chandra et al. 2012). In another study, flower extracts of *Spathodea campanulata* and *Cordia sebestena* as natural dyes and myrobalan as a natural mordant were used for dyeing cotton samples. It is stated that various colors with good color fastnesses can be obtained by using different mordant combinations (Kumaresan et al. 2013). Chattopadhyay et al. (2013) also used myrobalan as a bio-mordant. In their work, they used manjistha, annatto, ratanjot, and babool as natural dyes, and myrobalan and pomegranate as bio-mordants for dyeing jute fabric.

It is stated that after premordanting with a bio-mordant and chemical mordant mixture, any naturally dyed jute cloth generates exceptionally good UV protection qualities. It was emphasized that while natural dyes like pomegranate and marigold (*Tagetes eracta* Linn.) were used in the coloring of cotton and synthetic fabrics, tannin-based harda could be used as a natural mordant (Tripathi et al. 2015). Stinging nettle fabric was dyed using *Acacia catechu* and madder (*Rubia cardifolia*), while myrobalan was used as a natural mordant. It is stated that the highest UPF was observed in the sample mordanted with myrobalan (Pargai et al. 2016). While silk fabric was dyed with a natural dye extract obtained from red prickly pear fruit (*Opuntia ficus-indica*), myrobalan was used as a natural mordant. It is reported that, compared to natural mordant-dyed fabrics, synthetic mordant-dyed fabrics have stronger color retention (Ganesan and Karthik 2017). In the trials where Indian rhubarb (*Rheum emodi*) was used as a natural dye source to obtain dark and bright tones in wool yarn samples, myrobalan was used as a natural mordant (Khan et al. 2017). Jadav and Gowda (2017) used myrobalan as a natural mordant agent to dye cotton with natural dyes. The mordanting process not only increased the color strength of the natural dye but also resulted in a large number of different color tones, and it was also stated that all mordanted samples showed excellent color strength, moderate to good color fastness values, and strong antioxidant activity. In another study, the UV protection properties of dyed material using pomegranate peel as a natural dye and myrobalan as a natural mordant were investigated (Sinnur et al. 2018). Ashrafi et al. (2018) used myrobalan as a bio-mordant in their research. It is stated that the red color created by myrobalan extract as a bio-mordant had medium fastness capabilities, offering full potential to substitute metal salts in the dyeing process. It has been reported that dyeing fabric with high fastnesses can be obtained by using myrobalan as a bio-mordant in coloring linen fabric with natural dyes (Teli and Pandit 2018). *Parkia speciosa*, *Punica granatum*, *Rubia cordifolia*, *Caesalpinia sappan*, and *Clerodendrum indicum* as natural dyes, and myrobalan as an environmentally friendly mordant, were used for dyeing silk and cotton fabrics. During printing with natural colorants, all fabrics are first treated with myrobalan and then printed with metallic salts. It is reported that silk and cotton dyed in this way show good washing and rubbing properties (Banerjee et al. 2019). Myrobalan was used as a natural mordant for dyeing wool and silk fabrics with natural dyes. It is stated that with and without mordanting, treated fabrics showed appropriate wash, light, and rubbing fastness, and with mordanting, treated silk and wool fabrics demonstrated good to very good UV protection properties (Patil et al. 2020). Singh and Sheikh (2020) also used myrobalan as a bio-mordant. In the study, they obtained sufficient fastness values from the dyes they made using bio-mordant. In one study, myrobalan was used as a natural mordant to dye silk, cotton, and nylon with natural dyes (*Terminalia arjuna* and *Thespesia populnea* trees). It is stated that in contrast to the pale yellow tone in cotton, both dyes produced a vibrant yellow color in silk and nylon materials (Amutha et al. 2020). Rajeswari (2020) also used myrobalan as a natural mordant in his natural dye work. Hosseinnezhad et al. (2020, 2022a) colored wool fibers using madder as natural dyes and yellow myrobalan and black myrobalan as natural mordants. It has been found that the washing, light, and rubbing fastnesses of dyeing on the fibers increase when bio-mordants are used in the dyeing process.

#### Neem (*Azadirachta indica *L*.*)

For more than 2000 years, the Indian subcontinent has recognized *Azadirachta indica*, also known as neem, as one of the most adaptable medicinal plants with a wide range of biological activity. More than 140 chemically diverse and structurally complex active compounds have been identified from various neem components (Subapriya and Nagini 2005). While neem extract has been used as a natural dye source in some studies, Habib et al. (2021) used neem as a bio-mordant in their research. In their study, they used the extract they isolated from natural dye in Arjun bark to dye cotton fabric with natural dye and neem (*Azadirachta indica*), zeera (*Cuminum cyminum*), elaichi (*Elettaria cardamomum*), and harmal (*Peganum harmala*) extracts as bio-mordants. It is stated that the color coordinates show that the bio-mordants have primarily produced much brighter shades with a reddish-yellow hue, but a fabric dyed while applying 10% zeera extract after US treatment using an acidified methanolic extract of Arjun bark has produced much darker shades with a reddish-yellow tone (Habib et al. 2021).

#### Oak (*Quercus*)

In the northern hemisphere, the genus *Quercus* (commonly named oaks) is among the most significant clades of woody angiosperms in terms of species diversity, ecological dominance, and economic importance (Nixon 2006). Bioactive groups such as ellagic acid, gallotannin/ellagitannin, phenolic compounds (flavonoids), tannins, and triterpenoids were detected in the leaves, bark, galls, shells, and acorns of the oak tree (Morales 2021). It is possible to come across the fact that very different parts of the oak tree are used in the process of coloring textile materials with natural dyes. Studies related to these are presented below. Grifoni et al. (2011) investigated the UV protection properties of fabrics such as cotton, linen, hemp, and ramie dyed with natural dyes using a tannin-based natural mordant substance (the galls of *Quercus infectoria*). It is stated that when fabric construction was appropriate, the employment of the tannin-based mordant boosted the UV protection level up to the very high and/or excellent protection categories, even without dyeing. In another study, it was emphasized that cotton fabrics showed high antioxidant and antibacterial properties when the gallnut of the oak tree was used as a natural mordant and the roots of gromwell (*Lithospermum erythrorhizon*) were used as a natural dye source (Hong et al. 2012). In another study, it was reported that dyed fabrics with high color fastness could be obtained by using the extract obtained from the wastes of red pine (*Pinus brutia* Ten.) timber as a natural dye and oak ash as a natural mordant in dyeing things like cotton, linen, wool, silk, tencel, polyamide, and acrylic (Avinc et al. 2013). In a study, the usability of natural dye extracts obtained from *Helichrysum arenarium* and natural mordant obtained from *Quercus aegilops* in dyeing Karabük Eflani cloth, a local weave, was examined. It is reported that all the dyeings were red-nuanced in terms of lightness and darkness, and also in terms of yellowness and blueness, blue nuance was higher in the sample at 5% concentration, and yellow nuance was higher in other samples (Akkaya and Eyupoglu 2016). While investigating the dyeing of wool yarn with *Adhatoda vasica* extract as a natural dye, natural tannin extracts (gallnut (*Quercus infectoria*), pomegranate (*Punica granatum*) bark, and babool bark (*A. nilotica*)) were evaluated comparatively. It is stated that gallnut may be a useful and possible replacement for metal mordants because it guarantees the most comparable and improved dyeing results among the bio-mordanted samples when combined with *A. vasica* (Rather et al. 2016). In another study in which gallnut (*Quercus infectoria*) was used as a bio-mordant, it was reported that dyeing wool yarns with anthraquinone obtained from Indian madder (*Rubia cordifolia*) was obtained with sufficient color fastness (Yusuf et al. 2017a). Nakpathom et al. (2018, 2019) performed natural dyeings on cotton and polyester fabric using purple corncob and annatto extract as natural dyes and gallnut (*Quercus infectoria* Olivier) as a mordant. It is stated that when potassium aluminum sulfate, ferrous sulfate, and gallnut mordants were used throughout the dyeing process, the color strength decreased but the color fastness qualities remained practically the same. In the other study, babul bark (*Acacia nilotica* L.) was used as a natural dye, and gallnut (*Quercus infectoria*) as a bio-mordant was used to dye cotton fabric. It is stated that the high UPF value obtained by the absorption of UV light by gallotannins in thuja and Babylon bark shows good UV protection properties in dyed cotton fabrics (Dhanania et al. 2021). In order to obtain a green, clean, environmentally friendly yarn dyeing, two natural dyes, Iranian madder and weld, and tannin-rich oak extract as a bio-mordant were used for carpet yarn dyeing (Hosseinnezhad et al. 2021a). In one of the studies, the usability of the natural dye obtained from the leaves of the olive tree in cotton fabrics woven with handmade threads was investigated, while *Quercus aegilops* L. (Valonia oak) was also used as a natural mordant along with citric acid (Özomay et al. 2022b). While examining the use of natural dye obtained from the *Sambucus ebulus* L. plant in hemp fabric dyeing, gallnut extract (*Quercus aegilops* L.) was used as a natural mordant. It is stated that the best dyeing characteristics were obtained using a 15% concentration of *Quercus aegilops* natural mordant, which was used to dye hemp fabric with *Sambucus* with the least amount of color difference and the greatest amount of color efficiency (Özomay and Akalın 2022a; Özomay 2023).

#### Oleaster or Russian olive (*Elaeagnus angustifolia* L.)

The family Elaeagnaceae, widely known as the Russian olive and wild olive, includes oleaster, scientifically known as *Elaeagnus angustifolia* L. The Semnan region of Iran is home to a large population of this species, which is found around the world but primarily in tropical areas. This tree typically lives a long time and begins to produce fruit after 5 years. This tree produces fruit that is highly nutritious and abundant in protein, vitamins, minerals, and tannins. According to Hamidpour et al. (2017), *E. angustifolia* L., which accounts for roughly 78 mg per 100 g of the fruit’s dry weight, is the fruit’s most significant phenolic group. In one study, the oleaster, or Russian olive, and its parts, which are one of the sources rich in tannin, were used as natural mordants. It is stated that with the different bio-mordants used, different color tones of red can be obtained with sufficient fastness and color strength (Hosseinnezhad et al. 2023b).

#### Onion (*Allium cepa *L*.*) skin

Quercetin and its derivatives have been shown to be abundant in onion skin (Świeca et al. 2013). In many studies, onion peel has been evaluated as a natural dye source. However, it has been reported that onion peel can be used as a natural mordant. In a study, Amutha and Annapoorani (2019) reported that banana leaves can be used as a natural dye to dye cotton, and onion (*Allium cepa*) and pomegranate (*Punica granatum*) rinds can be used as natural mordants. Acceptable color fastnesses have been reported to be achieved. In another study, onion skin was similarly used as a bio-mordant. It has been reported that cocklebur (*Xanthium strumarium* L.) leaves can be used as natural dyes, and onion peel, *Acacia*, and *Eucalyptus* can be used as bio-mordants in dyeing cotton fabric. It is stated that the usage of bio-mordants revealed notable differences in color intensity, color shade, and fastness characteristics depending on the scope of interaction generated between functional mordant molecules, cotton, and dye molecules (Hussaan et al. 2023).

#### Orange (*Citrus sinensis *L*.*) peel

Three different forms of flavonoids are found in citrus fruit: flavanones, flavones, and flavonols. Hesperidine, narirutin, naringin, and eriocitrin are the principal flavonoids present in *Citrus* species (Hegazy and Ibrahium 2012). In one study, extracts of harda, amla, pomegranate, and orange were used as natural mordants in an approach to using natural colorants from various plant sources, such as Kalanchoe-pinnata, papaya, peepal, and banyan, for dyeing wool fabric (Rani and Jajpura 2021). In another study, Adeel et al. (2022e) used coral jasmine (*Nyctanthes arbortristis*) as a natural dye for dyeing wool and *Acacia* bark, orange peels, pomegranate peels, and tannic acid as bio-mordants. It has been reported that, depending on the mordant material, green and gray color tones can be obtained with sufficient fastness values.

#### Papaya (*Carica papaya *L*.*)

One of the most important fruit crops grown in tropical and subtropical regions is papaya, *Carica papaya* L., which contains two important active compounds such as chymopapain and papain (Silva et al. 2007). In addition to phenolic compounds such as erulic acid, caffeic acid, and rutin in papaya fruit, lycopene, β-cryptoxanthin, and β-carotene were identified as carotenoids (Rivera‐Pastrana et al. 2010). According to Rosyida et al. (2022), cotton fabrics could be dyed with jackfruit wood extract, papaya fruit extract as a bio-mordant agent, and metallic mordants in one study. It has been reported that, depending on the mordant agents, green, purple, and gray color tones can be obtained with sufficient fastness properties.

#### Peltophorum pterocarpum

*Peltophorum pterocarpum* is a member of the Fabaceae family native to tropical Southeast Asia and is a widely cultivated ornamental tree worldwide. It has been reported that chemical structures such as leucocyanidin and leucocyanidin-3-O-α-D-galactopyranoside are found in the bark of the plant (Jash et al. 2013). In a study, it was emphasized that *Peltophorum pterocarpum* can be used as a bio-mordant for dyeing yarn. It is shown that the presence of betalain as a pigment gives a reddish color (Mukherjee and Kanakarajan 2017).

#### Peppermint (*Mentha piperita *L*.*)

A perennial aromatic herb in the Lamiaceae (Labiatae) family, peppermint or mint (*Mentha piperita* L.) is a natural hybrid of water mint (*Mentha aquatic* L.) and spearmint (*Mentha spicata* L.). Chemical structures such as menthol, isomenthone, menthone, and cineole have been defined in the mint plant (Loolaie et al. 2017). According to Haji et al. (2023), they used dragon’s blood resin extract as a natural dye and 4 different bio-mordants (including peppermint, mugworts (*Artemisia*), *Dorema ammoniacum* gum, and pomegranate rind) as natural dyes to dye nylon fabric with natural dye. It has been reported that the color strength of dyed fabrics mordanted with all mordants increased and also improved color fastness to washing.

#### Pine (*Pinus pinea *L*.*) cone

One of the most widespread species in both Europe and North America is the pine tree, although its production and consumption importance are at their peak in the Mediterranean region (Ayrilmis et al. 2009). Pine cone extracts have been reported to include tannins and other lignin-related substances that may have antibacterial, antiviral, and anticancer effects (Micales et al. 1994). Ghaheh et al. (2022) used pine cone as a bio-mordant in their studies. They used *H. sabdariffa* L. extract as a natural dye and sodium alginate, walnut hull, lemon peel, and pine cone as bio-mordants to dye viscose rayon fabric in their studies. It is stated that the inclusion of walnut, lemon, and pinecone mordants increased the colored fabric’s light fastness rating, which ranges from 5 to 6, as well as the highest wash and light fastness, which was also attained when the post-mordanting procedure was applied.

#### Pine (*Pinus pinea *L*.*) nut shell

The most significant edible wild seed gathered from Mediterranean woodlands is the *Pinus pinea* pine nut. Stone pine, *P. pinea* L., is a valuable species that is also used to produce timber, but it is best known for producing pine kernels, which are highly appreciated on the market for their excellent nutritional value and delicate flavor (Queirós et al. 2020). Pine nut shells have been used as a bio-mordant in some studies. According to Ul Hasan et al. (2022), *Cassia ovata* has been valued as a natural yellow dye source for nylon dyeing with sustainable bio-mordants (turmeric rhizomes, pomegranate peels, *Acacia* bark, and pine nut hull). It is claimed that bio-mordants have provided great color strength in addition to relaxing tints. In another study, coral jasmine flowers were used as a natural dye and pine nuts and walnut shell as bio-mordants in the dyeing of wool fabric in a microwave environment. Walnut shell and pine nut shell used as bio-mordants have been reported to create yellowish-green hues on wool (Adeel et al. 2023b). In another study, *Butea monosperma* plants were used as a natural dye and pine nut hulls and *Acacia* as bio-mordants in the dyeing of silk fabric in a microwave environment (Adeel et al. 2023c). According to Talib et al. (2023) they used black pepper (*Piper nigrum* L.) as a natural dye, walnut shells (*Juglans regia*), pine nut shells (*Pinus pinea* L.), and orange peel (*Citrus sinensis*) as bio-mordants in their work. Adeel et al. (2023e) mentioned in one study that an attempt was made to determine whether *Rheum emodi* (rhubarb) extract might be utilized as a natural dye for wool dyeing in order to replace synthetic dyes. The treatment of wool cloth with microwave (MW) rays was done in order to color it. A magnificent color palette of hues and tones was produced by blending different mordants. To determine the best mordant for each application, a comparative analysis of the effects of various chemical mordants (aluminum salt, iron salt, tannic acid, and cream of tartar) and bio-mordants (pomegranate extract and pine nut hull extract) on the characteristics of dyed wool samples was conducted.

#### Pistachio (*Pistacia vera* L.)

The pistachio (*Pistacia vera* L.) belongs to the Anacardiaceae (Barghchi and Alderson 1989); the pistachio, or *Pistacia vera* L., is a tree native to the arid regions of Central and West Asia and is widely cultivated throughout the Mediterranean region (Tomaino et al. 2010) sometimes known as the cashew family. Pistachio hull is a different source of physiologically active substances because of its high phenolic content and antioxidant activity (Özbek et al. 2020). Pistachio shells used for different purposes were also used as bio-mordant material. Habib et al. (2022) used turmeric as a natural dye and *Acacia*, pomegranate, and pistachio extracts as bio-mordants to dye wool in a microwave environment. It has been reported that samples in dark yellow and pink tones were obtained, depending on the mordant substance. In another study, haar singhar (*Nyctanthes arbor-tristis*) flowers were used as a natural dye and pistachio as a bio-mordant to dye cotton fabric. It is stated that, as a natural mordant of pistachio shell, deep color tones can be obtained on cotton fabrics (Yameen et al. 2022). In one study, bio-mordant included extracts of pine nutshell (*Pinus gerardiana*), walnut shell (*Juglans regia*), and pistachio shell (*Pistacia vera*). It is stated that the pistachio shells used as a bio-mordant produced reddish-yellow colors of excellent quality (Nasreen et al. 2023).

#### Pomegranate (*Punica granatum* L.) rind

The pomegranate, or *Punica granatum* L, is a fruit crop with great agro-climatic adaptability. It is a Punicaceae family member and a deciduous fruit-bearing shrub or small tree. Iranians are the original cultivators of the pomegranate fruit, which is mostly grown in Tunisia, Turkey, Spain, Egypt, Morocco, the USA, China, India, Argentina, Israel, and South Africa. It has a wide range of phenolic compounds, which are sources of natural antioxidants and have drawn the interest of numerous researchers and medical professionals. These substances can be further separated into a number of subgroups, including phenolic acids, flavonoids, and tannins, mostly based on the structural components that link benzene rings and the quantity of attached hydroxyl groups (Belemkar and Ramachandran 2015; Singh et al. 2018). Pomegranate peel is one of the vegetable sources used both as a natural dye and as a bio-mordant. Studies on their use as a bio-mordant are presented below. Deshmukh and Patil (2008) used *Carissa carandas* as a natural dye, pomegranate rind, and babul bark as bio-mordants to dye cotton yarn. It has been reported that dyeings with sufficient fastness can be obtained. In another study, lemon peel (*C. limetta*) extract as a natural dye, *Punica granatum* (pomegranate rind), *Terminalia chebula* (myrobalan), and *Acacia nilotica* (babul bark) as bio-mordants were used to dye silk fabric. It has been stated that beige-light yellow-green color tones can be obtained depending on the mordant substance (Sangeetha et al. 2015). Mansour et al. (2016) also used pomegranate peel as a bio-mordant. In their studies, they used *Vitis vinifera* L. leaf extract as a natural dye, tannic acid, and pomegranate peel extract as bio-mordants to dye linen and silk fabrics. It has been reported that dyeing in brown tones with sufficient fastness can be obtained. Other researchers using pomegranate peel as a bio-mordant are Adeel et al. (2016). In their research, they used golden *Duranta* (*Duranta repens*) leaf extract as a natural dye, pomegranate peel, and tannic acid as bio-mordants in a microwave environment to dye cotton fabrics. It has been stated that pomegranate peel extract produces dyes with high color strength. In another study, *Terminalia catappa* (tropical almond) leaf extract was used as a natural dye to dye silk fabrics, and *Terminalia chebula*, *Curcuma longa*, tannic acid, and *Punica granatum* were used as bio-mordants. It has been reported that light yellow-orange color tones can be obtained depending on the bio-mordant (Vadwala and Kola 2017). Hosseinnezhad et al. (2017) used pomegranate peels as a bio-mordant in the dyeing of silk fabrics. It has been reported that reddish-blue color tones can be obtained depending on the bio-mordant. In another study in which *A. vasica* extract was used as a natural dye, it was reported that gallnut, pomegranate rind, and babool were used as bio-mordants. It is stated that in acidic, neutral, and alkaline environments, each bio-mordant combination utilized in this study produced tasteful and vibrant colors ranging from light yellow, reddish yellow, and brown to deep brown (Rather et al. 2018). Pomegranate peel was used as a natural mordant in the printing process on cotton fabric. In the study, in which marigold (*Tagetes erecta* Linn.) flower extract was used as a natural dye, gum Arabic and environmentally friendly mordants such as mirobalon and pomegranate peel were used as thickeners (Sangamithirai 2019). Kiran et al. (2020) emphasized that Al, Fe, and tannin can be used as chemical mordants, and pomegranate, *Acacia*, henna, and turmeric can be used as bio-mordants to dye coconut (*Cocos nucifera*) coir and silk in the microwave environment. It has been stated that dyeings with sufficient fastness values in different colors can be obtained with different bio-mordants. According to Adeel et al. (2021a), tannin-containing coconut can be used as a dye, and *Acacia*, henna, pomegranate, and turmeric can be used as bio-mordants in the dyeing of wool fabrics. It has been stated that wool fabrics with sufficient fastness values in different colors can be obtained with different bio-mordants. In a study, *Acacia* bark, pomegranate peel, and mango bark were used as bio-mordants while investigating the coloring potential of durum (*Triticum durum* Desf.) and bread (*Triticum astivum* L.) wheat bark. It has been reported that the use of pomegranate peel and *Acacia* bark is more effective in obtaining high color depth in cellulosic fabrics dyed with an alkaline extract of durum wheat bark (Khan et al. 2021). In another study, *Eleutherine bulbosa* bulbs were used as a natural dye, and myrobalan fruit extract and pomegranate peel extract were used as bio-mordants for dyeing cotton fabric (Kizhakkinayil and Nair 2022). Caffeine, chlorogenic acid, polyphenols, flavonoids, alkaloids, and other phytochemicals are the important ones, while polyphenol (chlorogenic acid) is the main functional moiety. Adeel et al. (2023d) dyed cotton in a microwave environment with the dye obtained from coffee beans and using bio-mordants obtained from turmeric and pomegranate peel extracts. It has been stated that reddish-yellow tones can be obtained from dyeing, depending on the MW rate and bio-mordant. Adeel et al. (2022h) suggested that Esfand (*P. harmala*) seed extract as a natural dye, pomegranate peel, and tannic acid can be used as bio-mordants in the dyeing of polyamide fabric. In general, it has been stated that bio-mordants give better results in terms of color fastness. Rehman et al. (2022a) also used pomegranate peel as a bio-mordant. They used *Alkanna tinctoria* dye as a natural dye, pomegranate peel, and turmeric as bio-mordants for dyeing silk fabric. It has been stated that the main reason for the very good fastness of the samples colored with bio-mordants such as light, washing, and rubbing is that both the colorant and the bio-mordant provide protection against external effects due to their nature. In another study, madder was used as a natural dye, and myrobalan and pomegranate peel were used as bio-mordants for dyeing wool yarns (Hosseinnezhad et al. 2022b). In one study, melanoidin was successfully extracted and purified from *Lycium barbarum* residues, and in the dyeing process, instead of the traditional metal mordant, the eco-friendly bio-mordant pomegranate peel was used to dye wool fabrics. It has been reported that a brown color tone was obtained at the end of dyeing (Wang et al. 2023).

#### Pumpkin (*Cucurbita *spp*.*)

The vegetable pumpkin, which is a member of the Cucurbitaceae family, is grown extensively around the world (Dhiman et al. 2009). Numerous phytoconstituents from the alkaloids, flavonoids, and palmitic, oleic, and linoleic acid categories are present in it (Yadav et al. 2010). Hosseinnezhad et al. (2022d) reported that *Reseda luteola* and madder can be used as natural dyes in the dyeing of wool yarns, and pumpkin extract can be used as a bio-mordant. It was stated that the fastness properties of dyed yarns increased significantly with mordanting.

#### Pyrus pashia

In temperate areas of the Northern Hemisphere, *Pyrus pashia*, a medium-sized fruiting tree belonging to the Rosaceae family, is sometimes referred to as Indian pear, Himalayan pear, or Mehal. It has about 38 species. After pharmacognostic research on the plant, the presence of secondary metabolites such as alkaloids, flavonoids, sterols, triterpenoids, and phenolic compounds was revealed (Ali and Juyal 2018). In one study, Vankar and Shanker (2009) used *Delonix regia* flower extracts together with enzymes (protease, amylase, diasterase, and lipase) as a natural dye source for silk dyeing and *Pyrus pashia* fruit, which is copper-containing, as a bio-mordant source. It has been stated that enzymatic and bio-mordant treatments can impart good color strength to silk fabric using the *Delonix* flower as a dye source.

#### Quebracho (*Schinopsis spp*.) tree bark

Tannic acid can potentially be produced from the wood of the Quebracho tree (Vera et al. 2022). One of the rarest bio-mordants is this resource. In one study, nut shell extract was used as a natural dye, and Quebracho bark tree extract was used as a bio-mordant for dyeing wool/angora blended yarn. It is stated that the samples bio-mordanted with Quebracho exhibit significantly increased color fastness to light for both radiation levels examined (Stanculescu et al. 2017).

#### Quercus castaneifolia

There are phenolic acids, tannins, flavonoids, lignans, stilbenoids, coumarins, monoterpenes, triterpenes, and steroids in the *Quercus* L. (Fagaceae) genus (Şöhretoğlu and Renda 2020). In another study, madder (*Rubia tinctorum* L.) extract was used as a natural dye for dyeing wool yarns, and *Rhus coriaria*, *Eucalyptus*, *Terminalia chebula*, *Quercus castaneifolia*, and pomegranate extracts were used as bio-mordants (Jahangiri et al. 2018).

#### Rhizophora mucronata fruits

In one study, manjistha was used as a natural mordant along with myrobalan, pomegranate rind, and *Rhizophora mucronata* fruits. It is reported that the fruits of *Rhizophora mucronata* function as a better mordant than the other natural mordants when they are utilized (Praveena et al. 2014).

#### Rose (*Rosa indica *L*.*)

*Rosa indica* L. is a species of perennial flowering shrub. It is a member of the Rosaceae family, which includes rhizomatous, thorny, or climbing herbs, shrubs, and trees. In one study, quinic acid, 5-hydroxymethylfurfural, pyrogallol, levoglucosan, and 4H-pyran-4-one,2,3-dihydro-3,5-dihydroxy-6-methyl were identified as the major components in the methanolic extract of indica petals (Rasheed et al. 2015). Using a natural dye made from cinnamon bark (*Cinnamomum verum*), Adeel et al. (2020b) investigated the fixing characteristics of *Acacia* (*Acacia nilotica*), henna (*Lawsonia inermis*), turmeric (*C. longa*), pomegranate (*P. granatum*), and rose (*Rosa indica*). It is stated that the conjugation of plant-derived anchors and bio-mordants, as well as the production of extract H-bonding, all play important roles in the establishment of shade when utilizing extracts of bio-mordants.

#### Rue herb (*Ruta graveolens*)

Thakker and Sun (2022) reported that hops can be used as a natural dye in dyeing cotton fabric and as natural mordant agents in oak bark, mugwort herb (*Artemisia vulgaris* L*.*), rue herb (*Ruta graveolens*), and black cherry stem (*Prunus serotina*). It is reported that very good rubbing and washing fastnesses and moderate light fastness values were achieved for the dyed samples.

#### *Rumex hymenosepalus*

*Rumex* species are abundant in anthraquinones, naphthalenes, flavonoids, stilbenoids, triterpenes, carotenoids, and phenolic acids, according to numerous phytochemical studies on this genus (Vasas et al. 2015). Large amounts of tannin found in *Rumex hymenosepalus* roots are used as a bio-mordant in the dyeing of wool. In one study, *Berberis vulgaris* wood extract was used as a natural dye for dyeing wool yarns, and tannin from the roots of *Rumex hymenosepalus* was used as a bio-mordant. It is stated that when applied as a bio-mordant to wool, the tannin found in the roots of *Rumex hymenosepalus* strengthened the color of the dyed items (Haji 2010).

#### Sepalika (*Nyctanthes arbor*-*tristis*) flowers

*Nyctanthes arbor-tristis*, a member of the Oleaceae family, produces sepalika flowers. The flower has an orange calyx and white petals. It has been discovered that *Nyctanthes* stems are a great source of antioxidants. The flower’s aqueous extracts, calyx, and petals were tested for their ability to fix colors to fabric when used for cotton dyeing. In one study, cotton was dyed using natural dye sources such as Chinese rose, Aparajitha flower, and balsam flower, as well as sepalika (*Nyctanthes arbor-tristis*) as a bio-mordant. It has been stated that bio-mordants such as sepalika can be used to improve the fastness properties of dyed fabrics (Wijayapala 2020).

#### Snep sohmylleng (*Tsuga canadensis* L. Carr.)

In one study, Banerjee et al. (2018) used tea leaves, onion peel, land ac insect as natural dyes, Sohtung leaves (*Terminalia chebula*), *Tsuga canadensis* L. Carriere (snep sohmylleng), *Oroxylum indicum* L. Kurz (waitlam pyrthat), and *Baccaurea ramiflora* Lour. (Sohkhu leaves) as bio-mordants to dye silk yarns. It has been reported that dyeing in burgundy-gray-brown tones can be obtained depending on the bio-mordant variety.

#### Sohkhu (*Baccaurea ramiflora* Lour.) leaves

In one study, Banerjee et al. (2018) used tea leaves, onion peel, land ac insect as natural dyes, Sohtung leaves (*Terminalia chebula*), *Tsuga canadensis* L. Carriere (snep sohmylleng), *Oroxylum indicum* L. Kurz (waitlam pyrthat), and *Baccaurea ramiflora* Lour. (Sohkhu leaves) as bio-mordants to dye silk yarns. It was reported that dyed silks had sufficient fastness values.

#### Sumac (*Rhus coriaria *L*.*)

The sumac species *Rhus glabra* is frequently referred to as “rhubarb.” Sumac is a spice widely used in Turkey and the Middle East. The fruits have one seed and are red in color. Due to their high tannin content, their dried and crushed leaves have been employed as tanning agents. This plant’s leaves were found to contain flavones, tannins, anthocyanins, and organic acids, according to previous phytochemical investigations (Kosar et al. 2007). Due to its rich chemical content, different studies have been found on the use of sumac both as a natural dye and as a bio-mordant. Dalby (1993) pointed out that oak galls, sumac, barks, and bark resins are all suitable sources of tannin that can be used for fixing natural dyes to cellulose fibers. It has also been stated that the amount of tannin obtained from these natural products may vary from source to source, and the use of natural tannin as a suitable stabilizer would be more acceptable than its synthetic counterpart when producing completely environmentally friendly yarns. Hosseinnezhad et al. (2018) used madder and reseda as natural dyes and sumac as a bio-mordant to dye silk fibers. It is stated that the dyeing’s wash, light, and rubbing fastness were all improved by the application of green mordanting. In another study, myrrh (*Commiphora molmol*) extract was used as a natural dye for dyeing wool and silk, and sumac (*Rhus coriaria*) and manjakani (*Quercus infectoria*) were used as bio-mordants (Salem and Al Amoudi 2020).

#### Supari (*Areca catechu*)

*Areca catechu*, sometimes referred to as Supari, is a type of dried, ripe nut that belongs to the Arecaceae family and is grown in India and Southeast Asia’s tropical regions. Tannins are the main component of *A. catechu* (Ansari et al. 2021). In one study, teak leaf extract as a natural dye, harda (*Terminalia chebula*), *Eucalyptus* (*Eucalyptus globulus*) leaves, Supari (*Areca catechu*), iron filings and jaggery, pomegranate peel (*Punica granatum* L.), tamarind (*Tamarindus indica*), and amla (*Phyllanthus emblica*) as bio-mordants were used to dye selected natural and synthetic fabrics. It has been stated that colors in purple, pink, and burgundy tones can be produced depending on the bio-mordants (Agrawal and Chopra 2020).

#### *Vernonia amygdalina *leaves

In one study, Jabar et al. (2022) used *Justicia carnea* L. leaves, *Moringa oleifera* L. bark, *Azadirachta indica* L. bark, and *Vernonia amygdalina* leaves as bio-mordant agents to dye cotton fabric with natural dyes. It has been stated that pink-brown colors can be obtained depending on the bio-mordant.

#### Waitlam pyrthat (*Oroxylum indicum* L.) Kurz

In one study, Banerjee et al. (2018) used tea leaves, onion peel, and land ac insect as natural dyes, Sohtung leaves (*Terminalia chebula*), *Tsuga canadensis* L. Carriere (snep sohmylleng), *Oroxylum indicum* L. Kurz (waitlam pyrthat), and *Baccaurea ramiflora* Lour. (Sohkhu leaves) as bio-mordants to dye silk yarns. It was reported that dyed silks had sufficient fastness values.

#### Wheat bran

Another interesting bio-mordant used in natural dyeing is wheat. Baseri and Ahmadzadeh (2022c) reported that color fastness increases when wheat bran and citric acid are used as bio-mordants in addition to natural dyes to dye wool. Citric acid and wheat bran could be employed as bio-mordants to enhance the dye fastness qualities of wool yarns and boost their affinity for the dye extract.

#### White mulberry (*Morus alba *L.) fruit

Red mulberry was first cultivated in North and South America, black mulberry in Southern Russia, and white mulberry in Western Asia, which includes Turkey. Mulberry fruits are among the berries that are high in phenolics and anthocyanins (Gungor and Sengul 2008). Hussaan et al. (2017) used bio-mordants (*Morus alba* fruit, *Acacia nilotica* bark, and *Curcuma longa*) as well as chemical mordants to dye cotton fabrics with natural dyes. It is stated that the use of bio-mordants enhanced the color intensity and gave cotton materials new colors, and during bio-mordanting, *A. nilotica* bark extract outperformed *M. alba* fruit and *C. longa* tuber as pre-mordants in terms of color strength.

#### Willow (*Salix alba *L*.*)

One of the most well-known and widely distributed species in the *Salix* genus is *Salix alba* L. It has been used as a medicine since ancient times and is rich in many biologically active chemicals, particularly phenolic compounds (Vasfilova 2020). The presence of at least 11 related salicylate compounds, including salicin, saligenin, salicylic acid, isosalicin, salidroside, picein, triandrin, salicoylsalicin, salicortin, isosalipurpuroside, and salipurpuroside, has been demonstrated by HPLC mass spectrometry analysis of aqueous extracts of willow bark (Shara and Stohs 2015). In one study, *Quercus robur* L. (fruit cups) extract was used as a natural dye, and *Salix alba* L. wood extract was used as a bio-mordant for dyeing wool and pashmina fabrics. It is reported that the extracted dye displayed a diversity of color tones when used in combination with natural mordants using various mordanting techniques (Geelani et al. 2015). In another study, Kavak et al. (2010) used onion peel (*Allium cepa*) as a natural dye, willow (*Salix alba*) extract as a bio-mordant, and AAUS (Artifical Animal Urine System) together to dye wool, cotton, feathered-leather fibers, and wood materials. It is reported that the brilliance of natural fibers and wood products is contributed by willow extract in addition to AAUS.

#### Tamarind (*Tamarindus indica *L.)

**Fig. 6:**
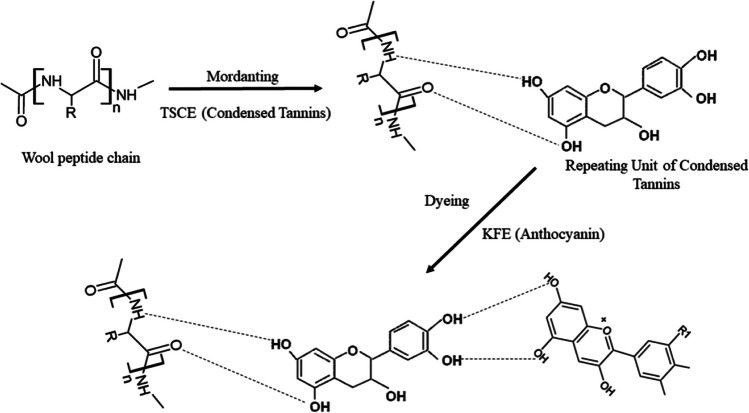
Complex structure between wool, bio-mordant, and coloring matter (this figure was adapted from Singh et al. 2019)

*Tamarindus indica* L. belongs to the leguminaceae family and the subfamily Caesalpinaceae. The genus *Tamarindus* is monotypic, meaning it contains a single species. *Tamarindus indica* is commonly known as tamarind. Tamarind grows in more than 50 countries in Africa, Asia, and Central America and is common across the tropics and subtropics (Yahia and Salih 2011). Tamarind seed kernels contain a comparatively high phenolic content and antioxidant activity (De Caluwé et al. 2010). In addition, tannin was also extracted from the tamarind seed coat, and the tannin class was determined (Belemkar and Ramachandran 2015). Tamarind has been used as a bio-mordant in addition to natural dyes in many different studies. In one study, mango (*Mangifera indica* L.) seed was used as a natural dye for dyeing cotton fabric, and *Tamarindus indica* seed was used as a bio-mordant. It is claimed that the treated fabrics mixed with natural mordant were more effective in terms of antibacterial activity against the two tested microorganisms (Satirapipathkul et al. 2014). In another study, turmeric, pomegranate peel as natural dyes, and tamarind seed coat tannin as a bio-mordant were used for dyeing cotton, wool, and silk fabrics. It is stated that the tamarind seed coat mordant alone can be used to create fabrics with good antibacterial action (Prabhu and Teli 2014). Sheikh et al. (2016) reported that turmeric and henna as natural dyes and harda and tamarind seed coat as bio-mordants can be used for dyeing wool fabrics. It is reported that with changing mordant and natural dye concentrations, several hues ranging from light to deep can be produced. In another study, Mariselvam et al. (2018) used Pterocarpus santalinus tree extract as a natural dye and tamarind as a bio-mordant for dyeing natural fibers. It is stated that pale red-burgundy colors can be obtained in dyeings made using tamarind. Singh et al. (2019) used kapok flower extract as a natural dye and tannin-based tamarind seed coat extract as a bio-mordant to dye wool. The relationship between wool, bio-mordant, and coloring matter is intricate, as shown in Fig. 6. In another study, the extract obtained from the dry leaves of *Tridax procumbens* as a natural dye to dye silk was used as a bio-mordant, gall nut (*Quercus infectoria*), dried fruits of amla (*Phyllanthus emblica*), dried fruits of bahera (*Terminalia bellirica*), myrobalan (*Terminalia chebula*), tamarind seed coat (*Tamarindus indica*), and laboratory-grade tannic acid were used. It is stated that, in comparison to the unmordanted sample, all of the bio-mordants utilized in the investigation produced a significant improvement in color strength (Sudhakar 2020). Marikani et al. (2020) used *Bixa orellana* extract as a natural dye in the dyeing of cotton fabric and tamarind seed powder as a bio-mordant. In another study, turmeric powder was used as a natural dye to dye silk fabric, and tamarind (*Tamarindus indica* L.) seed coat tannin was used as a bio-mordant. It is reported that the color strength value and general fastness attributes are enhanced by tannin mordanting (Sarker et al. 2020). Baseri (2022b) also used tamarind in his study. In that study, he used marzangoosh leaves as a natural dye to dye cotton threads and tamarind hull as a bio-mordant.

**Fig. 7:**
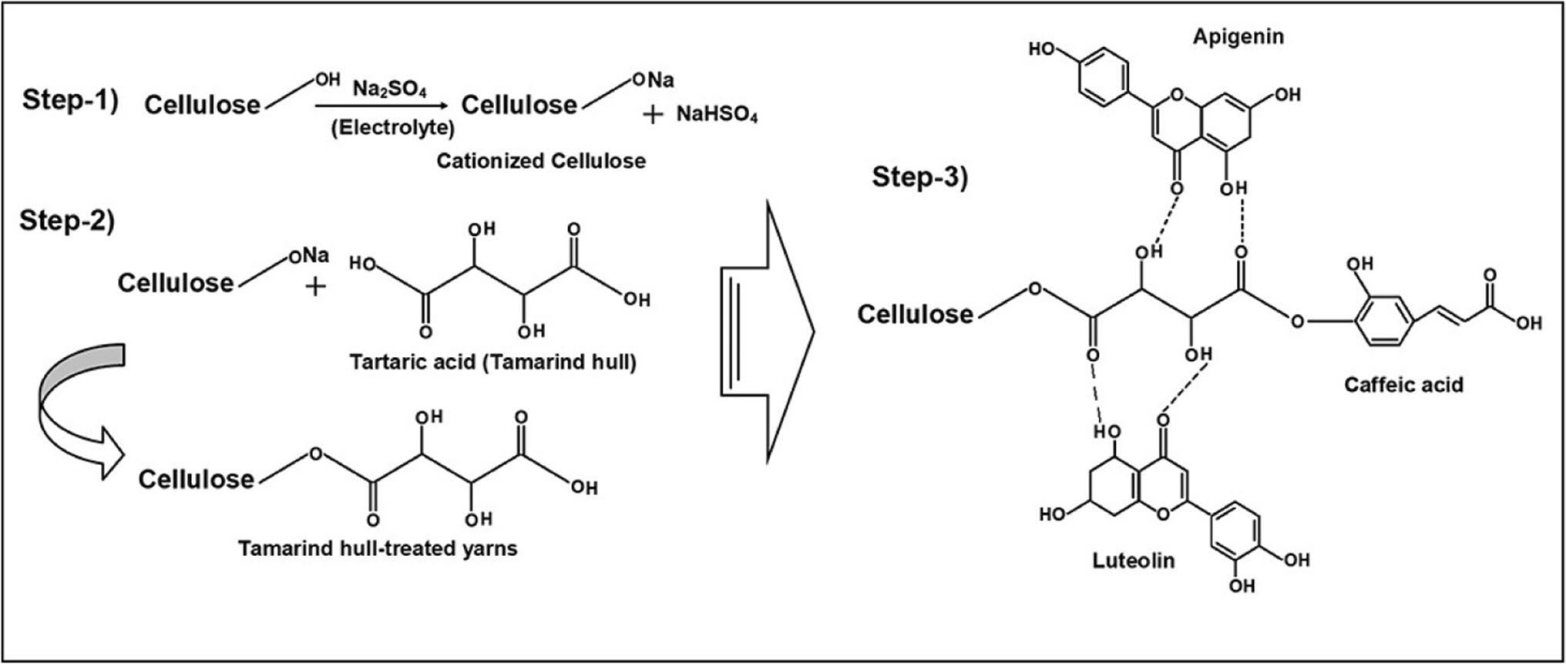
Complex structure between bio-mordant, cellulose, and natural dye (this figure was adapted from Baseri 2022b)

Figure 7 shows a potential process for cationizing cotton (step 1), interacting tamarind shell with cationized cotton during pre-mordanting (step 2), and reacting marzangoosh molecules with tamarind hull-treated cotton strands (step 3) (Baseri 2022b). Nazir et al. (2022) used sugarcane meal, wheat bran, and rice husk as natural dyes and *Tamarindus indica* L. seed coat extract as a bio-mordant to dye cotton fabric in their study. It has been stated that colors with sufficient fastness can be obtained in light yellow and beige tones.

#### Tannic acid (Tannins) (C_76_H_52_O_46_)

In this section, brief information was given about tannins used as mordant materials. Tannins are used as mordants in dyeing textiles. Numerous naturally occurring polyphenols go by the term “tannin,” which is derived from the French word “tanin” (tanning substance). In nature, tannins are present in many different families of higher plants, including plant galls, chestnut and oak wood, Divi-Divi, sumac, myrobalan, trillo, and valonia. The chemistry of tannins varies significantly depending on where they are found in nature. Nearly every component of the plant, including the bark, wood, leaves, fruit, roots, and seeds, has high levels of tannin. The tannins are glossy, practically colorless, loose masses that are light yellow or white in color and have an astringent flavor and very peculiar smell. Tannins are used to create cationic dyes (tannin dyes) in the dyestuff industry as caustics. Tannins are polyphenolic secondary metabolites of higher plants. Tannins are divided into four main groups: gallo-tannins, ellagitannins, complex tannins, and condensed tannins. Tannins are polyphenolic secondary metabolites of higher plants and are either galloyl esters and their derivatives, in which galloyl moieties or their derivatives are attached to a variety of polyol-, catechin-, and triterpenoid cores (gallo-tannins, ellagitannins, and complex tannins), or they are oligomeric and polymeric proanthocyanidins that can possess different interflavanyl coupling and substitution patterns (condensed tannins) (Khanbabaee and Ree 2001). Rosaceae, Geraniaceae, Leguminosae, Combretaceae, Rubiaceae, Polygonaceae, Theaceae, and other plant families are rich in both of the aforementioned categories of tannins. On the other hand, the members of the families Papaveraceae and Cruciferae are completely free of tannins. Table 2 lists the main plants that produce tannin along with their tannin concentration. Tannins are amorphous, non-crystallisable chemicals. They are soluble in diluted alkalis, water, ethyl alcohol, glycerol, and acetone. They exhibit an acidic response and a strong, astringent flavor in their aqueous solution. The majority of tannin compounds cause solutions of alkaloids, glycosides, gelatin, and heavy metal salts of copper, lead, and tin to precipitate (Prabhu and Bhute 2012).

**Table 2:** Some tannin-producing plants and their tannin content (Prabhu and Bhute 2012)

Botanical name (common name)	Parts	Tannin content (%)
*Acacia catechu* (*khair*)	Wood	57–60
*Acacia mollissima* (*mimosa*)	Bark	35–65
*A. mearnsil* (*black wattle*)	Bark	35
*A. nilotica* (*babool*)	Bark	12–18
*Anogeissus latifolia* (*dhawada*)	Leaves	16–18
*Anacardium occidentale* (*cashew*)	Leaves	20–25
*Astronium balansae* (*urun day*)	Wood	10
*C.coriaria* (*Divi-divi*)	Pods	35–50
*Cassia auriculata* (*avaram*)	Bark	15–20
*Ceriops roxburghiana* (*goran)*	Bark	20–40
*C. fistula* (*amaltas*)	Bark	9–12
*Castanea spp.* (*chestnut*)	Wood	30
*Casuarina equisetifolia* (*Casuarina*)	Bark	7–8
*Ceriops roxburghiana* (*Goran*)	Bark	20–40
*C. Tagal* (*Goran*)	Bark	20–40
*Cleistanthus collinus* (*karad*)	Bark	23–27
*Emblica officinails* (*amala*)	Stem bark, fruit, leaves	8–9, 21–24
*Ecualyptus occidentalis* (*mallet*)	Bark	40
*Eucalyptus spp.* (*Eucalyptus*)	Bark and wood	55
*Eugenia jambolana* (*jamun*)	Bark	13–19
*Hopea parviflora* (*Hopea*)	Bark	21
*Larix spp.* (*larch*)	Bark	10
*Mangifera indica* (*mango*)	Bark	17
*Peltophorum ferrugineum* (*peltophorum*)	Bark	20–22
*Pithecelobium dulce* (*jungle jalebi*)	Bark	30–35
*Punica granatum* (*pomegranate*)	Fruit rind	26
*Quebracho colorado*	Heart wood	20–27
*Quercus aegilops* (*oak*)	Bark	30
*Q. marolepis* (*valonia*)	Cup and bread	23–35
*Q. montana* (*chestnut oak*)	Bark	6–15
*Rhizophora mucronata* (*mangrove)*	Bark	30
*Rhus. spp.* (*sumach*)	Leaves	20–35
*Rhus pentaphylla* (*tizrah*)	Roots and wood	30
*Shorea robusta* (*sal*)	Bark	7–9
*Terminalia alata* (*saja, laurd*)	Bark	15
*T. arjuna* (*Arjun*)	Bark	23
*T. bellirica* (*beheda*)	Nut	12
*T.chebula* (*myrobalan*)	Nut	30–55
*T.tormentosa*	Fruit	10–23
*T. suga* (*hemlock*)	Bark	25
*T. heteropkylla* (*West.hemlock*)	Bark	25
*Tamarindus indica* (*tamarind*)	Fruit	20
*Uncaria gambir* (*gambier*)	Leaves	35–40

Tannins are important in textile dyeing for a number of reasons, including their usage as a mordant in dyeing, the production of ink, the sizing of paper and silk, and for printing cloth. In the beginning, they create vital mordants for dying plant fibers like cotton and linen. Second, they frequently work with the yellow, orange, red, and violet colorants in plants. Their own pigment in the dye bath intensifies these colorants’ hues. Tannin-dyed materials have good light- and wash-fastness (Prabhu and Bhute 2012). Many researchers used tannic acid as a mordant agent when dyeing textile materials with natural dyes (Ali et al. 2010; Arroyo-Figueroa et al. 2011; Prabhu and Bhute 2012; Tayade and Adivarekar 2013; Narayanaswamy et al. 2013; Ibrahim et al. 2013; Mulec and Gorjanc 2015; Kundal et al. 2016; Ebrahimi and Gashti 2016; Swamy et al. 2016; Basak et al. 2016; Mansour et al. 2017; Adeel et al. 2017b; Mariamma and Jose 2018; Baaka et al. 2019; Mir et al. 2019; Elsahida et al. 2019; Gulzar et al. 2015; Azeem et al. 2019a; Saleh and Mohamed 2019; Kawlekar et al. 2022; Sadeghi-Kiakhani et al. 2022b; Li et al. 2022; Ke et al. 2023). In another study, tannic acid, used in denim dyeing, was used as an environmentally friendly alternative to metal mordants (Eryürük et al. 2023).

#### Taro (*Colocasia esculenta *L*.*)

*Colocasia esculenta* (L.) is an annual herbaceous plant belonging to the Araceae family (Prajapati et al. 2011). C. esculenta has some phenolic compounds, such as anthocyanin like cyanidin and pelargonidin derivatives, and also flavones like apigenin and luteolin derivatives (Gonçalves et al. 2013). Hosen et al. (2021) reported that the cotton fabric was dyed with turmeric (*Curcuma longa* L.) extract as a dye and citrus lemon and *Colocasia esculenta* bulk extracts as bio-mordants, and also that the bio-mordant-pretreated sample’s color strength (*K/S*) was two times greater than the metal-mordanted sample’s.

#### Tea (*Camellia sinensis *L*.*) 

The cultivated evergreen plant known as tea, *Camellia sinensis* L., originated in China and then spread to India, Japan, Europe, and Russia before making its way to the New World in the late seventeenth century. Polyphenols (catechins and flavonoids), alkaloids (caffeine, theobromine, theophylline, etc.), volatile oils, polysaccharides, amino acids, lipids, vitamins (like vitamin C), and inorganic elements (like aluminum, fluorine, and manganese) are just a few of the chemical substances found in tea leaves (Sharangi 2009). It is possible to come across studies in which tea leaves are used as both a dye and a mordant agent in natural dyeing. In one study, madder (*Rubia tinctorum*) was used as a natural dye to dye polyester fabrics, and oak wood ash, green tea, black tea, sumac, and gallnut were used as bio-mordants. It is stated that, in conjunction with chemical and natural mordants, dyeing using *Rubia tinctorum* has produced various hues of orange, brown, pink, and reddish green (Gedik et al. 2014). In another study, madder was used as a natural dye, and green tea was used as a bio-mordant to dye 100% nettle-bast bio-fiber fabric. It is stated that all nettle-dyed fabrics displayed extremely high and marketable wash, dry-rub, alkaline-perspiration, acidic-perspiration, and water-fastness qualities (Yavas et al. 2017). Win et al. (2020) also used beetroot (*Beta vulgaris*), hin-nu-new (*Amaranthus viridis* L.) leaves as natural dyes, and tea leaves (*Camella sinensis*) as a bio-mordant to dye cotton fabrics. It has been reported that, depending on the bio-mordant, colors in gray and beige tones can be obtained. In another study, onion peel (*Allium cepa* L.) as a natural dye and tea leaves, tamarind, *Acacia* bark ash, and *Aloe vera* as bio-mordants were used to dye silk fabric. It is stated that the outcomes of the yellow, red, and brown dyes produced with these bio-mordants were good in terms of colorfastness (Yaqub et al. 2020). In one study, the peels of *P. granatum*, flowers of *Rhus coriaria* (*R. coriaria*), and powdered leaves of *Camellia sinensis* (*C. sinensis*) were utilized as bio-mordants during wool dyeing. It has been reported that fabrics dyed in brown tones with bio-mordants have better color and fastness properties than fabrics dyed with metal mordants (Abou Elmaaty et al. 2023). In one study, three different natural mordants, namely tea leaves, tannic acid, and harde, were used to dye cotton and viscose rayon. It has been reported that colors in gray tones can be produced according to the bio-mordanting method (Madhu and Agrawal 2024).

#### Thuja (*Thuja orientalis *L*.*)

A well-known medicinal plant, *Thuja orientalis*, often called arbor vitae, white cedar, or morpankhi, is a member of the Cupressaceae family. It is believed to have come from Central Asia, Korea, Japan, Taiwan, Siberia, and northern and north-eastern China. Fresh plants contain essential oils, reducing sugars, water-soluble polysaccharides, water-soluble minerals, free acid, tannin-producing substances, flavonoids, saponins, glycosides, and alkaloids, according to biochemical research (Jain and Sharma 2017). İşmal et al. (2014, 2015) reported that this plant could be a bio-mordant for dyeing wool fabric. In their study, they used powders of valex (acorns of *Quercus ithaburensis* ssp. macrolepis), pomegranate (*Punica granatum* L.) rind, rosemary (*Rosmarinus officinalis*), and thuja (*Thuja orientalis* L.) as bio-mordants. It is stated that based on the control sample, *Thuja* was ineffective for both washing and light fastness, while valex and pomegranate rind enhanced both, and rosemary was ineffective for both light and washing fastness.

#### Turmeric (*Curcuma longa *L*.*)

One of the plants used both as a natural dye and as a bio-mordant is turmeric. It is widely cultivated in Asia, primarily in India and China. The Zingiberaceae (ginger) family includes the herbaceous, evergreen plant known as turmeric. Numerous metabolites, including curcuminoid, oil content, flavonoids, phenolics, a few crucial amino acids, protein, and high alkaloid content, are present (Verma et al. 2018). Adeel et al. used turmeric as a bio-mordant in their research at different times. They used henna, turmeric, *Acacia nicotica*, and pomegranate as bio-mordants in their works (Adeel et al. 2019a, 2020a, e, 2021b, 2022f, g, 2023a). Rehman et al. (2022b) used saffron (*Crocus sativus*), safflower (*Carthamus tinctorius* L.) leaves as natural dyes, turmeric, and pomegranate as bio-mordants to dye polyamide fabric. In one study, Salman et al. (2023) highlight the use of aqueous, alkaline, acidic, and basified methanol as solvents to explore roses containing anthocyanin as a source of yellowish-pink natural colorant for wool dyeing. Turmeric and pomegranate were utilized as bio-mordants, while salts of iron (Fe^+2^), aluminum (Al^+3^), and tannic acid (Tn) were used as chemical mordants to increase the colorfastness properties. The findings suggest that wool fabric dyed with naturally occurring rose petals can be used as a valuable antibacterial fabric since it has a variety of bioactive components that counteract the effects of fabrics dyed with synthetic dyes that can cause allergies.

#### Walnut (*Juglans regia *L*.*) shell

The walnut tree belongs to the Juglandaceae family. Due to its excellent kernels, it is the most common nut-bearing tree and is cultivated in several regions of the world. Many different phenols and derivatives have been identified in walnut shell pyroligneous, acids such as phenol, 4-methyl-pheno, 1,2-benzenediol (Jahanban-Esfahlan and Amarowicz 2018). In one study, Mall et al. (2017) used *Butea monosperma* leaves as a natural dye and almond shell, walnut shell, rinds of bahera fruits, and rinds of harad (Myrobalan-*Terminalia chebula*) fruits as bio-mordants to dye cotton fabric. It is stated that the chemical and natural mordants were both found to be equally effective, and all mordanted samples had greater *K/S* values than the control samples, indicating a significant affinity for cotton. In another study, madder and *Reseda luteola* were used as natural dyes for dyeing wool, and walnut husk was used as a bio-mordant. It has been stated that, as a result of dyeing, colors in brown, pink, and burgundy tones can be obtained (Hosseinnezhad et al. 2022c, 2023a).

#### Xylocarpus moluccensis

One of the different bio-mordant materials used in natural dyeing is the bark of *Xylocarpus moluccensis*. In one study, Rahman et al. (2013) used heartwood as a natural dye and the bark of *Xylocarpus moluccensis* as a bio-mordant to dye silk fabric.

#### Zeera (*Cuminum cyminum*)

Due to its unique aromatic effect, *Cuminum cyminum* (cumin), often known as “zeera,” is a significant and well-liked spice that is utilized in food. *C*. *cyminum*’s fruit is made up of volatile oils, proteins, and oil. The volatile oil that makes up 30 to 50% of cumin aldehyde is the primary component, with small contributions from phellandrene, hydrocuminin, α-pinene, hydrated cumin aldehyde, cuminic alcohol, and β-pinene (Mughal 2022). In one study, Adeel et al. (2020c) used saffron and madder as natural dyes and zeera and harmal seeds as bio-mordants to dye woolen yarn. It is stated that zeera and harmal used together with saffron extract showed high color strength and sufficient fastness properties.

### Oil mordants

Oil mordants are naturally occurring oils that contain fatty acids and their glycerides, including palmitic, stearic, oleic, and ricinolic acids (Kumar and Sinha 2004). Stearic, oleic, ricin, and other fats containing fatty acids occur naturally in oil mordants and their glycerides; the main function of an oil mordant is to form a complex and then use it as a mordant (Ragab and Hassabo 2021). Castor oil or Turkey red oil (TRO) are oil-type mordants (İşmal and Yıldırım 2019). It was previously used as an oil mordant in sesame (til) oil (Aggarwal 2021). Oil mordants are used mainly in the dyeing of Turkey red color from madder. The primary purpose of the oil mordant is to create a complex with the principal mordant, alum. Due to the presence of the sulfonic acid group, sulfonated oils have a greater ability to bind metal ions than natural oils (Vankar 2000). Alum is easily removed from treated cloth because it is soluble in water and has no attraction to cotton. Fatty acids like palmitic, stearic, oleic, and others, as well as their glycerides, are present in naturally occurring oil. Fatty acid’s -COOH group reacts with metal salts to form -COOM, where “M” stands for the metal. The introduction of the sulphonic acid group, -SO_3_H, caused by the treatment of oils with concentrated sulfuric acid results in sulphonated oils, which have stronger metal-binding capacity than natural oils. Metal and sulphonic acid can react to form -SO_3_M. With a mordant dye like madder, this bonded metal can form a complex to produce Turkey red, a color with exceptional fastness and hue. Turkey red oils (TRO) was the name given to the sulphonated oils as a result. These days, only sulphonated castor oil is referred to as TRO (Prabhu and Bhute 2012).

### New-generation and non-vegetable-based mordants

The new-generation and non-vegetable-based mordant (Table 3) materials have also interestingly entered the natural dyeing industry. It has been reported here that many different materials can be used as mordant agents in natural dyeing processes.

**Table 3:** Sources used as non-vegetable-based mordants in the literature

No	Non-vegetable-based mordants	References	No	Non-vegetable-based mordants	References
1	Chitosan	Kampeerapappun et al. (2011) Lu (2011) Teli et al. (2013) Saravanan et al. (2015) Mehrparvar et al. (2016) Azarmi and Ashjaran (2017) Chilukoti et al. (2019) Safapour et al. (2019) Alebeid et al. (2020) Syrine et al. (2020) Ul-Islam and Butola (2020) Zhao et al. (2020) Kandasamy et al. (2021) Krifa et al. (2021) Dulo et al. (2022) Ltaief et al. (2022) Sadeghi-Kiakhani et al. (2022a) Toprak-Cavdur et al. (2022) Lambrecht et al. (2023) El Sayed et al. (2023) Grande et al. (2023) Rahman et al. (2023) Do et al. (2023) Fang et al. (2023)	2	Citric acid	Bulut and Akar (2012) Bulut et al. (2014) Karaboyacı and Uğur (2014) Park and Jung (2014) Ammayappan and Shakyawar (2016) Zhang et al. (2020) Perju et al. (2019) Kaynar and Ucar (2019) Yıldırım and İşmal (2019) Ismal and Yıldırım (2020) Gong et al. (2020) He et al. (2021) Oancea et al. (2021) Baseri and Ahmadzadeh (2022c) Pancapalaga et al. (2022) Tu et al. (2023)
3	Wood ash	Kampeerapappun et al. (2011) Cunningham et al. (2011) Tajuddin et al. (2011) Ahmad et al. (2012a) Gedik et al. (2014) Geelani et al. (2017) Yaqub et al. (2020) Yan et al. (2021)	4	Gelatin	Bydoon (2016) Ahmed et al. (2020a) Ahmed et al. (2020b) El-Zawahry et al. (2021) El-Sayed et al. (2022)
5	Clays	Liu and Chen (2012) Gashti et al. (2014) Pour et al. (2020b)	6	Rare earth compounds	Zheng et al. (2011) Liu et al. (2011) Liu and Bai (2012)
7	Hyperbranched polymers	Davulcu (2015) Mehrparvar et al. (2016) Sadeghi-Kiakhani et al. (2022a)	8	Cow dung	Saravanan and Chandramohan (2011) Chandra et al. (2012)
9	Sodium alginate	Ghaheh et al. (2022) Tehrani et al. (2023a)	10	Vinegar	Geetha and Sumathy (2013) Kashyap et al. (2016)
11	Whey	Başaran and Sarikaya (2015) Baseri (2020)	12	Lime	Kampeerapappun et al. (2011) Feiz and Norouzi (2014)
13	Caffeic acid	Phan et al. (2020)	14	Dorema ammoniacum gum	Haji et al. (2023)
15	Egg shells	Chan et al. (2002)	16	Gum rosin	Yan et al. (2021)
17	Human hair keratin	Baseri (2022d)	18	Potassium acetate	Baig et al. (2019)
19	Jaggery	Agrawal and Chopra (2020)	20	Ammonium acetate	Baig et al. (2019)
21	Lye	Başaran and Sarikaya (2015)	22	Mud	Masae et al. (2017)
23	Mushroom	Başaran and Sarikaya (2015)	24	Oyster shells	Kim et al. (2005)
25	Sodium citrate	Baig et al. (2019)	26	Urine	Ado et al. (2014)
27	Soy protein	Periyasamy (2022)	28	Al-hyperaccumulating plants (*Symplocos* species)	Cunningham et al. (2011)
29	Yeast	Başaran and Sarikaya (2015)			

#### Al-hyperaccumulating plants

Some plants are known to contain certain metals. Such plant sources can be used as bio-mordanting agents in natural dyeing processes. For example, plants that accumulate aluminum have been used as aluminum mordants. As an organic source of mordants, metal hyperaccumulating plants offer improved adherence to the dyes since they tightly chelate to the dye molecules. The use of Al-hyperaccumulating plants as mordants is common in Indonesia. It is stated that traditional dyeing processes in Indonesia have used Al mordants obtained from plants or metallic salts of iron found in mud. It is stated that most of the world’s 250 *Symplocos* species, which are characterized as Al-hyperaccumulators, could probably be used as natural mordants. These natural metallic mordants could be employed as an alternative to mordants that include hazardous metal ions (Cunningham et al. 2011).

#### Ammonium acetate

Some organic compounds are used directly as organic mordant agents in reactive dyes. Baig et al. (2019) used sodium citrate, ammonium acetate, and potassium acetate as organic mordants in their study. It is reported that out of these three salts, sodium citrate demonstrated superior fastness characteristics in comparison to other salts, while organic salts displayed greater *K/S* ratios at lower concentrations.

#### Caffeic acid

Phan et al. (2020) used anthocyanins in blueberry waste as a dyestuff source and reported that cotton materials could be dyed by using caffeic acid as a bio-mordant agent. At the same time, they reported that there are very small interaction energies for dye molecules related to bio-mordants. Gallic acid and caffeic acid were chosen to reconstruct the stability achieved by co-pigmentation, or π-π interactions.

#### Chitosan

**Fig. 8:**
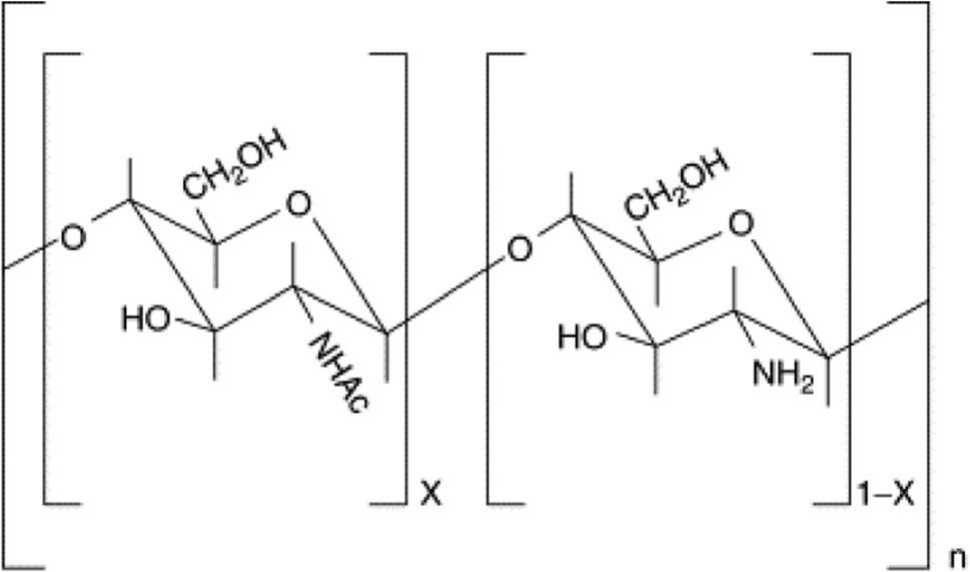
Chemical structure of chitosan with *x*, degree of acetylation; *n*, number of sugar units per polymer (this figure was adapted from Dodane and Vilivalam 1998)

A significant amount of research on this polymer and its possible applications has been published over the past few years. It is easily obtained by deacetylation of chitin, a polysaccharide commonly found in nature (e.g., crustaceans, insects, and some fungi). Chitosan is a linear copolymer of β (1–4) linked 2-actamido-2-deoxy-β-d-glucopyranose and 2-amino-2-deoxy-β-d-glycopyranose (Fig. 8). Chitosan is better suited for industrial uses because chitin is only partially soluble in aqueous solutions. Chitosan is biocompatible with both healthy and sick human skin and has no irritating or allergic effects (Dodane and Vilivalam 1998).

**Fig. 9:**
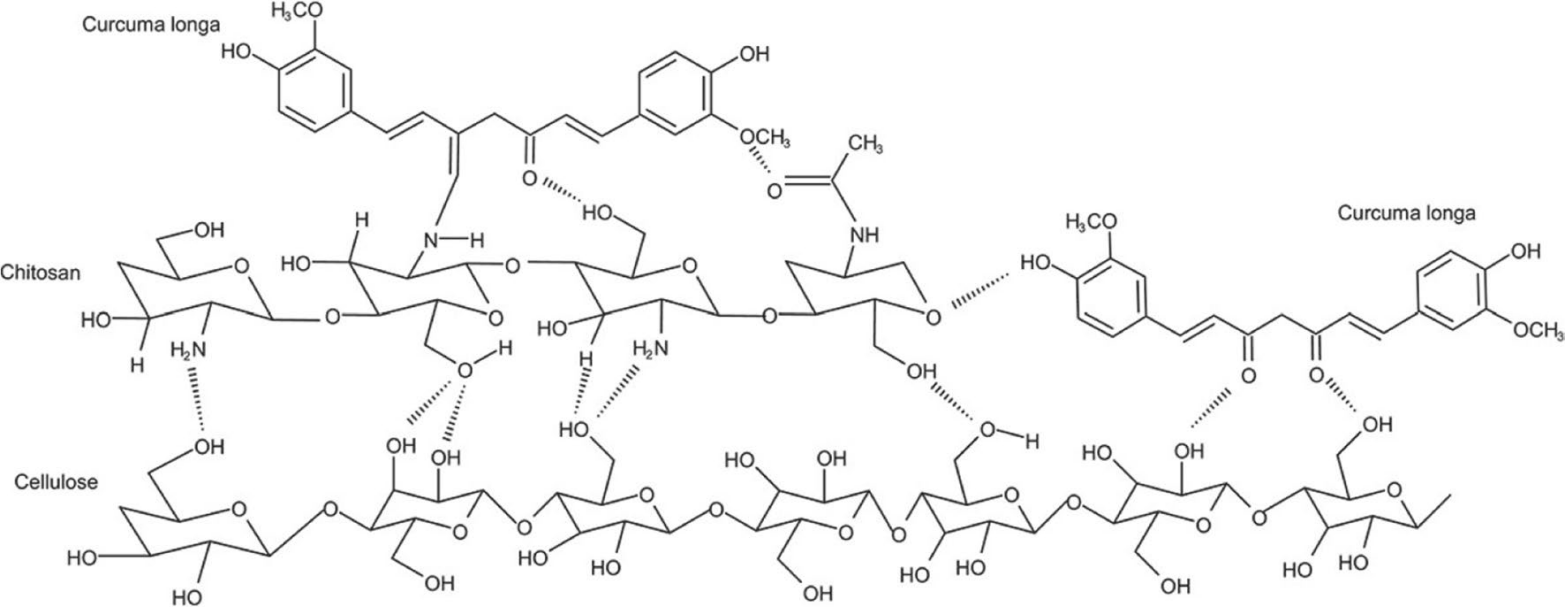
Mechanism of the chitosan-cellulose-*Curcuma longa* reaction (this figure was adapted from Toprak-Cavdur et al. 2022)

There are many different applications of chitosan in the textile industry, but in this section, the use of chitosan as a bio-mordant in the coloring of textile materials along with natural dyes has been investigated. Kampeerapappun et al. (2011) treated knitted cotton fabric with different concentrations of chitosan to find a suitable concentration for the dyeability of *Ruellia tuberosa* Linn, investigated the dyeing properties of chitosan and mordants, and examined the effects of mordanting methods on dyeing. It is stated that fabric that had been treated with chitosan and dyed with popping pods displayed a yellowish-brown hue when mordanted with stannous sulfate, manganese sulfate, sodium chloride, lime juice, and ash. It has been stated that silk fiber can be dyed in various shades by using anthocyanins extracted with an ethanol solution from red onion peels as a natural dye source and chitosan and some metal salts (Fe^2+^, Fe^3+^, Mg^2+^, Al^3+^, Cu^2+^, Mn^2+^) as mordants. It is reported that using chitosan and various salts of transition elements as mordant agents, the different colors of dusky brown, brown, faint yellow, light green, reddish brown, cyan, and green could be obtained (Lu 2011). In another study, it was reported that using chitosan at different concentrations as a mordant, cotton can be printed on fabric with natural dyes such as catechu, turmeric, and marigold. It is stated that despite the fact that the color values varied depending on the dye and mordant combination, the chitosan mordant displayed good color values (Teli et al. 2013). Saravanan et al. (2015) treated cotton fabric with different concentrations of chitosan to find a suitable concentration for dyeability with a natural dye obtained from the bark of *Odina wodier* Roxb, and they found that fabrics treated with chitosan had higher dyeability and fastness values. In one study, Azarmi and Ashjaran (2017) stated that silk fabrics can be colored using inorganic salts, the biological mordant chitosan, and curcumin as a natural dye. It has been stated that it can show sufficient fastness and antibacterial properties in silk fabric dyeing using chitosan. Safapour et al. (2019) suggested that when wool yarn is treated with the prepared chitosan-cyanide chloride hybrid (Ch-Cy as a bio-mordant), dye uptake will increase, dyeing time and temperature will decrease, and Ch-Cy may be an environmentally friendly product. Ul-Islam and Butola (2020) reported that the combination of chitosan with *Citrus sinensis* bark extract biomolecules offers full potential in natural dyeing technology as an approach to improving the natural dyeing performance of cotton fabric. According to Zhao et al. (2020), the structure boosts color fastness characteristics by acting as a bio-mordant by curing cotton fabric with a double-layered chitosan coating and producing a protective layer on it. It is reported that the wash fastness of cotton fabric dyed with sodium copper chlorophyllin can be increased from 3 to 5 by adding a second chitosan layer and curing it using the cross-linking technique. Another study proposed an environmentally friendly process in which wool fibers are dyed in the presence of chitosan using natural dyes extracted from henna leaves in decamethyl cyclopentasiloxane. An efficient, eco-friendly method that can be used to avoid using excessive amounts of water during the dyeing process and lessen heavy metal contamination is to dye chitosan-treated wool fibers with henna extract in a decamethyl cyclopentasiloxane medium (Alebeid et al. 2020). Syrine et al. (2020) examined the treatment of cotton fabric with different amounts of chitosan biopolymer, dimethyl dialyl ammonium chloride, dialylamine copolymer, and alum and emphasized that the dyeing behavior and color fastness values were good with an ecological extract of *Pistacia vera* hull by-products. In another study, logwood dye was applied to a polyester fabric treated with atmospheric plasma. The fabric is treated with environmentally friendly substances such as a formaldehyde-free acrylate binder and chitosan. It has been reported that dyeing plasma-treated polyester fiber while imparting antibacterial properties with a bio-based log wood dye without the addition of any metallic mordant may be a viable eco-option to replace some hazardous dyes and intermediates used in textiles (Krifa et al. 2021). In a study, during the investigation of *Limoniastrum monopetalum* leaves as an ecological dye source in cotton fabric dyeing, alum, chitosan, and pomegranate peel were used as mordants, and the fastness properties of the colored cotton samples obtained at the end of dyeing were found to be good. It has been stated that cotton fabrics colored with natural dye in the presence of chitosan can be obtained in dark yellow and brown tones (Ltaief et al. 2022). Dulo et al. (2022), while investigating the coloring potential of the extracts obtained from the shells of peanut (*Arachis hypogaea* L.), cashew nut (*Anacardium occidentale* L.), coconut (*Cocos nucifera* L.), and macadamia nut (*Macadamia integrifolia* L.), used chitosan, alum, and ferrium sulfate as mordant agents. Toprak-Cavdur et al. (2022) stated that dyeing recycled cotton fibers with natural dyes from *Curcuma longa* and *Pterocarpus santalinus* and bio-mordant chitosan. It is stated that according to the dye, different molecular weights of chitosan had varying effects on the color depth. The mechanism of the complex reaction between chitosan, cellulose, and *Curcuma longa* is given in Fig. 9.

Lambrecht et al. (2023) used red tea as a natural dye and chitosan as a bio-mordant in their study. It has been stated that pink color tones can be obtained by dyeing with chitosan. In a study, it was reported that when onion skin is used as a natural dye, and chitosan is used as a mordant in dyeing cotton, fabrics in red and purple colors and with sufficient washing and rubbing fastness can be obtained (Grande et al. 2023). In one work, a sustainable method for dying linen with natural lac dye using microwave heating following cationic bio-mordant treatment with chitosan is introduced. It is stated that fabrics with close to black color tones and sufficient levels of washing and perspiration fastness can be produced from the dyeings obtained (El Sayed et al. 2023). In one study, Rahman et al. (2023) designed an eco-friendly dyeing process for cotton fibers using the natural dye curcumin and bio-mordant chitosan. According to one study, Do et al. (2023) used chitosan as a bio-mordant together with a dye extract from the roots of *Rubia cordifolia* L. to functionalize silk with antibacterial and UV protection qualities. For comparison, the dyeing procedure also included the use of an alum mordant. The dyed silk samples had a good wash, rubbing, and illumination resistance. With maximal inhibitions of 96.71% and 99.08% against *Escherichia coli* and *Staphylococcus aureus*, respectively, and just 2.3% spore germination of white mold (*Aspergillus* spp.), the chitosan-mordanted-dyed silk demonstrated exceptional antibacterial characteristics. Likewise, the chitosan-modified-dyed silk acquired a superb UV protection factor. In one study, Fang et al. (2023) stated that the natural dye was extracted from biomass lotus seedpod waste; the effects of temperature, time, pH, and a metal mordant on fabric dyeing performance were investigated; and it was then utilized for eco-dyeing and functional finishing of cotton fabric along with the bio-mordant chitosan. The chitosan pretreatment-dyed cotton fabric outperformed other fabrics in terms of UV protection factor (UPF value = 63.4) and *E. coli* and *S. aureus* inhibition (99.86 and 99.99% initial inhibition, 99.08 and 98.21% after ten cycles of washing).

#### Citric acid

Due to its low toxicity compared to other acidulants used mostly in the pharmaceutical and food industries, citric acid is in high demand globally. In addition to these uses, citric acid also appears in cosmetics, cleaning supplies, and other products (Soccol et al. 2006). The following are some citric acid-based natural dyeing studies: Bulut and Akar (2012) investigated the ecological dyeing of cotton fabric and wool yarn with bio-mordants by using plant wastes as dyestuffs. Ecological dyeing on cationized cotton fabric and wool yarn, which do not contain metal salts, was obtained by using the residues of rosemary, rose, lavender, and mate tea extracts as natural dyes. The use of citric acid instead of CuSO_4_ as a bio-mordant provided high color strength and moderately good fastness values in cellulose-extracted wool yarn. In another study, natural dyes for the coloring of wool yarns were obtained from extracts of lavender (*Lavandula hybrida*) and broom (*Spartium junceum*) flowers after oil extraction and from the extract of Dimrit grape red wine. During the dyeing of wool yarns with these natural dyes, ecological wool dyeing was obtained by using an ecological mordant (citric acid) (Karaboyacı and Uğur 2014). While examining the natural dyeing method using the *Phytolacca americana* L.-berry, five different mordants were combined: copper acetate, aluminum potassium sulfate, sodium tartrate plus citric acid, ferric II sulfate, and potassium dichromate. It has been determined that the dye extracted from the fruits sufficiently dyes silk fabrics (Park and Jung 2014). In one of the studies, a natural dye called cochineal from *Dactylopius coccus* cacti was extracted by the aqueous extraction method and applied to wool yarn in the presence of five different mordants in single and combination forms. Aluminum sulfate, tin chloride, ferrous sulfate, citric acid, and potassium hydrogen tartrate were used as mordants. It has been stated that at the end of all dyeings, wool yarns with sufficient fastness can be obtained with a wide color palette from red to purple (Ammayappan and Shakyawar 2016). In another study, Zhang et al. (2020) accepted alum (KAl(SO_4_)_2_12H_2_O), calcium acetate (Ca(CH_3_COO)_2_), and citric acid (C_6_H_8_O_7_) as different eco-friendly mordants and came up with the conclusion that wool can be dyed with the extract obtained from buckwheat hull. It was determined that the results obtained from dyeing cotton in the presence of anthocyanins obtained from *Hibiscus sabdariffa* as a natural dye and bio-mordants such as citric and tannic acids were comparable to dyeing using copper sulfate (Perju et al. 2019). Wool carpet yarns were colored with 9 different mordant substances, including citric acid, using 28 different vegetable dye sources obtained from nature, and dyed yarns were reported to have satisfactory color fastness values (Kaynar and Ucar 2019). In an article by Yıldırım and İşmal (2019), they wanted to show the dyeing effect by using banana peel and various mordants in dyeing polyamide fabric, and they said that citric acid, tartaric acid, and oxalic acid can be used as alternatives to metallic mordants. A study was conducted to reveal the potential of using faba bean husk as a natural dye in dyeing polyamide/elastane fabric, to create a color gamut, and to observe the effect of different mordants. Mordant materials such as alum, ferric sulfate, tin II chloride, copper II sulfate, citric acid, tartaric acid, oxalic acid, ammonium sulfate, sodium acetate, alum-iron, iron-tin, and alum-tin were used here to assist dyeing (Ismal and Yıldırım 2020). He et al. (2021) obtained the colored substance from the lotus seedpod, the natural pigment of oligomeric procyanidins, to dye Tussah silk fabric. Subsequently, the dyed samples were treated with different concentrations of citric acid solution to improve their color fastness. Here, they used citric acid as a crosslinking agent to bind fiber and dye molecules. In a study, it was explained that dyeing processes can be performed in the presence of anthocyanins obtained from the extraction of peony (*Paeonia officinalis* L.) flowers and tannic and citric acids as bio-mordants in the dyeing of cotton. At the end of dyeing, it was stated that cotton samples colored in red tones could be obtained with citric acid (Oancea et al. 2021). Pancapalaga et al. (2022) used 20 sheepskin crusts and mangrove bark extracts in their study to evaluate the color fastness and bark quality of eco-printed leather by using various types of mordants in the natural dye of mangrove extract. The study was done in a completely random design (CRD) using materials such as aluminum sulfate, calcium carbonate, citric acid, and iron sulfate as mordants. In one study, Tu et al. (2023) claimed that fabric pretreatment and aftertreatment procedures were an easy and effective technique to increase dye exhaustion and color fastness of cotton fabrics dyed using *Coptis chinensis* (*C. chinensis*) extract. In order to boost the capacity of their anionic sites, the cotton textiles were first treated with critic acid (CA). According to the findings, pretreated cotton fabrics had stronger colors than untreated materials. The colored fabrics treated with water-repellent substances also showed improved washing fastness. The ultraviolet protection factor (UPF) value of colored fabric increased to 349.69, and the antibacterial activity increased by 98%. Additionally, the colored materials showed outstanding waterproof and wrinkle-resistant qualities.

#### Clays

Some of the interesting mordant materials used are clays. Liu and Chen (2012) used mugwort as a natural dye and montmorillonite as a bio-mordant to dye silk fabrics. Gashti et al. (2014) used bentonite-type clays as mordants. They used madder as a natural dye and bentonite-type clays as a bio-mordant to dye wool yarn.

#### Cow dung

Saravanan and Chandramohan (2011) made one of the different bio-mordant applications. They used natural dye obtained from the bark of *Ficus religiosa* L. as dye and myrobalan and cow dung as natural mordants to dye bleached silk fabrics. At the end of the trials, it is stated that orange and pink colors can be obtained with sufficient fastnesses.

#### Dorema ammoniacum gum

According to Haji et al. (2023), they used dragon’s blood resin extract as a natural dye and four different bio-mordants (including peppermint, mugworts (*Artemisia*), *Dorema ammoniacum* gum, and pomegranate rind) as natural mordants to dye nylon fabric with natural dye.

#### Egg shells

In one study, Chan et al. (2002) reported that tea wastes and wilted flower petals as natural dyes, egg shells, tannic acid, and turmeric as bio-mordants can be used to dye wool. It is stated that it is possible to use natural mordants (egg shells, turmeric) extracted from waste to replace harmful traditional mordants.

#### Gelatin

Gelatin is a largely pure protein obtained by thermal denaturation of collagen, the most common protein. Gelatin is a high-molecular-weight polypeptide and important hydrocolloid that has proven popular with the general public and has found use in a wide variety of food products, largely due to its gelling and thickening properties (Mariod and Fadul 2013). In this section, the possibilities of using gelatin, which has been used for different purposes, as a bio-mordant have been compiled. Bydoon (2016) reported that cotton fabrics can be colored with sufficient fastness by using tea leaves as natural dyes and gelatin as a bio-mordant along with metal salts. In a study by Ahmed et al. (2020a), the cotton fabric pretreated with tannic acid and gelatin, which they previously defined as bio-mordants, was produced with madder (CI Natural Red 9), curcumin (CI Natural Yellow 3), rhubarb (CI Natural Yellow 23), and alkanet (CI Natural Red), stating that it can be dyed with natural dyes. Similarly, Ahmed et al. (2020b) stated in the second part of their study that it can be used as a printing paste content in the printing of cotton fabrics treated with gelatin-tannic acid (bio-mordant). A similar study by El-Sayed et al. (2022) first treated cotton fabric with a gelatin-tannic acid combination (as a bio-mordant), then printed with madder using natural and synthetic thickeners.

#### Gum rosin

A gum exudate called gum rosin is obtained from pine trees. Neoabetic acid, palaustric acid, and abiatic acid are the main components (Sigma Aldrich 2023). Yan et al. (2021) have used it as a bio-mordant agent in the natural dyeing process. It is stated that natural mordants have effects on fastness after washing and perspiration that are comparable to those of metallic ones.

#### Human hair keratin

One of the unusual mordant substances is human hair keratin. In one study, Baseri (2022d) reported that keratin from salvaged human hair can be a bio-mordant that improves the dye absorption of extract from *Matricaria recutita* flowers in dyeing cotton fabrics. It is stated that the color strength and fastness values of the material treated with keratin can be good.

#### Jaggery

Jaggery is a traditional, unrefined sugar used throughout South and Southeast Asia. In one study, teak leaf extract as a natural dye, harda (*Terminalia chebula*), *Eucalyptus* (*Eucalyptus globulus*) leaves, Supari (*Areca catechu*), iron filings and jaggery, pomegranate peel (*Punica granatum* L.), tamarind (*Tamarindus indica*), and amla (*Phyllanthus emblica*) as bio-mordants were used to dye selected natural and synthetic fabrics. Depending on the bio-mordants, it has been claimed that colors with purple, pink, and burgundy tones can be created (Agrawal and Chopra 2020).

#### Lime

Lime is a type of white, inorganic-based binder that is obtained as a result of firing limestone at various temperatures (850–1450 °C), which, when mixed with water, solidifies in air or water depending on its type (Wikipedia 2023). It is seen that it is frequently used in natural dyeing work done in rural areas. In a different study, lime was used as a mordant in addition to metal mordant substances in the process of coloring wool fabrics with madder. It is stated that it gives higher color strength in post-mordanting with lime; brownish red color is obtained in post-mordanting; and red color is obtained in meta and pre-mordanting (Feiz and Norouzi 2014).

#### Lye

Başaran and Sarikaya (2015) used lye as a mordant in their studies. It is stated that the light fastness obtained while pre-mordanting cotton fabric samples with lye, yeast, whey, and mushroom extract is at a medium level, with the exception of whey.

#### Mud

Sludge has been used as a mordant agent in different studies on the coloring of textile materials with natural dyes. Most likely, the sludge content also varies depending on the regions where the sludge is obtained. Masae et al. (2017) used mud together with aluminum potassium sulfate, iron chloride, and sodium hydroxide to dye silk fabric.

#### Mushroom

Başaran and Sarikaya (2015) used mushroom as a mordant in their studies. It has been stated that khaki color tones can be obtained at the end of the process.

#### Oyster shells

Today, there are many different studies on the evaluation of seashells; however, here is an example of its use in natural dyeing that has been examined. In one study, extract from *Coreopsis drummondii* was used as a natural dye to dye silk, and lime juice from *Camellia japonica*, oyster shells, *Symplocos chinensis* (Lour) Druce for. pilosa (Nakai) Ohwi were used as natural mordants. It has been stated that the use of natural mordants increases the color’s strength (Kim et al. 2005).

#### Potassium acetate

Baig et al. (2019) used sodium citrate, ammonium acetate, and potassium acetate as mordants in their studies. According to the paper, among these three salts, sodium citrate showed superior fastness properties to other salts, while organic salts showed higher *K/S* ratios at lower concentrations.

#### Rare earth compounds

Another study, Zheng et al. (2011), used rare earth compounds (lanthanum-rich) as bio-mordants to dye ramie fabrics with natural dyes. It is reported that the colored fabrics showed greater color and shade stability when rare earth compounds were used as mordants. In other studies, “rare earth element” (Liu et al. 2011), “praseodymium chloride” (Liu and Bai 2012), and “montmorillonite,” (Liu and Chen 2012) as mordant, in cotton fabric-turmeric, silk fiber-monascorubrin pigment, and silk fabric-mugwort combinations, respectively, were used. It is stated that the dye absorption and *K/S* value of silk fabrics were successfully increased by mordanting with terrae rare (*Praseodymium chloride*) and monascorubrin pigment.

#### Sodium citrate

Baig et al. (2019) used sodium citrate, ammonium acetate, and potassium acetate as mordants in their studies. In the study, sodium citrate outperformed other salts in terms of its fastness properties, while organic salts displayed better *K/S* ratios at lower concentrations.

#### Sodium alginate

One of the rare bio-mordants is sodium alginate. Ghaheh et al. (2021) and Tehrani et al. (2023a) used sodium alginate as a bio-mordant. They used *Hibiscus sabdariffa* L extract as a natural dye, tannic acid, pine cone, lemon peel, and sodium alginate as bio-mordants to dye cotton fabrics. The findings show that sodium alginate and calcium lignosulfonate, along with the meta-mordanting technique, have produced the highest color strength.

#### Soy protein

**Fig. 10:**
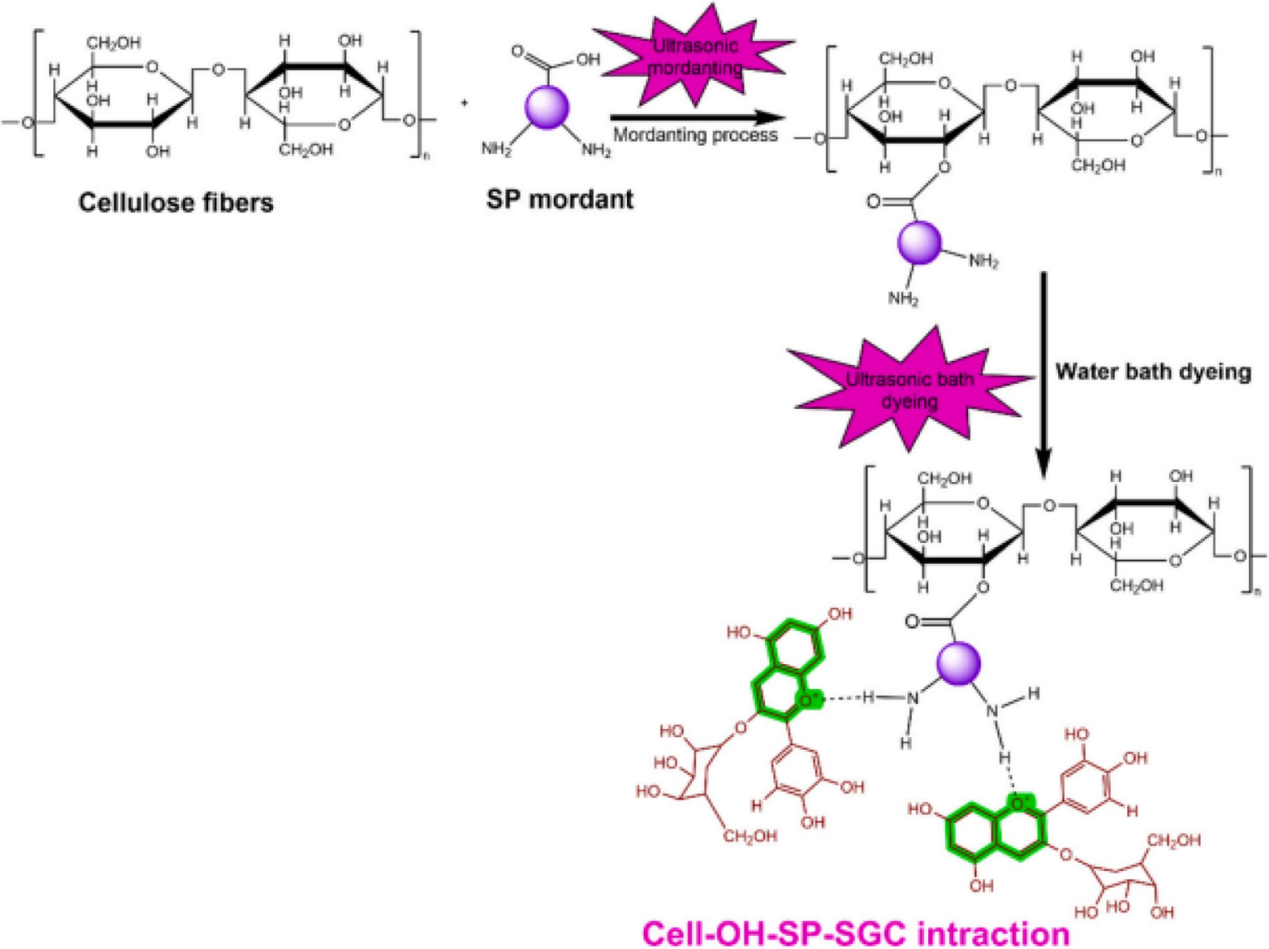
Interaction of cotton, natural dye, and bio-mordant (this figure was adapted from Periyasamy 2022)

Another interesting source of bio-mordant is soy protein. Periyasamy (2022) used *Syzygium cumini* fruit extract as a natural dye and soy protein as a bio-mordant to dye cotton fabric. The complex structure formed is presented in Fig. 10.

#### Urine

It is pointed out that traditionally, mordants were found in nature; wood ash or stale urine have been used as alkali mordants, and acids are found in acidic fruit or rhubarb leaves (which contain oxalic acid), for example (Ado et al. 2014).

#### Vinegar

Another type of bio-mordant that traditional natural dyers use is vinegar. In their research, Geetha and Sumathy (2013) used plant sources such as *Bougainvillea glabra*, beetroot, and red cabbage as natural dyes and vinegar as a bio-mordant to dye cotton fabrics. It has been stated that different colors can be obtained if vinegar is used as a mordant. In another study, coconut husk powder extract and vinegar as a bio-mordant were used to dye cotton fabric. It is stated that the rubbing, washing, and light fastness characteristics of the tested colored materials were found to be good (Kashyap et al. 2016).

#### Whey

**Fig. 11:**
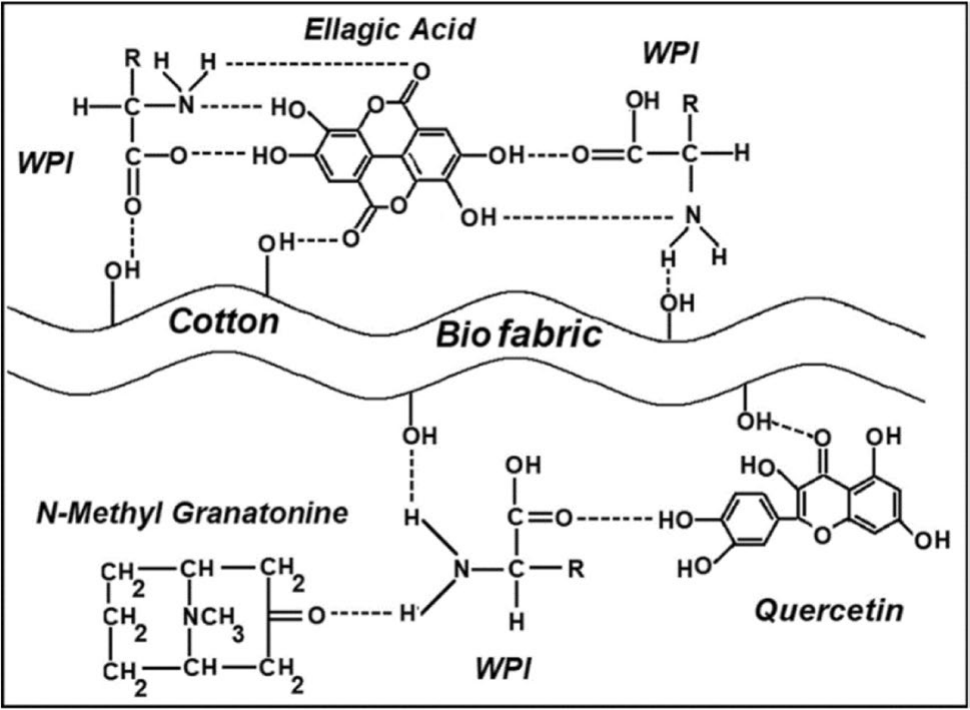
Diagram showing the chemical interactions between pomegranate rind dye, WPI, and cotton (this figure was adapted from Baseri 2020)

One of the different mordant substances used in natural dyeing is whey. Baseri (2020) used pomegranate peel as a natural dye and whey as a bio-mordant to dye cotton fabric. It has been stated that samples with sufficient color fastness and color strength can be obtained. The complex structure between the components is shown in Fig. 11.

#### Wood ash

Wood ash is one of the natural mordant materials that rural natural dyers frequently use. A study on the use of paddy ash as a natural mordant in natural dyeing is reviewed. Tajuddin et al. (2011) used *Xylocarpus moluccensis* heartwood extract as a natural dye and paddy ash as a natural mordant to dye viscose rayon. In one of these studies, Ahmad et al. (2012b) used *Melastoma malabathricum* L.’s (Senduduk) plant extract as a natural dye and wood ash as a natural mordant for dyeing silk fabrics. It has been stated that the natural mordants used increase the color fastness values. Geelani et al. (2017) used a natural dye extract from *Quercus robur* L. (fruit cups) to dye wool, cotton, silk, and pashmina fabrics in their research and wood ash obtained from *Salix alba* L. and *Populus deltoides Bartram ex marsh* as a bio-mordant. It is reported that with *Populus deltoides* (wood ash) mordant on the pashmina fabric, which used the pre-mordanting procedure, the highest value of the percent absorption was discovered. Wood ash has also been commonly used in natural mordanting processes in Indonesia, particularly *Sterculia foetida* L. (fruit peels), *Ixora coccinea* L., *Schleichera oleosa* Merr., or *Tamarindus indica* L. (wood) and the ashes of these plants (Cunningham et al. 2011).

#### Yeast

One of the interesting mordant substances used in natural dyeing is yeast. Başaran and Sarikaya (2015) used *Quercus infectoria* as a natural dye to dye cotton fabrics and whey, yeast, lye, and mushroom extract as bio-mordants in their research. It is claimed that, with the exception of whey, the light-fastness produced after pre-mordanting cotton fabric samples with lye, yeast, whey, and mushroom extract is at a medium level.

#### Hyperbranched polymers

In this form of dendrimer application, dye molecules connect with the end groups, branches, or core of a dendrimer molecule. The interaction causes a change in the dye’s absorption or emission behavior, which can reveal details about the structure of the dendrimer or how it interacts with its surroundings (Froehling 2001). Walnut bark (*Juglans regia* L.), oak tree (*Quercus infectoria*), chamomile (*Anthemis tinctoria*), sage (*Salvia officinalis* L.), and buckthorn (*Rhamnus petiolaris*) as natural dye sources, and polypropylene with the addition of hyperbranched polymer (HBP) by melt spinning technique, it has been reported that PP fiber can be dyed. It is stated that the addition of HBP improved the ability of PP fibers to take color from natural sources (Davulcu 2015). In another study, madder as a natural dye and chitosan-polypropylene imine dendrimer hybrid (CS-PPI) was used as a bio-mordant for dyeing wool. It is stated that CS-PPI can be employed as a promising antibacterial finishing ingredient for the creation of hygienic colored wool textiles as well as an alternative “bio-mordant” in place of the metallic mordant typically used in wool dyeing with madder dye (Mehrparvar et al. 2016). Sadeghi-Kiakhani et al. (2022a) used cochineal and madder as natural dyes and a chitosan-poly (amidoamine) dendrimer (Ch-PAMAM) hybrid as a bio-mordant to dye wool yarn. It is stated that the Ch-PAMAM hybrid can be used successfully as a green potential agent for multifunctional wool treatment with its improved natural dyeing quality and antibacterial and antioxidant properties. Additionally, it has been reported by some researchers that the dyeing abilities of cotton fabrics can be improved by modifying their surfaces with some polymers (e.g., poly (4-vinyl pyridine), quaternary ammonium salts, polyethyleneimine, polyamine dendrimers) (Zhang et al. 2022c).

#### Enzymes

**Fig. 12:**
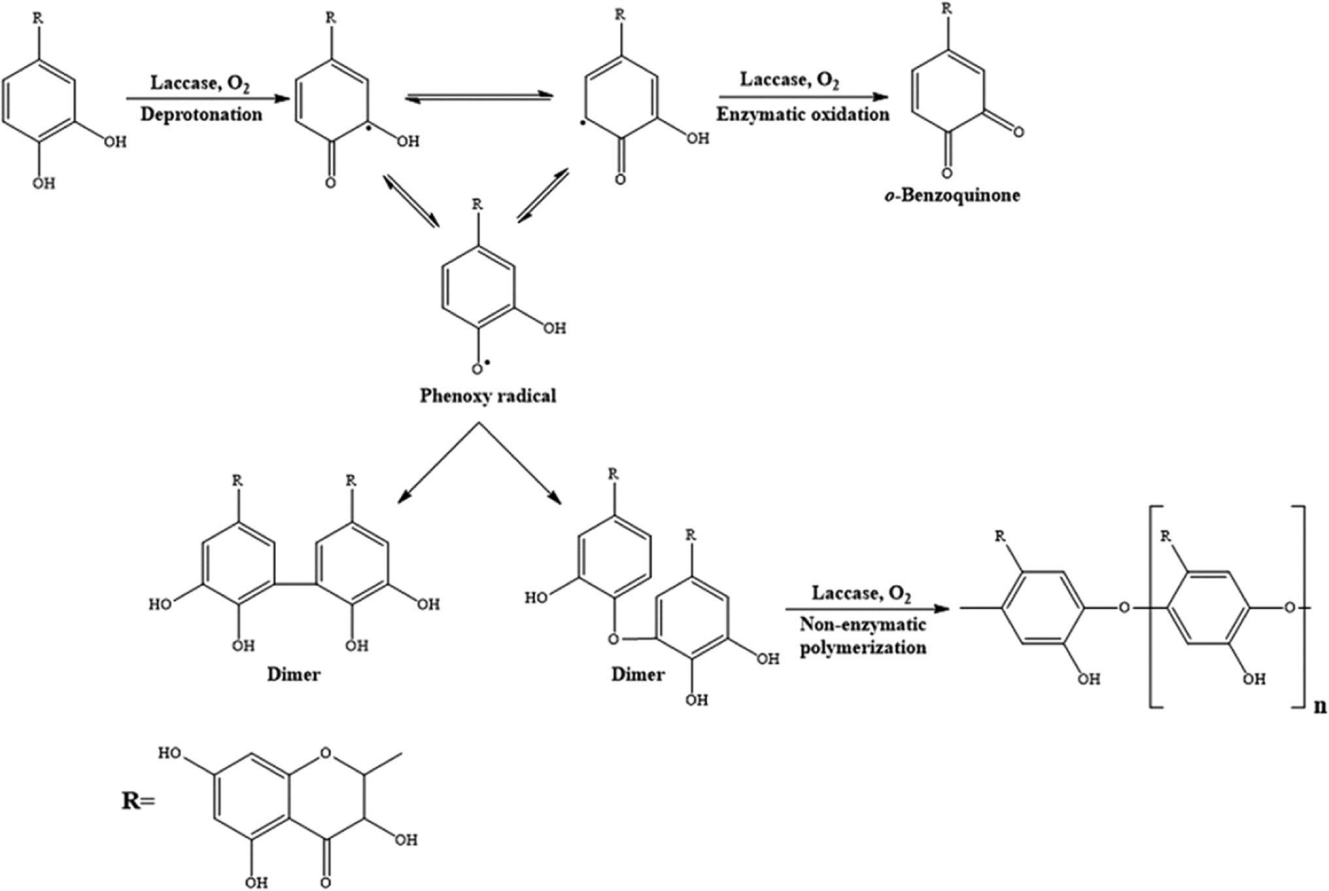
Quercetin’s conversion to o-benzoquinone and laccase’s polymerization of it (this figure was adapted from Atav et al. 2022)

**Fig. 13:**
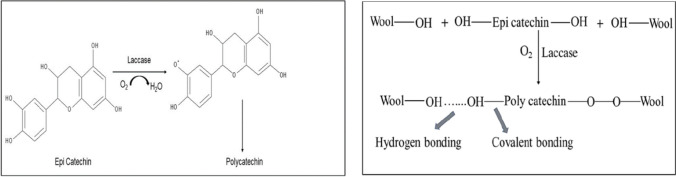
The mechanism of laccase-assisted wool dyeing using green tea extract (this figure was adapted from Garg et al. 2023)

Because of their flexibility, reliability, and energy and water savings, enzymes are gaining an increasing role in textile wet processes. In recent years, novel recombinant and/or bioengineered enzymes, such as cellulases, have been added to the process of processing and finishing textiles in dye factories. A number of researchers are experimenting with enzymatic treatment of cotton and wool fabric to enhance softness and surface appearance, speed up the dyeing process, and other purposes (Vankar and Shanker 2008a). It has been found that enzymes are also used in natural dyeing processes. Some of these enzymes are as follows: laccase, protease, amylase, cellulase, diasterase, xylanase, and m-TGase. For example, Dumitrescu et al. (2012) mentioned in their study that some enzymes (cellulose, protease, and amylase) instead of metal salts could increase the dye yield. Sricharussin et al. (2009) cleaned the pineapple leaf fibers with pectinase and cellulase enzymes before dyeing and then dyed them with five different natural dyes. Accordingly, it has been reported that it can be effective for dyeing pineapple leaf fibers with natural material without resorting to potentially toxic mordants. It is emphasized that enzyme treatments effectively remove non-celulosic materials, and this results in a hyrophilic surface, and improved wettability, and may have dyeability. In one study, the tannic acid-enzyme (protease-amylase, diasterase, and lipase)-natural dye (*Terminalia arjuna*, *Punica granatum*, and *Rheum emodi*) combination method offered an environmentally friendly alternative to metal-mordanted natural dyeing. According to reports, the enzymes are adsorbed onto the silk fabric through a variety of ionic and nonionic modes of attraction, including electrostatic forces, hydrogen bonding, and dipole–dipole interactions. In order to prevent the dye from washing out, an enzyme-dye complex is created on the surface of the colored silk cloth (Vankar and Shanker 2009; Vankar et al. 2007). Another enzyme used in natural dyeing processes is laccase. It is stated that a multicopper oxidase known as laccase catalyzes the oxidation of phenolic compounds by transferring a single electron, which results in the oxidation of oxygen to water. Tyrosine is found in wool, and it can interact with natural colors through covalent bonding through laccase-assisted polymerization to produce effects that are washable and multifunctional (Garg et al. 2023). In a study, laccase enzyme was used instead of harmful mordants in the fixation of three different flavonoids (natural dyes), rutin, morin, and quercetin, and high color fastness was obtained in fabric printing. In explaining the formation of quinone derivatives in the structure of natural dyes with the laccase enzyme, El-Hennawi et al. (2012) stated as follows: They stated that the laccase reaction probably proceeds with the formation of a radical cation, and deprotonation of the hydroxyl group occurs to give a subsequent radical. That is, according to the statement, the laccase processes most likely start with the creation of a radical cation, which is followed by the hydroxyl group’s deprotonation to produce a radical. After that, the radical can transform into a derivative of quinonoid. Bai et al. (2016) used Chinese gallnut, turmeric, and grape seed extract as natural dyes and the enzyme laccase as a mordant agent to dye wool fabrics. The outcomes demonstrated that laccase had a significant impact on Chinese gallnut dyeing performance. The chemical interaction and binding mechanism of the laccase enzyme between phenolic hydroxyl-based natural dyes and wool are explained as follows: It has been reported that the phenolic hydroxy (-OH) group of natural dyes turns into quinones by oxidation of the laccase enzyme, and then the reaction proceeds by spontaneously forming covalent bonds under the catalyst of laccase. Alves et al. (2022) used madder as a natural dye, Quebracho tree sap (*Schinopsis spp*.), and laccase enzyme as bio-mordants to dye cotton fabric. Atav et al. (2022) used laccase-catalyzed enzymatic dye synthesis to dye cotton fabrics with St. John’s wort and white onion peel extracts as an alternative to traditional mordants. They explained the function of the enzyme in the dyeing medium as follows: The phenolic active chemicals in the natural dyes’ structures alter structurally when laccase enzyme is present in the surrounding environment during dyeing. It is likely that the laccase processes start with the production of a radical cation, which is followed by the hydroxyl (-OH) group being deprotonated to produce a radical. The radical may then proceed via quinonoid derivative production (Fig. 12). Enzymes cause phenolic chemicals to change into polymeric compounds, which increases the molecular weight of the former. It indicates that the natural dyes produce darker colors when enzymes are present. Because of their strong reactivity, quinones have the ability to spontaneously polymerize into molecules with large molecular weights. One study used green tea (*Camellia sinensis*) as a natural dye to create multipurpose wool fabric. In situ polymerization of *Camellia sinensis* phenolic compounds on wool was accomplished using the enzyme laccase. It is reported that an increase in the enzyme’s uses led to more polymeric dyes based on the phenolic components of natural dyes, which resulted in a striking improvement in the dyed wool materials’ color strength (*K/S*). Green tea includes polyphenolic chemicals, which the laccase enzyme can oxidize inside the amorphous area of wool fiber. Through the polymerization of phenolic chemicals, it was demonstrated that natural colors can be fixed to wool without the use of a mordant. Epicatechin, a polyphenolic molecule found in green tea, has been polymerized using laccase, as reported in the literature. It is said that the dyed wool fabrics had strong dye-fiber interactions because polymeric dyes and wool formed a greater number of strong covalent and hydrogen bonds (Garg et al. 2023). Figure 13 shows the proposed reaction strategy.

The current approach uses an enzyme to dye wool sustainably and naturally without using metal mordants, which can have hazardous effects. Here, it is stated that dyeing occurs by forming hydrogen and covalent bonds in the presence of laccase enzyme and oxygen through the hydroxyl (-OH) groups of wool and catechin (Fig. 12) (Garg et al. 2023).

In a study, it was reported that polyamide fiber could be dyed with sufficient fastness using pomegranate peels and walnut barks as natural dye sources after being modified with pepsin and trypsin enzymes (Bahtiyari and Benli 2016). In another study, Vankar and Shanker (2008a, 2009) reported that more efficient dyeing can be obtained when cotton fabric pretreated with some enzymes (protease and amylase, lipase, and diasterase) is dyed with selected natural dyes such as *Acacia catechu* and *Tectona grandis*. In one study, natural pigment obtained from sappan was used for dyeing wool fabrics after treatment with protease and transglutaminase. It has been emphasized that the enzymatically modified wool fabric has sufficient fastness (Zhang and Cai 2011). They explained the relationship between natural dyes and enzyme-wool dyeing as follows: Wool samples that have previously been treated with hydrogen peroxide are then treated with protease and transglutaminase. Protease catalyzes the proteolytic reaction to the wool’s scales, breaking the disulfide crosslink and allowing more dyes to permeate the treated wool fibers. Nazari et al. (2014) emphasized that wool fabric has high dye absorption when dyed with natural dyes (madder and cochineal) after pretreatment with the denture enzyme. The enzyme-dye relationship is expressed as the dye affinity increasing due to the protease enzyme hydrolyzing some peptide bonds in the protein molecule. In a study, *Rubia cordifolia* extract was used as a natural dye to dye silk fabric, and different enzymes (protease, amylase, xylanase, pectinase, phytase) as a mordant were used simultaneously and in two stages. It is reported that protease was the finest choice for the one-step dying procedure, and the reactivity of the enzymes was as follows: protease > phytase > xylanase > amylase > pectinase. It is stated that *Rubia* can be used as a dye source in enzymatic treatment to produce silk fabric with good color strength and has good commercial dyeing potential. They stated that hydrogen bonds, dipole–dipole interactions, and electrostatic forces are some of the ionic and non-ionic factors of attraction that cause the enzymes to be adsorbed onto the silk fabric. Additionally, they reported that since the majority of the material in silk fibers is proteinaceous, with starches and pectins serving as binding agents, commercially available enzymes such as protease, amylase, and pectinase have been used to loosen the surrounding material, improving dye molecules’ absorption into the fabric under milder conditions (Vankar et al. 2017). In one study, madder was used as a natural dye, and alum and the transglutaminase (m-TGase) enzyme were used as mordants for dyeing wool fabric (Pour and He 2020a). In another study, Pour et al. (2020b) used m-TGase and bentonite as bio-nano-mordants for dyeing wool. They modified the surface of the wool by adding the appropriate amount of m-TGase due to the cross-linking reaction between the two main amino acids of the wool, glutamine and lysine. The inhomogeneous surface of the obtained hydrophilic wool interacted with the hydrophilic bentonite and thus provided a rough surface for the placement of dye molecules during the dyeing process. It was stated that the surface modification significantly affects the obtained color strength (Pour et al. 2020b). It is seen that some enzymes can be used in natural dyeing processes, but it would be more appropriate to classify them as auxiliary substances that can increase the color yield of textile materials rather than directly calling them mordants.

### Conventional mordants (metal salts)

A fixer is needed to bind the dye to the fiber because the majority of natural dyes have a relatively poor affinity for textile fibers (Iqbal and Ansari 2021). Mordants are substances that form complexes and contain metal ions. That is, a highly intricate chemical structure develops between the dye, the fiber, and the mordant material during the natural dyeing process. The authors have put forward different theories to explain this complex structure (Kasiri and Safapour 2013; Shabbir et al. 2017; Singh et al. 2019; Baseri 2020; Zhang et al. 2021; Thakker and Sun 2022; Shahmoradi Ghaheh et al. 2023). For example, a complex chemical structure is formed through the amino and carboxylic groups of wool and the hydroxy groups of phenolic groups of plant structures. Thus, the dyeing of the fiber takes place. Mordants are used to alter the color, shade, and fastness properties of dyes as well as to improve dye uptake and fixation. The three mordanting techniques and the type of mordant have a considerable impact on color strength and color coordination (Tang et al. 2010; Benli and Bahtiyari 2015a, b; Jabar et al. 2020, 2021). Heavy metal ions used as mordants include copper, iron, chromium, cobalt, nickel, and aluminum, whose residue in dyeing wastewater causes major problems with effluent disposal (Kasiri and Safapour 2013). Both the natural dyes with ligands such as -OH, -NH_2_,—COOH groups, and the textile fibers with active sites such as –NH_2_, -COOH groups can make a complex with a metal ion of the metallic mordant thereby enhancing the extent of fabric-mordant-dye interactions (Ibrahim et al. 2013). Recently, the most important source of concern in natural dyeing circles is the harm that metal-mordant substances can cause to the environment such as chromium. Residual heavy metals and all dyestuffs should conform to the Ecological and Toxicological Association of the Dyestuff Manufacturing Industry (ETAD) regulations. Limits for tracing some metals in compounds have been proposed by ETAD as follows: chromium (Cr) 100 ppm, silver (Ag) 100 ppm, nickel (Ni) 200 ppm, tin (Sn) 250 ppm, copper (Cu) 250 ppm, zinc (Zn) 1500 ppm, iron (Fe) 2500 ppm (Roy Choudhury 2013). Since the main target of this review is not metallic mordants, the research made with these mordants is not mentioned.

## Outlooks for the future

Although the use of natural dyes has advanced tremendously, much more work has to be done before plant-based colorants and bio-mordants can be considered competitive alternatives to their synthetic equivalents. More study is needed on the toxicity, safety, and quality of colorants made from plants and bio-mordants before they may be used commercially. Poor fastness qualities, long extraction processes, and non-reproducibility are the main problems faced by dyers and finishers using natural materials in textile work. To these difficulties can also be added the environmental pressure of metal mordants, which significantly affects the color tone. In the future, it is seen as a great possibility that the use of natural resources will increase and become widespread in the process of coloring textile materials by overcoming the abovementioned difficulties without harming the environment.

During the research, although it was observed that plant materials were used more as mordant substances compared to other mordants, it was determined that different materials were used as mordants. Particularly, new-generation and non-vegetable-based mordants have a high potential for natural dyeing processes. Because nature offers incredibly different materials to human beings, it is thought that these will be brought to light with studies carried out over time. Additionally, due to the potential of oil-based plant materials to be used in foodstuffs, they are thought to have limited use and development as mordants.

## Conclusions

Sustainability is the practice of avoiding the depletion of natural resources in order to maintain ecological balance and preserve the standard in living of society. Natural dyes gain importance again in the coloring processes of textile materials for the textile dye industry. In this context, the sustainability of natural dyeing processes is an important issue. Large-scale use of natural dyes promotes sustainable development, which satisfies existing needs without jeopardizing the ability of future generations to meet their own. The economy, society, and the environment are the three pillars of sustainability. The relationship between the use of natural dyes and sustainability is then emphasized by the three fundamental principles of sustainable development: profit, people, and planet. To address climatic challenges and restore sustainability, natural dyes provide some valuable benefits for the environment, economy, and society (Iqbal and Ansari 2021). In this respect, it is important not to use metal salts that are harmful to the environment when using natural dyes. Bio-mordants are biological natural materials having metal ion(s), tannins, etc. that mostly come from vegetable sources and act as mordants in natural dyeing processes. Some plants and plant parts with high tannin or metal content may present mordanting effects to various extents depending on their chemical structure and the amount of metal present in them (Rather et al. 2016). Researchers have advised the use of natural mordants (bio-mordants) in place of metallic salt mordants in terms of an effective and safe alternative considering environmental aspects of pollution and their biodegradable nature; hence, they can be discharged into the environment without any chemical or physical treatment (e.g., precipitation or filtration).

Until the discovery of synthetic dyestuffs, human beings used materials obtained from natural sources as dyestuffs. After the discovery of synthetic dyestuffs, natural dyestuffs continued to be used, albeit partially. In this context, the most preferred natural resource has been plants because access to plant resources is quite easy and cheap. In addition, they do not have harmful effects on the environment. In particular, vegetable sources were used as a natural dye source before being used as a bio-mordant material. It is possible to find many academic studies on this subject. In light of the technological developments in recent years, it is predicted that some plant resources can also be used as bio-mordants. It is likely that in the coming years, many different herbal sources will be used both as dyestuffs and as bio-mordants. In many parts of the world, it has been found that herbal resources are used in natural dyeing. This diversity comes to the fore in countries with a very large plant flora, especially in the Eurasian Belt. Starting from rare plants that grow depending on the region (Asia, Europe, etc.) and climatic conditions, there are common plants that spread to very wide geographies, and these have found use in natural dyeing.

Recently, a large number of scientists have been researching and developing new and effective bio-mordants to replace synthetic mordants. As a result of the examination, the following conclusions can be clearly expressed:

1. A total of 84 different herbal sources were used as bio-mordants.
2. There are 30 different types of new-generation and non-vegetable-based mordants.
3. The first traces of the use of herbal mordants date back to the 1990s.
4. In the last 5 years, herbal resources have been extensively used as bio-mordants.
5. Most of the vegetable sources were used both as a dye and a mordant.
6. It was determined that herbal-based bio-mordants are mostly used in Asian countries. It is thought that the reason for this situation may be due to its rich vegetation.
